# Timing of Allergenic Food Introduction and Risk of Immunoglobulin E–Mediated Food Allergy: A Systematic Review and Meta-analysis

**DOI:** 10.1001/jamapediatrics.2023.0142

**Published:** 2023-03-27

**Authors:** Roberta Scarpone, Parisut Kimkool, Despo Ierodiakonou, Jo Leonardi-Bee, Vanessa Garcia-Larsen, Michael R. Perkin, Robert J. Boyle

**Affiliations:** 1 School of Public Health, Imperial College London, London, United Kingdom; 2 Department of Paediatric Allergy, Imperial College Healthcare NHS Trust, London, United Kingdom; 3 Department of Primary Care and Population Health, University of Nicosia Medical School, Nicosia, Cyprus; 4 Centre for Evidence-Based Healthcare, School of Medicine, University of Nottingham, Nottingham, United Kingdom; 5 Department of International Health, Johns Hopkins University, Baltimore, Maryland; 6 Population Health Research Institute, St George’s, University of London, London, United Kingdom; 7 National Heart and Lung Institute, Imperial College London, London, United Kingdom

## Abstract

**Importance:**

Earlier egg and peanut introduction probably reduces risk of egg and peanut allergy, respectively, but it is uncertain whether food allergy as a whole can be prevented using earlier allergenic food introduction.

**Objective:**

To investigate associations between timing of allergenic food introduction to the infant diet and risk of food allergy.

**Data Sources:**

In this systematic review and meta-analysis, Medline, Embase, and CENTRAL databases were searched for articles from database inception to December 29, 2022. Search terms included *infant*, *randomized controlled trial*, and terms for common allergenic foods and allergic outcomes.

**Study Selection:**

Randomized clinical trials evaluating age at allergenic food introduction (milk, egg, fish, shellfish, tree nuts, wheat, peanuts, and soya) during infancy and immunoglobulin E (IgE)–mediated food allergy from 1 to 5 years of age were included. Screening was conducted independently by multiple authors.

**Data Extraction and Synthesis:**

The Preferred Reporting Items for Systematic Reviews and Meta-analyses guideline was used. Data were extracted in duplicate and synthesized using a random-effects model. The Grading of Recommendations, Assessment, Development, and Evaluation framework was used to assess certainty of evidence.

**Main Outcomes and Measures:**

Primary outcomes were risk of IgE-mediated allergy to any food from 1 to 5 years of age and withdrawal from the intervention. Secondary outcomes included allergy to specific foods.

**Results:**

Of 9283 titles screened, data were extracted from 23 eligible trials (56 articles, 13 794 randomized participants). There was moderate-certainty evidence from 4 trials (3295 participants) that introduction of multiple allergenic foods from 2 to 12 months of age (median age, 3-4 months) was associated with reduced risk of food allergy (risk ratio [RR], 0.49; 95% CI, 0.33-0.74; *I*^2^ = 49%). Absolute risk difference for a population with 5% incidence of food allergy was −26 cases (95% CI, −34 to −13 cases) per 1000 population. There was moderate-certainty evidence from 5 trials (4703 participants) that introduction of multiple allergenic foods from 2 to 12 months of age was associated with increased withdrawal from the intervention (RR, 2.29; 95% CI, 1.45-3.63; *I*^2^ = 89%). Absolute risk difference for a population with 20% withdrawal from the intervention was 258 cases (95% CI, 90-526 cases) per 1000 population. There was high-certainty evidence from 9 trials (4811 participants) that introduction of egg from 3 to 6 months of age was associated with reduced risk of egg allergy (RR, 0.60; 95% CI, 0.46-0.77; *I*^2^ = 0%) and high-certainty evidence from 4 trials (3796 participants) that introduction of peanut from 3 to 10 months of age was associated with reduced risk of peanut allergy (RR, 0.31; 95% CI, 0.19-0.51; *I*^2^ = 21%). Evidence for timing of introduction of cow’s milk and risk of cow’s milk allergy was very low certainty.

**Conclusions and Relevance:**

In this systematic review and meta-analysis, earlier introduction of multiple allergenic foods in the first year of life was associated with lower risk of developing food allergy but a high rate of withdrawal from the intervention. Further work is needed to develop allergenic food interventions that are safe and acceptable for infants and their families.

## Key Points

**Question:**

Is the timing of introduction of allergenic foods to infants associated with their risk of developing immunoglobulin E–mediated food allergy?

**Findings:**

This systematic review and meta-analysis of 23 randomized clinical trials with 13 794 participants found moderate-certainty evidence that introducing multiple allergenic foods from 2 to 12 months of age was associated with reduced risk of any food allergy but increased risk of withdrawal from the intervention. There was high-certainty evidence that earlier introduction of egg or peanut was associated with reduced risk of egg or peanut allergy, respectively.

**Meaning:**

In this study, earlier introduction of multiple allergenic foods was associated with a reduced risk of food allergy but with significant rates of withdrawal from the intervention.

## Teaser

This systematic review and meta-analysis examines whether introducing allergenic foods to infants aged 2 to 12 months is associated with risk of developing immunoglobulin E–mediated food allergy at 1 to 5 years of age.

## Introduction

Food allergy is an important public health concern due to significant quality-of-life and economic impact.^1^ Food allergy incidence varies between populations, and the discovery that earlier introduction of egg and peanut to the infant diet probably reduces risk of egg and peanut allergy, respectively, has galvanized primary prevention efforts.^2,3,4^ Clinical practice guidelines now recommend earlier introduction of allergenic foods, and infant feeding practice has changed substantially in some regions.^5,6,7^ It is unknown whether earlier introduction of allergenic foods will reduce overall food allergy prevalence in populations.^8^ Prevention of 1 or 2 specific food allergies without preventing food allergy as a whole may have limited public health impact. One trial evaluating the effect of earlier multiple allergenic food introduction on risk of any food allergy reported inconclusive findings and a high rate of withdrawal from the intervention.^9^ A more recent trial reported reduced food allergy following earlier introduction of multiple allergenic foods, largely attributable to a reduction in peanut allergy.^10^

Herein, we report outcomes from a targeted update of a previous systematic review conducted by some of us of the timing of allergenic food introduction and atopic or autoimmune disease.^2^ We evaluated the association between earlier allergenic food introduction and risk of any food allergy and evaluated rates of withdrawal from the intervention as a marker of safety and acceptability of earlier allergenic food introduction.

### Methods

The methods of this systematic review and meta-analysis are described in detail in the eMethods in Supplement 1. Protocol and search strategies were registered in PROSPERO (CRD42013004239).^11^ This study was reported according to the Preferred Reporting Items for Systematic Reviews and Meta-analyses (PRISMA) reporting guideline.^12,13^ We searched the Medline, Embase, and CENTRAL databases for articles published from database inception to December 29, 2022. Search terms included *infant*, *randomized controlled trial*, and terms for common allergenic foods and allergic outcomes (eAppendix in Supplement 1). Screening was conducted independently by 2 of us (R.S. and P.K.). Reference lists of relevant included studies were reviewed to identify additional potentially eligible studies. We included randomized clinical trials evaluating age at allergenic food introduction (milk, egg, fish, shellfish, tree nuts, wheat, peanuts, and soya) during the first year of life and immunoglobulin E (IgE)–mediated food allergy at age 1 to 5 years. The study populations were infants enrolled from birth to 12 months of age. Studies that compared earlier and later allergenic food introduction and different doses and types of exposure were included, as were trials using breastfeeding or breastmilk, amino acid formula, other low-allergen exposures, or standard care as the comparator. We excluded nonrandomized trials, trials of timing of solid food introduction that did not use allergenic foods, and trials in specific populations, such as very premature infants.

### Outcomes

The primary efficacy outcome was IgE-mediated allergy to any food assessed by double-blind, placebo-controlled food challenge; open food challenge; medical diagnosis; or parental report at the closest reported time point to age 3 years. The primary safety outcome was withdrawal from study intervention assessed as the number of randomized study participants in each group who withdrew from the intervention or were lost to follow-up during the intervention period for reasons related to the intervention or reasons that could potentially have been related to the intervention. Secondary outcomes included allergic sensitization to any food assessed by a positive result of a skin prick test (SPT) and/or allergen-specific IgE test as well as allergy and allergic sensitization to 1 of the aforementioned specific common allergenic foods.^14^

### Statistical Analysis

Data were extracted in duplicate, and risk of bias was assessed using the Cochrane Risk of Bias 2 tool.^15^ Publication bias was assessed using funnel plots and the Egger test when 10 or more trials were included in a meta-analysis.^16^ Results are presented as risk ratios (RRs) with 95% CIs and expressed as risk differences where possible. Random-effects meta-analyses used the DerSimonian and Laird method in the metafor version 3.4-0 package in R, version 4.2.0 (R Project for Statistical Computing).^17,18,19,20^ Statistical significance was set at 2-sided *P* < .05, and heterogeneity was quantified using the *I*^2^ statistic. Data that could not be included in the meta-analysis were reported narratively. Prespecified subgroup analyses were conducted using study-level variables, comparing single vs multiple allergenic food introduction, high vs low allergen intake, and milk feeding status at enrollment. The prespecified sensitivity analysis evaluated low-risk-of-bias data only. Post hoc trial sequential analysis was used to quantify statistical reliability of key moderate- or high-certainty efficacy outcomes.^21^ Control event rates pooled from the largest included studies (5% for any food allergy, 4% for egg allergy, and 2.5% for peanut allergy) were used to estimate optimal heterogeneity-adjusted information sizes needed to identify a risk reduction of 30%, assuming a 2-sided *P* < .05 significance level and 80% power. The Grading of Recommendations, Assessment, Development, and Evaluation (GRADE) framework was used to assess certainty of evidence.^22,23^ The data set and statistical code are available from the corresponding author.

## Results

The results of the search and selection process are presented in a PRISMA flow diagram (eFigure 1 in Supplement 1). From a total of 9283 titles, 23 trials reported in 56 articles were included in the review.^3,4,9,10,24,25,26,27,28,29,30,31,32,33,34,35,36,37,38,39,40,41,42,43,44^ The characteristics of the 23 studies^3,4,9,10,24,25,26,27,28,29,30,31,32,33,34,35,36,37,38,39,40,41,42,43,44^ (13 794 randomized participants) and 12 ongoing studies (16 765 intended participants) are given in eTables 1 and 2 in Supplement 1. Twenty-one studies (91%)^3,4,9,10,24,25,26,27,28,30,31,32,33,34,35,36,37,38,39,40,41,43,44^ were conducted in high-income countries. Interventions were multiple allergenic foods (4 studies^9,10,28,37^), egg (9 studies^24,27,29,33,36,38,39,42,43^), peanut (1 study^3,4^), cow’s milk (8 studies^25,26,30,31,32,34,35,41,44^), and a comparison of all these interventions (1 study^40^). Twelve^3,4,9,10,24,27,28,36,37,38,39,40,43^ of 15^3,4,9,10,24,27,28,29,33,36,37,38,39,40,42,43^ complementary feeding trials (80%) initiated the intervention prior to age 6 months. A summary of key findings, with GRADE evidence assessments for earlier multiple foods, egg, peanut, and cow’s milk introduction, is presented in the Table, with further detailed findings reported in eTables 3 to 6 in Supplement 1. Results of subgroup and sensitivity analyses for these comparisons are given in eTables 7 to 11 in Supplement 1, and outcomes for earlier introduction of other foods are shown in eTable 12 in Supplement 1.

**Table.** Summary of Key Review Findings for Earlier vs Later Introduction of Allergenic Foods to the Infant Diet

Intervention, outcome	Participants, No. (studies, No.)	RR (95% CI)	Certainty of evidence^a^	Control risk, cases per 1000 population^b^	RD (95% CI), cases per 1000 population	NNTB/H (95% CI)
**Earlier introduction of multiple allergenic foods**						
Allergy to any food	3295 (4^9,10,37,40^)	0.49 (0.33 to 0.74)	Moderate	50	−26 (−34 to −13)	38 (29 to 77)
				200^c^	−102 (−134 to −52)	10 (7 to 19)
Withdrawal from study intervention	4703 (5^9,10,28,37,40^)	2.29 (1.45 to 3.63)	Moderate	200	258 (90 to 526)	4 (2 to 11)
**Earlier egg introduction**						
Allergy to egg	4811 (9^9,10,24,29,36,37,38,39,43^)	0.60 (0.46 to 0.77)	High	40	−16 (−22 to −9)	63 (45 to 111)
				200^c^	−80 (−108 to −46)	13 (9 to 22)
Withdrawal from study intervention	7442 (13^9,10,24,28,29,33,36,37,38,39,40,42,43^)	1.58 (1.12 to 2.22)	Low	200	116 (24 to 244)	9 (4 to 42)
**Earlier peanut introduction**						
Allergy to peanut	3796 (4^3,9,10,37^)	0.31 (0.19 to 0.51)	High	25	−17 (−20 to −12)	59 (50 to 83)
				100^c^	−69 (−81 to −49)	14 (12 to 20)
Withdrawal from study intervention	5343 (6^3,9,10,28,37,40^)	1.91 (1.19 to 3.05)	Very low	200	182 (38 to 410)	5 (2 to 26)
**Earlier cow’s milk introduction**						
Allergy to cow’s milk	3900 (6^9,10,31,32,37,44^)	0.84 (0.38 to 1.87)	Very low	10	−2 (−6 to 9)	500 (110 to ∞)
				50^c^	−8 (−31 to 44)	125 (23 to ∞)
Withdrawal from study intervention	7895 (11^9,10,25,26,28,31,32,37,40,41,44^)	1.05 (0.61 to 1.82)	Low	200	10 (−78 to 164)	100 (6 to ∞)

Abbreviations: NNTB/H, number needed to treat for an additional beneficial or harmful outcome; RD, risk difference; RR, risk ratio.

^a^ Grading of Recommendations, Assessment, Development, and Evaluation framework.

^b^ Control event rate was pooled from the largest included studies.

^c^ Population at high risk for developing food allergy.

### Earlier Introduction of Multiple Allergenic Foods

**Figure 1.**
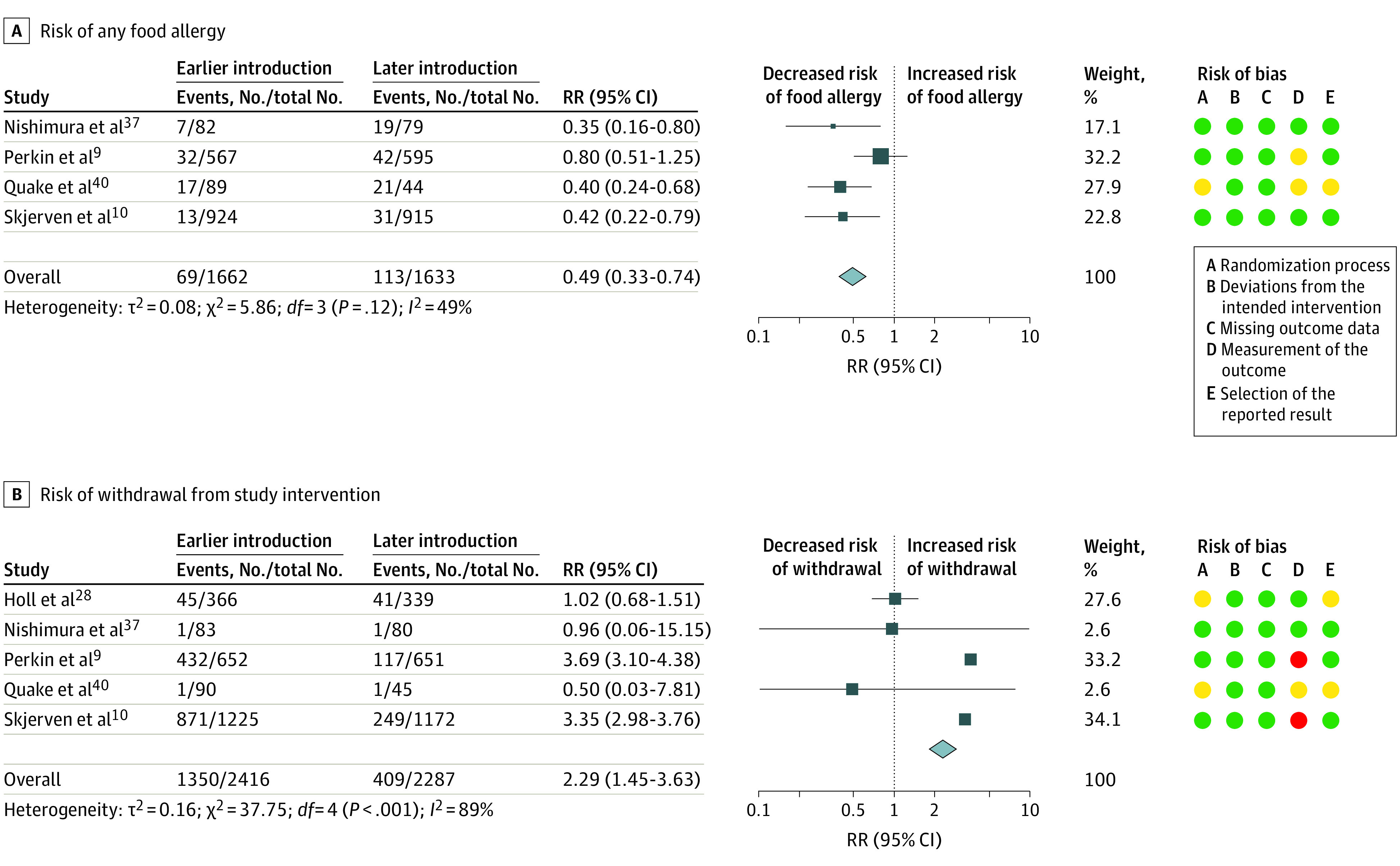
Earlier vs Later Introduction of Multiple Allergenic Foods and Risk of Any Food Allergy and Withdrawal From Study Intervention Squares indicate risk ratios (RRs), with horizontal lines indicating 95% CIs and size of squares indicating weight; diamonds indicate pooled estimates, with outer points of the diamonds indicating 95% CIs. Green circles indicate low risk of bias; yellow, some concerns; and red, high risk of bias.

Meta-analysis of 4 trials^9,10,37,40^ (3295 participants) showed moderate-certainty evidence that earlier introduction of multiple allergenic foods between ages 2 and 12 months (median age, 3-4 months) was associated with decreased risk of any food allergy from 1 to 3 years of age (RR, 0.49; 95% CI, 0.33-0.74; *I*^2^ = 49%) (Figure 1A). Statistical heterogeneity was explained by a less pronounced effect in 1 large study.^9^ The reason for the different findings in this study was not clear, and certainty of evidence was therefore downgraded for inconsistency. Absolute risk difference for a population with 5% incidence of food allergy was −26 cases per 1000 population (95% CI, −34 to −13 cases per 1000 population). Trial sequential analysis showed that the heterogeneity-adjusted optimal information size for detection of a 30% risk reduction had not been reached (eFigure 2 in Supplement 1). There was low-certainty evidence for earlier introduction of multiple allergenic foods and risk of any food sensitization (3 trials^9,10,37^ [2827 participants]; RR, 0.77; 95% CI, 0.54-1.10; *I*^2^ = 73%) (eFigure 3 in Supplement 1). Certainty of evidence was downgraded for inconsistency and imprecision. There was moderate-certainty evidence that earlier introduction of multiple allergenic foods was associated with risk of withdrawal (5 trials^9,10,28,37,40^ [4703 participants]; RR, 2.29; 95% CI, 1.45-3.63; *I*^2^ = 89%) (Figure 1B). Statistical heterogeneity was explained by high rates of withdrawal from the intervention in the 2 largest studies,^9,10^ which both used high allergen intake for multiple allergenic foods and normal foods rather than powders (eTable 7 in Supplement 1). The certainty of evidence was downgraded for risk of bias. Absolute risk difference for a population with 20% withdrawal from the intervention was 258 cases per 1000 population (95% CI, 90-526 cases per 1000 population).

### Earlier Introduction of Egg

**Figure 2.**
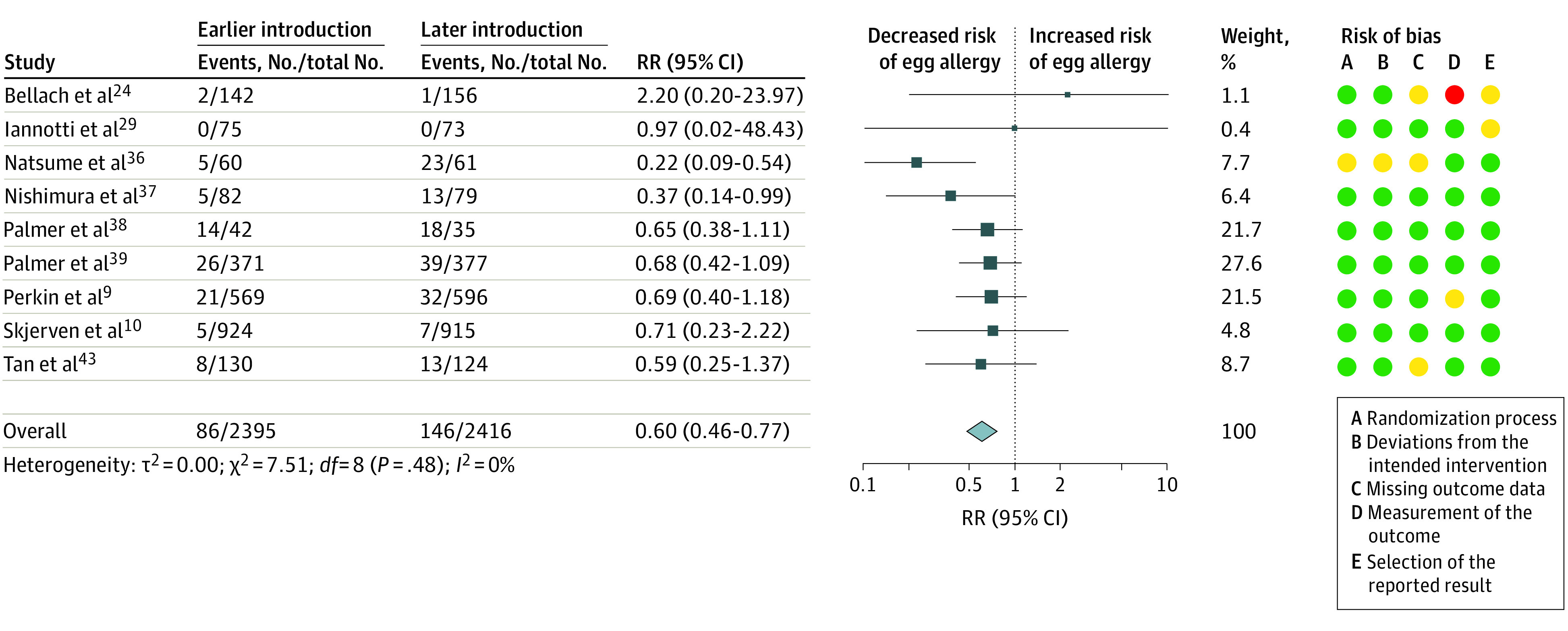
Earlier vs Later Introduction of Egg and Risk of Egg Allergy Squares indicate risk ratios (RRs), with horizontal lines indicating 95% CIs and size of squares indicating weight; diamond indicates the pooled estimate, with outer points of the diamond indicating the 95% CI. Green circles indicate low risk of bias; yellow, some concerns; and red, high risk of bias.

A meta-analysis of 9 trials^9,10,24,29,36,37,38,39,43^ (4811 participants) showed high-certainty evidence that earlier introduction of egg between 3 and 6 months of age was associated with decreased risk of egg allergy (RR, 0.60; 95% CI, 0.46-0.77; *I*^2^ = 0%) (Figure 2). Absolute risk difference for a population with 4% incidence of egg allergy was −16 cases per 1000 population (95% CI, −22 to −9 cases per 1000 population). Trial sequential analysis showed that the heterogeneity-adjusted optimal information size for detection of a 30% risk reduction had been reached (eFigure 2 in Supplement 1). There was no evidence for a difference in outcome between trials of egg only (6 trials^24,29,36,38,39,43^ [1646 participants]) and multiple allergenic foods including egg (3 trials^9,10,37^ [3165 participants]) (eTable 8 in Supplement 1). Subgroup analysis found a significant interaction related to dose of egg, with evidence for a greater reduction in risk of egg allergy in the low-dose group (*P* = .02 for interaction) (eTable 8 in Supplement 1). There was moderate-certainty evidence that earlier introduction of egg was associated with decreased risk of egg sensitization (8 trials^9,10,24,33,37,38,39,43^ [4325 participants]; RR, 0.81; 95% CI, 0.69-0.96; *I*^2^ = 18%) (eFigure 3 in Supplement 1). There was low-certainty evidence that earlier introduction of egg was associated with increased risk of withdrawal (13 trials^9,10,24,28,29,33,36,37,38,39,40,42,43^ [7442 participants]; RR, 1.58; 95% CI, 1.12-2.22; *I*^2^ = 90%) (eFigure 4 in Supplement 1). There was an asymmetrical funnel plot (eFigure 5 in Supplement 1), with increased withdrawal in larger studies. Certainty of evidence was downgraded for inconsistency and imprecision. There was very low-certainty evidence about the association of earlier egg introduction with risk of allergy^9,10,37,40^ or sensitization^9,10,37^ to any food, as almost all information for these analyses was derived from trials of multiple food interventions (eFigure 6 in Supplement 1).

### Earlier Introduction of Peanut

**Figure 3.**
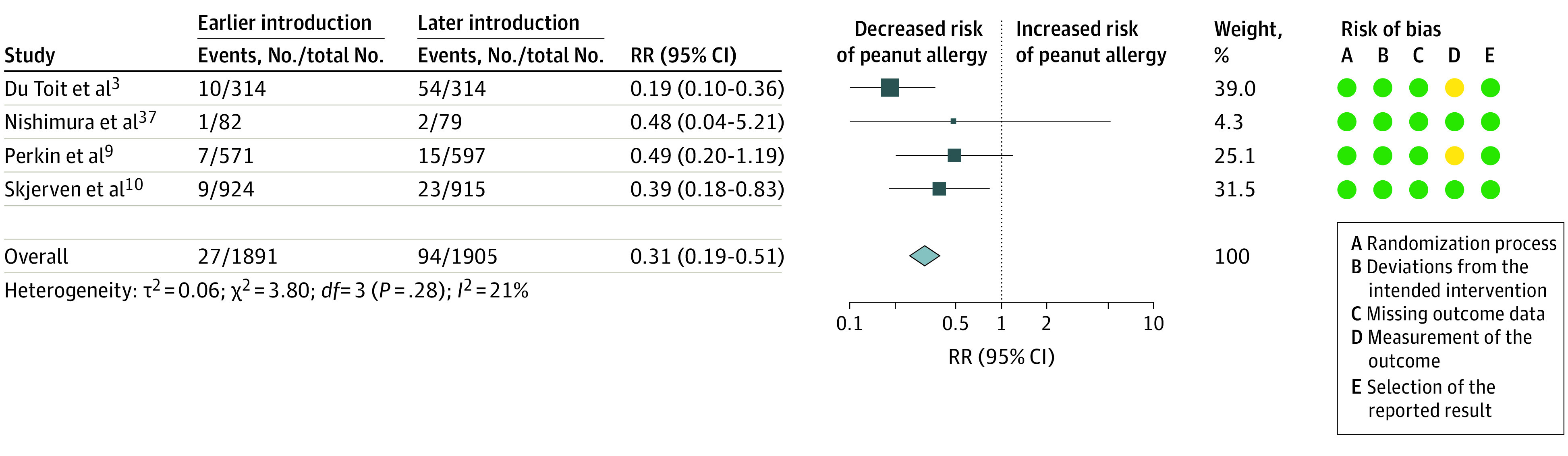
Earlier vs Later Introduction of Peanut and Risk of Peanut Allergy Squares indicate risk ratios (RRs), with horizontal lines indicating 95% CIs and size of squares indicating weight; diamond indicates the pooled estimate, with outer points of the diamond indicating the 95% CI. Green circles indicate low risk of bias; and yellow, some concerns.

Meta-analysis of 4 trials^3,9,10,37^ (3796 participants) showed high-certainty evidence that earlier introduction of peanut between 3 and 10 months of age was associated with decreased risk of peanut allergy (RR, 0.31; 95% CI, 0.19-0.51; *I*^2^ = 21%) (Figure 3). Absolute risk difference for a population with 2.5% incidence of peanut allergy was −17 cases per 1000 population (95% CI, −20 to −12 cases per 1000 population). Trial sequential analysis showed that the heterogeneity-adjusted optimal information size for detection of a 30% risk reduction had not been reached (eFigure 2 in Supplement 1). Most of the events contributing to this effect estimate were from a trial of single allergenic food introduction,^3^ in which participants in the control group were advised to avoid peanut until age 5 years, but trials of multiple allergenic food introduction without such prolonged avoidance advice in the control group^9,10,37^ also reported reduced peanut allergy (*P* = .06 for interaction) (eTable 9 in Supplement 1). There was low-certainty evidence that earlier introduction of peanut was associated with decreased risk of peanut sensitization (4 trials^4,9,10,37^ [3434 participants]; RR, 0.74; 95% CI, 0.46-1.20; *I*^2^ = 77%) (eFigure 3 in Supplement 1). Certainty of evidence was downgraded for inconsistency and imprecision, with only 1 study,^10^ which used an SPT, showing a stronger effect. There was very low-certainty evidence about earlier introduction of peanut and risk of withdrawal (6 trials^3,9,10,28,37,40^ [5343 participants]; RR, 1.91; 95% CI, 1.19-3.05; *I*^2^ = 91%) (eFigure 4 in Supplement 1); however, the single trial of peanut only^3^ showed no increase in withdrawal. Certainty of evidence was downgraded for risk of bias, inconsistency, and indirectness. There was also very low-certainty evidence about earlier introduction of peanut and decreased risk of allergy to any food (5 trials^4,9,10,37,40^ [3927 participants]; RR, 0.60; 95% CI, 0.38-0.94; *I*^2^ = 81%) (eFigure 6 in Supplement 1). Subgroup analysis found significant differences between multiple food intervention trials,^9,10,37,40^ which showed reduced allergy, and the single peanut-only intervention trial,^4^ which showed no effect (*P* = .02 for interaction) (eTable 9 in Supplement 1). There was low-certainty evidence for earlier introduction of peanut and risk of any food sensitization (4 trials^4,9,10,37^; 3456 participants; RR, 0.86; 95% CI, 0.71-1.05; *I*^2^ = 61%) (eFigure 3 in Supplement 1). Evidence was downgraded for inconsistency and imprecision.

### Earlier Introduction of Cow’s Milk

**Figure 4.**
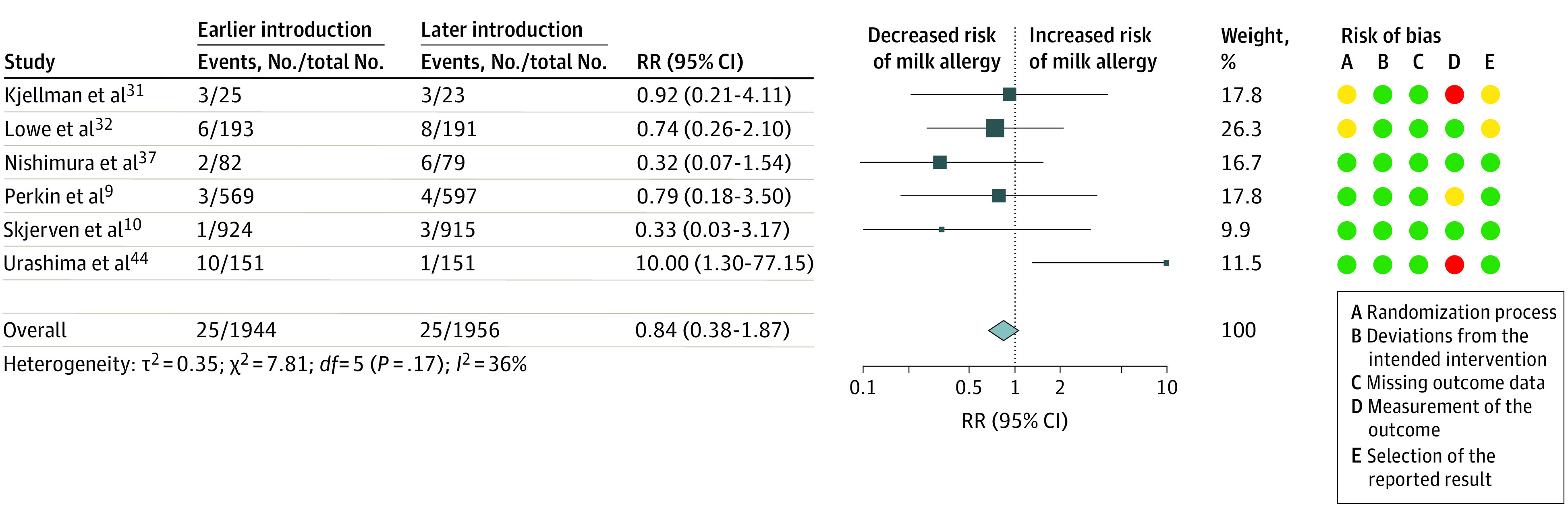
Earlier vs Later Introduction of Cow’s Milk and Risk of Cow’s Milk Allergy Squares indicate risk ratios (RRs), with horizontal lines indicating 95% CIs and size of squares indicating weight; diamond indicates the pooled estimate, with outer points of the diamond indicating the 95% CI. Green circles indicate low risk of bias; yellow, some concerns; and red, high risk of bias.

For the comparison between earlier and later introduction of cow’s milk, there was low- to very low-certainty evidence for all outcomes. Evidence was downgraded for indirectness because interventions were not representative of typical ways to introduce cow’s milk products to an infant diet and for imprecision. Meta-analysis of 6 trials^9,10,31,32,37,44^ (3900 participants) showed very low-certainty evidence about earlier introduction of milk between the first day of life and age 4 months and risk of milk allergy (RR, 0.84; 95% CI, 0.38-1.87; *I*^2^ = 36%) (Figure 4 and eTable 10 in Supplement 1). Most information was from studies with high risk of bias, with lack of blinding of outcome assessment as the main limitation. Sensitivity analyses restricted to low-risk-of-bias data reduced heterogeneity (2 trials^10,37^ [2000 participants]; RR, 0.32; 95% CI, 0.09-1.18; *I*^2^ = 0%) (eTable 11 in Supplement 1). There was very low-certainty evidence about earlier introduction of milk and risk of milk sensitization (7 trials^9,10,26,31,32,37,44^ [4887 participants]; RR, 1.14; 95% CI, 0.82-1.59; *I*^2^, 45%) (eFigure 3 in Supplement 1). Heterogeneity appeared to be partly explained by the method of outcome measurement, with studies using SPTs showing no heterogeneity in an exploratory, post hoc subgroup analysis (SPT: 3 trials^9,10,32^ [2974 participants]; RR, 0.64; 95% CI, 0.32-1.29; *I*^2^ = 0%; specific IgE: 4 trials^26,31,37,44^ [1913 participants]; RR, 1.29; 95% CI, 0.89-1.89; *I*^2^ = 62%; *P* = .08 for interaction). There was low-certainty evidence for earlier introduction of milk and risk of withdrawal (11 trials^9,10,25,26,28,31,32,37,40,41,44^ [7895 participants]; RR, 1.05; 95% CI, 0.61-1.82; *I*^2^ = 94%) (eFigure 4 in Supplement 1). There was an asymmetrical funnel plot (eFigure 5 in Supplement 1), with increased withdrawal in larger studies. Heterogeneity was explained by high rates of withdrawal in 2 pragmatic trials of multiple, high-dose interventions^9,10^ and a trial in which many participants withdrew from the delayed cow’s milk (soya formula) intervention^25^ due to a preference for earlier cow’s milk formula. There was no statistical heterogeneity with these 3 excluded trials^9,10,25^ (8 trials^26,28,31,32,37,40,41,44^ [3816 participants]; RR, 0.86; 95% CI, 0.70-1.06; *I*^2^ = 0%). There was very low-certainty evidence about earlier introduction of milk and risk of allergy to any food (6 trials^9,10,32,37,40,44^ [3981 participants]; RR, 0.67; 95% CI, 0.39-1.13; *I*^2^ = 83%) (eFigure 6 in Supplement 1). There was no information available about the effect of milk introduction without other allergenic foods on allergic sensitization to any food.

### Earlier Introduction of Other Foods

Evidence for the timing of introduction of other allergenic foods and risk of food allergy was limited (eTable 12 in Supplement 1). Wheat (5 trials^9,10,28,37,40^ [4658 participants]), soya (6 trials^25,28,31,32,37,41^ [2215 participants]), fish (3 trials^9,28,40^ [2098 participants]), and crustaceans and tree nuts (2 trials^28,40^ [795 participants]) were introduced earlier, usually as part of a multiple allergenic food introduction. Where meta-analysis was possible, only small numbers of participants with allergy to the relevant foods were included, and no evidence for a difference between groups was seen for earlier wheat introduction and wheat allergy (3 trials^9,10,37^ [3169 participants]; RR, 0.66; 95% CI, 0.10-4.47; *I*^2^ = 2%) or wheat sensitization (3 trials^9,10,37^ [2818 participants]; RR, 0.62; 95% CI, 0.29-1.34; *I*^2^ = 59%) or for earlier soya introduction and soya sensitization (2 trials^31,37^ [192 participants]; RR, 1.14; 95% CI, 0.79-1.65; *I*^2^ = 0%) (eTable 12 in Supplement 1).

## Discussion

This systematic review and meta-analysis found that earlier introduction of multiple allergenic foods was associated with reduced IgE-mediated allergy to any food; that earlier egg and peanut introduction were associated with lower risk of egg and peanut allergy, respectively; and that multiple food interventions can be difficult to adhere to. The findings support the concept of using earlier allergenic food introduction to prevent food allergy but highlight the need for more acceptable forms of multiple allergenic foods. Notably, most complementary feeding interventions were initiated before 6 months of age, which is against World Health Organization infant feeding guidance.^45^

These findings are consistent with those of the Preventing Atopic Dermatitis and Allergies in Children (PreventADALL) trial,^10^ which is, to our knowledge, the largest trial of multiple earlier allergenic food introduction. Previous clinical trials found evidence that earlier introduction of egg and peanut reduces risk of egg and peanut allergy, respectively, but to our knowledge, no trials have been able to confirm whether any food allergy can be prevented in this way.^2,3,4,9^ The Learning Early About Peanut Allergy (LEAP) trial^3^ of earlier peanut introduction found almost identical rates of sensitization and allergy to any food between the intervention and control groups. This lack of effect on overall food allergy risk in the LEAP trial contrasts with a marked reduction in peanut allergy.^3,4^ For infants and their caregivers, prevention of any food allergy is likely to be a more important goal than prevention of allergy to a single food. The data we report herein and those of the PreventADALL trial^10^ support the concept that earlier introduction of multiple allergenic foods can affect an infant’s risk for developing food allergy, which is likely to be an important outcome for families. The findings of this study suggest that approximately 38 families would need to be advised to introduce multiple allergenic foods earlier to their infant’s diet to prevent 1 infant from developing food allergy, although this number is reduced in families at higher risk. It is therefore important to establish safety, cost-effectiveness, and acceptability of earlier multiple allergenic food introduction.

Safety data from earlier allergenic food introduction trials^9,38^ were generally reassuring, although systemic allergic reactions can occur in infants at higher risk or those who already have a food allergy. However, we found in this systematic review and meta-analysis that earlier allergenic food introduction was associated with high rates of withdrawal from the intervention in the 2 large, pragmatic multiple allergenic food introduction trials.^9,10^ Only 29%^10^ and 34%^9^ of participants in the earlier introduction groups of these trials were able to fully adhere to the intervention. In 1 trial,^46^ nonadherence was associated with reported feeding difficulties and was more common with parent-reported allergy symptoms, belonging to a racial or ethnic minority group, increased maternal age, and lower maternal quality of life. In contrast to these larger studies, 3 smaller studies^28,37,40^ of multiple allergenic food introduction had lower rates of withdrawal, with similar rates in the earlier and later introduction groups. These smaller studies used multiple allergenic food protein powders rather than a stepwise introduction of normal foods. There is, however, widespread concern about the nutritional composition, texture, flavor, and marketing of commercial foods for infants,^47^ and these wider issues need to be considered before making recommendations to use commercial multiple allergenic food protein powders for food allergy prevention. One trial^9^ reported that earlier multiple allergenic food introduction did not have any adverse effect on breastfeeding rates. There was also no detectable adverse effect on infant growth, gastrointestinal or respiratory health, or development in this trial’s high-income setting.^48^ Most of the world’s infants are born in low- or middle-income countries, where food allergy is uncommon. The effect of earlier allergenic food introduction on general child health and development is unclear, and food allergy interventions that shorten exclusive breastfeeding duration in low- or middle-income settings may be harmful.^49^ Another important uncertainty that remains is whether earlier introduction of cow’s milk affects risk of cow’s milk allergy. Trials of earlier cow’s milk introduction identified few cases of milk allergy,^9,10,31,32,37,44^ and some had a high risk of bias.^50^

### Limitations

This study has limitations. It did not include formal individual patient data (IPD) analysis, although some trial IPD were in the public domain and we extracted those from publicly available data sets. A recent IPD meta-analysis^51^ of 2 studies suggested similar effectiveness of earlier peanut introduction in infants with and without eczema. Further IPD analysis may be helpful for better understanding adverse effect data and subgroup analyses but is unlikely to have a significant effect on the efficacy outcomes.^52^ Our data represent clinical trial populations and may therefore lack generalizability. In Australia, population-based studies^7,8^ demonstrated a shift to earlier peanut and egg introduction since 2016 but no reduction in peanut allergy associated with this shift. Population-based data on the effect of earlier multiple allergenic food introduction on the risk of any food allergy are not yet available.

## Conclusions

In this systematic review and meta-analysis, earlier introduction of multiple allergenic foods was associated with reduced IgE-mediated allergy to any food but high rates of withdrawal from the intervention. Further work is needed to develop allergenic food interventions that are safe and acceptable for infants and their families.

## Notes

Supplement 1.
eMethods
**eFigure 1.** PRISMA Flow Diagram of Study Selection **eFigure 2.** Post Hoc Trial Sequential Analysis for Key Study Findings **eFigure 3.** Earlier introduction of Allergenic Foods and Allergic Sensitization **eFigure 4.** Earlier Introduction of Allergenic Foods and Withdrawal From Study Intervention **eFigure 5.** Exploration of Small Study Effects Using Funnel Plots **eFigure 6.** Earlier Introduction of Allergenic Foods and Risk of Allergy to Any Food **eTable 1.** Characteristics of Included Studies **eTable 2.** Characteristics of Ongoing Studies **eTable 3.** Summary of Findings for Earlier Introduction of Multiple Allergenic Foods **eTable 4.** Summary of Findings for Earlier Introduction of Egg **eTable 5.** Summary of Findings for Earlier Introduction of Peanut **eTable 6.** Summary of Findings for Earlier Introduction of Cow’s Milk **eTable 7.** Subgroup Analyses for Earlier Introduction of Multiple Allergenic Foods **eTable 8.** Subgroup Analyses for Earlier Introduction of Egg **eTable 9.** Subgroup Analyses for Earlier Introduction of Peanut **eTable 10.** Subgroup Analyses for Earlier Introduction of Cow’s Milk **eTable 11.** Sensitivity Analysis of Low Risk of Bias Data **eTable 12.** Earlier Introduction of Other Common Allergenic Foods
eReferences
**eAppendix.** Search Strategies

Supplement 2.
Data Sharing Statement

## References

[1] Cafarotti A, Giovannini M, Begin P, Brough HA, Arasi S. Management of IgE-mediated food allergy in the 21st century. Clin Exp Allergy. 2023;53(1):25-38. doi:10.1111/cea.14241 36200952 PMC10092460

[2] Ierodiakonou D, Garcia-Larsen V, Logan A, . Timing of allergenic food introduction to the infant diet and risk of allergic or autoimmune disease: a systematic review and meta-analysis. JAMA. 2016;316(11):1181-1192. doi:10.1001/jama.2016.12623 27654604

[3] Du Toit G, Roberts G, Sayre PH, ; LEAP Study Team. Randomized trial of peanut consumption in infants at risk for peanut allergy. N Engl J Med. 2015;372(9):803-813. doi:10.1056/NEJMoa1414850 25705822 PMC4416404

[4] Du Toit G, Sayre PH, Roberts G, ; Immune Tolerance Network Learning Early About Peanut Allergy study team. Allergen specificity of early peanut consumption and effect on development of allergic disease in the Learning Early About Peanut Allergy study cohort. J Allergy Clin Immunol. 2018;141(4):1343-1353. doi:10.1016/j.jaci.2017.09.034 29097103 PMC5889963

[5] Halken S, Muraro A, de Silva D, ; European Academy of Allergy and Clinical Immunology Food Allergy and Anaphylaxis Guidelines Group. EAACI guideline: preventing the development of food allergy in infants and young children (2020 update). Pediatr Allergy Immunol. 2021;32(5):843-858. doi:10.1111/pai.13496 33710678

[6] Fleischer DM, Chan ES, Venter C, . A consensus approach to the primary prevention of food allergy through nutrition: guidance from the American Academy of Allergy, Asthma, and Immunology; American College of Allergy, Asthma, and Immunology; and the Canadian Society for Allergy and Clinical Immunology. J Allergy Clin Immunol Pract. 2021;9(1):22-43.e4. doi:10.1016/j.jaip.2020.11.002 33250376

[7] Soriano VX, Peters RL, Ponsonby AL, . Earlier ingestion of peanut after changes to infant feeding guidelines: the EarlyNuts study. J Allergy Clin Immunol. 2019;144(5):1327-1335.e5. doi:10.1016/j.jaci.2019.07.032 31401287

[8] Soriano VX, Peters RL, Moreno-Betancur M, . Association between earlier introduction of peanut and prevalence of peanut allergy in infants in Australia. JAMA. 2022;328(1):48-56. doi:10.1001/jama.2022.9224 35788795 PMC9257582

[9] Perkin MR, Logan K, Tseng A, ; EAT Study Team. Randomized trial of introduction of allergenic foods in breast-fed infants. N Engl J Med. 2016;374(18):1733-1743. doi:10.1056/NEJMoa1514210 26943128

[10] Skjerven HO, Lie A, Vettukattil R, . Early food intervention and skin emollients to prevent food allergy in young children (PreventADALL): a factorial, multicentre, cluster-randomised trial. Lancet. 2022;399(10344):2398-2411. doi:10.1016/S0140-6736(22)00687-0 35753340

[11] Boyle R, Garcia-Larsen V, Leonardi-Bee J, Perkin M, Ierodiakonou D, Kimkool P. Systematic review and meta-analysis of timing of allergenic food introduction and risk of IgE-mediated food allergy. PROSPERO 2013 CRD42013004239. Accessed February 13, 2023. https://www.crd.york.ac.uk/prospero/display_record.php?ID=CRD42013004239 10.1001/jamapediatrics.2023.0142 PMC10043805 36972063

[12] Page MJ, McKenzie JE, Bossuyt PM, . The PRISMA 2020 statement: an updated guideline for reporting systematic reviews. BMJ. 2021;372(71):n71. doi:10.1136/bmj.n71 33782057 PMC8005924

[13] Page MJ, Moher D, Bossuyt PM, . PRISMA 2020 explanation and elaboration: updated guidance and exemplars for reporting systematic reviews. BMJ. 2021;372(160):n160. doi:10.1136/bmj.n160 33781993 PMC8005925

[14] Food Allergen Labeling and Consumer Protection Act of 2004, US Public Law 108-282, 108th Cong (2004).

[15] Sterne JAC, Savović J, Page MJ, . RoB 2: a revised tool for assessing risk of bias in randomised trials. BMJ. 2019;366:l4898. doi:10.1136/bmj.l4898 31462531

[16] Egger M, Davey Smith G, Schneider M, Minder C. Bias in meta-analysis detected by a simple, graphical test. BMJ. 1997;315(7109):629-634. doi:10.1136/bmj.315.7109.629 9310563 PMC2127453

[17] R Foundation for Statistical Computing. (2022). R: A language and environment for statistical computing. Accessed February 13, 2023. https://www.R-project.org/

[18] Balduzzi S, Rücker G, Schwarzer G. How to perform a meta-analysis with R: a practical tutorial. Evid Based Ment Health. 2019;22(4):153-160. doi:10.1136/ebmental-2019-300117 31563865 PMC10231495

[19] Viechtbauer W. Conducting meta-analyses in R with the metafor package. J Stat Softw. 2010;36(3):1-48. doi:10.18637/jss.v036.i03

[20] DerSimonian R, Laird N. Meta-analysis in clinical trials. Control Clin Trials. 1986;7(3):177-188. doi:10.1016/0197-2456(86)90046-2 3802833

[21] *Trial Sequential Analysis (TSA)*. Version 0.9.5.10 Beta. The Copenhagen Trial Unit, Centre for Clinical Intervention Research; 2021.

[22] Guyatt GH, Oxman AD, Vist GE, ; GRADE Working Group. GRADE: an emerging consensus on rating quality of evidence and strength of recommendations. BMJ. 2008;336(7650):924-926. doi:10.1136/bmj.39489.470347.AD 18436948 PMC2335261

[23] Guyatt G, Oxman AD, Akl EA, . GRADE guidelines: 1. Introduction—GRADE evidence profiles and summary of findings tables. J Clin Epidemiol. 2011;64(4):383-394. doi:10.1016/j.jclinepi.2010.04.026 21195583

[24] Bellach J, Schwarz V, Ahrens B, . Randomized placebo-controlled trial of hen’s egg consumption for primary prevention in infants. J Allergy Clin Immunol. 2017;139(5):1591-1599.e2. doi:10.1016/j.jaci.2016.06.045 27523961

[25] Brown EB, Josephson BM, Levine HS, Rosen M. A prospective study of allergy in a pediatric population. The role of heredity in the incidence of allergies, and experience with milk-free diet in the newborn. AJDC. 1969;117(6):693-698. 5818902

[26] de Jong MH, Scharp-van der Linden VT, Aalberse RC, Oosting J, Tijssen JG, de Groot CJ. Randomised controlled trial of brief neonatal exposure to cows’ milk on the development of atopy. Arch Dis Child. 1998;79(2):126-130. doi:10.1136/adc.79.2.126 9797592 PMC1717657

[27] Halpern SR, Sellars WA, Johnson RB, Anderson DW, Saperstein S, Reisch JS. Development of childhood allergy in infants fed breast, soy, or cow milk. J Allergy Clin Immunol. 1973;51(3):139-151. doi:10.1016/0091-6749(73)90019-5 4739434

[28] Holl JL, Bilaver LA, Finn DJ, Savio K. A randomized trial of the acceptability of a daily multi-allergen food supplement for infants. Pediatr Allergy Immunol. 2020;31(4):418-420. doi:10.1111/pai.13223 32030829 PMC7318591

[29] Iannotti LL, Lutter CK, Stewart CP, . Eggs in early complementary feeding and child growth: a randomized controlled trial. Pediatrics. 2017;140(1):e20163459. doi:10.1542/peds.2016-3459 28588101

[30] Johnstone DE, Dutton AM. Dietary prophylaxis of allergic disease in children. N Engl J Med. 1966;274(13):715-719. doi:10.1056/NEJM196603312741305 5952173

[31] Kjellman NI, Johansson SG. Soy versus cow’s milk in infants with a biparental history of atopic disease: development of atopic disease and immunoglobulins from birth to 4 years of age. Clin Allergy. 1979;9(4):347-358. doi:10.1111/j.1365-2222.1979.tb02493.x 573185

[32] Lowe AJ, Hosking CS, Bennett CM, . Effect of a partially hydrolyzed whey infant formula at weaning on risk of allergic disease in high-risk children: a randomized controlled trial. J Allergy Clin Immunol. 2011;128(2):360-365.e4. doi:10.1016/j.jaci.2010.05.006 21696814

[33] Makrides M, Hawkes JS, Neumann MA, Gibson RA. Nutritional effect of including egg yolk in the weaning diet of breast-fed and formula-fed infants: a randomized controlled trial. Am J Clin Nutr. 2002;75(6):1084-1092. doi:10.1093/ajcn/75.6.1084 12036817

[34] Miskelly FG, Burr ML, Vaughan-Williams E, Fehily AM, Butland BK, Merrett TG. Infant feeding and allergy. Arch Dis Child. 1988;63(4):388-393. doi:10.1136/adc.63.4.388 3365008 PMC1778829

[35] Hand S, Dunstan F, Jones K, Doull I. The effect of diet in infancy on asthma in young adults: the Merthyr Allergy Prevention Study. Thorax. 2021;76(11):1072-1077. doi:10.1136/thoraxjnl-2020-215040 33963089

[36] Natsume O, Kabashima S, Nakazato J, ; PETIT Study Team. Two-step egg introduction for prevention of egg allergy in high-risk infants with eczema (PETIT): a randomised, double-blind, placebo-controlled trial. Lancet. 2017;389(10066):276-286. doi:10.1016/S0140-6736(16)31418-0 27939035

[37] Nishimura T, Fukazawa M, Fukuoka K, . Early introduction of very small amounts of multiple foods to infants: a randomized trial. Allergol Int. 2022;71(3):345-353. doi:10.1016/j.alit.2022.03.001 35367136

[38] Palmer DJ, Metcalfe J, Makrides M, . Early regular egg exposure in infants with eczema: a randomized controlled trial. J Allergy Clin Immunol. 2013;132(2):387-92.e1. doi:10.1016/j.jaci.2013.05.002 23810152

[39] Palmer DJ, Sullivan TR, Gold MS, Prescott SL, Makrides M. Randomized controlled trial of early regular egg intake to prevent egg allergy. J Allergy Clin Immunol. 2017;139(5):1600-1607.e2. doi:10.1016/j.jaci.2016.06.052 27554812

[40] Quake AZ, Liu TA, D’Souza R, . Early introduction of multi-allergen mixture for prevention of food allergy: pilot study. Nutrients. 2022;14(4):737. doi:10.3390/nu14040737 35215387 PMC8879339

[41] Sakihara T, Otsuji K, Arakaki Y, Hamada K, Sugiura S, Ito K. Randomized trial of early infant formula introduction to prevent cow’s milk allergy. J Allergy Clin Immunol. 2021;147(1):224-232.e8. doi:10.1016/j.jaci.2020.08.021 32890574

[42] Stewart CP, Caswell B, Iannotti L, . The effect of eggs on early child growth in rural Malawi: the Mazira Project randomized controlled trial. Am J Clin Nutr. 2019;110(4):1026-1033. doi:10.1093/ajcn/nqz163 31386106 PMC6766435

[43] Wei-Liang Tan J, Valerio C, Barnes EH, ; Beating Egg Allergy Trial (BEAT) Study Group. A randomized trial of egg introduction from 4 months of age in infants at risk for egg allergy. J Allergy Clin Immunol. 2017;139(5):1621-1628.e8. doi:10.1016/j.jaci.2016.08.035 27742394

[44] Urashima M, Mezawa H, Okuyama M, . Primary prevention of cow’s milk sensitization and food allergy by avoiding supplementation with cow’s milk formula at birth: a randomized clinical trial. JAMA Pediatr. 2019;173(12):1137-1145. doi:10.1001/jamapediatrics.2019.3544 31633778 PMC6806425

[45] World Health Organization. Complementary feeding. Accessed February 14, 2023. https://www.who.int/health-topics/complementary-feeding#tab=tab_1

[46] Perkin MR, Bahnson HT, Logan K, ; Enquiring About Tolerance (EAT) study team. Factors influencing adherence in a trial of early introduction of allergenic food. J Allergy Clin Immunol. 2019;144(6):1595-1605. doi:10.1016/j.jaci.2019.06.046 31812183 PMC6904906

[47] *Guidance on Ending the Inappropriate Promotion of Foods for Infants and Young Children: Implementation Manual*. World Health Organization; 2017. Accessed February 13, 2023. https://apps.who.int/iris/bitstream/handle/10665/260137/9789241513470-eng.pdf?sequence=1

[48] Perkin MR, Bahnson HT, Logan K, . Association of early introduction of solids with infant sleep: a secondary analysis of a randomized clinical trial. JAMA Pediatr. 2018;172(8):e180739. doi:10.1001/jamapediatrics.2018.0739 29987321 PMC6142923

[49] Turner PJ, Campbell DE, Boyle RJ, Levin ME. Primary prevention of food allergy: translating evidence from clinical trials to population-based recommendations. J Allergy Clin Immunol Pract. 2018;6(2):367-375. doi:10.1016/j.jaip.2017.12.015 29524992 PMC5840515

[50] Helfer B, Leonardi-Bee J, Mundell A, . Conduct and reporting of formula milk trials: systematic review. BMJ. 2021;375(2202):n2202. doi:10.1136/bmj.n2202 34645600 PMC8513520

[51] Logan K, Bahnson HT, Ylescupidez A, . Early introduction of peanut reduces peanut allergy across risk groups in pooled and causal inference analyses. Allergy. Published online November 27, 2022. doi:10.1111/all.15597 36435990 PMC10202125

[52] Van Vogt E, Cro S, Cornelius VR, et al. Individual participant data meta-analysis versus aggregate data meta-analysis: a case study in eczema and food allergy prevention. Clin Exp Allergy. 2021;52(5):628-645. doi:10.1111/cea.14085 PMC9302682 34939249

