# Supplementary material for: Timing of Allergenic Food Introduction and Risk of Immunoglobulin E–Mediated Food Allergy: A Systematic Review and Meta-analysis
Source: JAMA Pediatr. 2023 Mar 27;177(5):489–97. doi: 10.1001/jamapediatrics.2023.0142 (PMC10043805; doi:10.1001/jamapediatrics.2023.0142)
Supplement: Supplement 1. — eMethods eFigure 1. PRISMA Flow Diagram of Study Selection eFigure 2. Post Hoc Trial Sequential Analysis for Key Study Findings eFigure 3. Earlier introduction of Allergenic Foods and Allergic Sensitization eFigure 4. Earlier Introduction of Allergenic Foods and Withdrawal From Study Intervention eFigure 5. Exploration of Small Study Effects Using Funnel Plots eFigure 6. Earlier Introduction of Allergenic Foods and Risk of Allergy to Any Food eTable 1. Characteristics of Included Studies eTable 2. Characteristics of Ongoing Studies eTable 3. Summary of Findings for Earlier Introduction of Multiple Allergenic Foods eTable 4. Summary of Findings for Earlier Introduction of Egg eTable 5. Summary of Findings for Earlier Introduction of Peanut eTable 6. Summary of Findings for Earlier Introduction of Cow’s Milk eTable 7. Subgroup Analyses for Earlier Introduction of Multiple Allergenic Foods eTable 8. Subgroup Analyses for Earlier Introduction of Egg eTable 9. Subgroup Analyses for Earlier Introduction of Peanut eTable 10. Subgroup Analyses for Earlier Introduction of Cow’s Milk eTable 11. Sensitivity Analysis of Low Risk of Bias Data eTable 12. Earlier Introduction of Other Common Allergenic Foods eReferences eAppendix. Search Strategies [file jamapediatr-e230142-s001.pdf]

# Supplementary Online Content

Scarpone R, Kimkool P, Ierodiakonou D, et al. Timing of allergenic food introduction and risk of immunoglobulin E–mediated food allergy: a systematic review and meta-analysis. *JAMA Pediatr*. Published online March 27, 2023. doi:10.1001/jamapediatrics.2023.0142

**eMethods**
**eFigure 1.** PRISMA Flow Diagram of Study Selection
**eFigure 2.** Post Hoc Trial Sequential Analysis for Key Study Findings
**eFigure 3.** Earlier introduction of Allergenic Foods and Allergic Sensitization
**eFigure 4.** Earlier Introduction of Allergenic Foods and Withdrawal From Study Intervention
**eFigure 5.** Exploration of Small Study Effects Using Funnel Plots
**eFigure 6.** Earlier Introduction of Allergenic Foods and Risk of Allergy to Any Food
**eTable 1.** Characteristics of Included Studies
**eTable 2.** Characteristics of Ongoing Studies
**eTable 3.** Summary of Findings for Earlier Introduction of Multiple Allergenic Foods
**eTable 4.** Summary of Findings for Earlier Introduction of Egg
**eTable 5.** Summary of Findings for Earlier Introduction of Peanut
**eTable 6.** Summary of Findings for Earlier Introduction of Cow's Milk
**eTable 7.** Subgroup Analyses for Earlier Introduction of Multiple Allergenic Foods
**eTable 8.** Subgroup Analyses for Earlier Introduction of Egg
**eTable 9.** Subgroup Analyses for Earlier Introduction of Peanut
**eTable 10.** Subgroup Analyses for Earlier Introduction of Cow's Milk
**eTable 11.** Sensitivity Analysis of Low Risk of Bias Data
**eTable 12.** Earlier Introduction of Other Common Allergenic Foods
**eReferences**
**eAppendix.** Search Strategies

This supplementary material has been provided by the authors to give readers additional information about their work.

© 2023 Scarpone R et al. *JAMA Pediatrics*.

## eMethods

This is a targeted update of a systematic review originally commissioned by the UK Food Standards Agency to inform revisions to mother and infant feeding guidance.[1] The previous version of this systematic review was part of a series evaluating the influence of maternal diet during pregnancy and lactation, and infant diet during the first year of life, on risk of allergic or autoimmune disease in the child at any time of life.[2,3] The previous systematic reviews and this targeted update review were all registered on the International Prospective Register of Systematic Reviews (PROSPERO references for original reviews CRD42013003802, CRD42013004239, CRD42013004252, registered on August 5, 2013; targeted update CRD42013004239, registered on May 28, 2021).

### Types of Study Included

We included only randomized controlled trials published from database inception until the search date (May 11, 2021; updated on June 28, 2022 and December 29, 2022). We did not include quasi- or non-randomized controlled trials, observational studies, non-comparative studies, or non-human studies.

### Population

Infants between the age of 0 and the end of the 12th postpartum month. Studies where subject eligibility was defined based on a family history of allergy were included, since this applies to a significant proportion of infants. We did not include trials undertaken in specific populations such as very premature infants, infants who all had a specific, uncommon health condition (e.g., affecting less than 1% of general populations), or other populations which were clearly unrepresentative of more general populations.

### Intervention

Timing of the introduction of common allergenic foods into the infant diet, namely cow's milk, egg, peanut, tree nuts, wheat, soya, crustaceans, and fish. We did not include trials which evaluated nutritional supplements such as vitamins, probiotics, prebiotics or synbiotics, trials of one mammalian milk versus an immunologically closely related mammalian milk (e.g., cow's milk versus sheep or goat milk), trials of multifaceted interventions where timing of allergenic food introduction to the infant diet was a minor component of the overall intervention (e.g., trials involving maternal dietary interventions and environmental control in addition to infant allergenic food introduction/avoidance), or trials of timing of solid food introduction without use of allergenic foods.

### Comparator

We included trials which used delayed allergenic food introduction, breastfeeding or human breastmilk, amino-acid formula, other low allergen exposure interventions, or usual care as the comparator. We did not include trials which compared an allergenic food with a partially or extensively hydrolyzed formula, whether or not that formula was derived from the same protein source as the allergenic food. We included studies which compared different doses, forms, or routes of an exposure e.g., high or low allergen intake, single or multiple allergenic food introduction.

### Study Outcomes

Allergy to any food was the primary efficacy outcome and withdrawal from study intervention was the primary safety outcome. Secondary outcomes included allergic sensitization to any or single foods, and allergy to specific foods. Age at assessment was the closest reported timepoint to age 3 years. Data outside the age range 1-5 years were extracted and reported, but not included in meta-analysis with 1-5 year data. Where there were multiple assessments, we included our preferred method as stated below, then the closest timepoint to age 3 years, then the outcome with the highest proportion of randomized participants evaluated.
Primary outcomes:
1. Food allergy to any food – defined by double-blind placebo-controlled food challenge, open food challenge, medical diagnosis or by self/parent report.
Preferred method of assessment: challenge-proven food allergy to any food, assessed at ~3 years. Where multiple measures were reported, cumulative incidence was analyzed preferentially.
2. Safety outcome – withdrawal from study intervention.
Preferred method of assessment: number of randomized participants in each group who were reported to have withdrawn from treatment or been lost to follow up during the study intervention period, for either unspecified reasons, or for any reason that could potentially be associated with the intervention.
Secondary outcomes:
1. Allergic sensitization to any food – defined as positive skin prick test and/or specific immunoglobulin E (IgE) test to at least one food allergen, using recognized methodologies and scoring criteria.

© 2023 Scarpone R et al. *JAMA Pediatrics*.

Preferred method of assessment: sensitization to at least one common food allergen, assessed at ~3 years. Where multiple measures were reported, point prevalence was analyzed preferentially.
2. Food allergy to a specific food – defined by double-blind placebo-controlled food challenge, by open food challenge, by medical diagnosis or by self/parent report.
Preferred method of assessment: challenge-proven food allergy to specific foods – cow's milk, egg, wheat, soya, peanut, tree nut, fish, or crustacean – assessed at ~3 years. Where multiple measures were reported, cumulative incidence was analyzed preferentially.
3. Allergic sensitization to a specific food – defined as positive skin prick test and/or specific IgE test to cow's milk, egg, wheat, soya, peanut, tree nut, fish, or crustacean, using recognized methodologies and scoring criteria.
Preferred method of assessment: sensitization to the relevant food allergen, assessed at ~3 years. Where multiple measures were reported, point prevalence was analyzed preferentially.

**Search Strategy**

The search strategies included both text terms and subject heading terms where appropriate. The search strategies were initially developed for use on the Medline database and then adapted for use on the other databases. We searched for eligible studies in Medline, Embase, Cochrane Library (CENTRAL) with no specified start date. The search was run on May 11, 2021, and included all studies published as either full text, letter, or abstract publications up to that date, and was updated on June 28, 2022 and December 29, 2022. We included peer reviewed publications, and abstract publications if they contained data that had not subsequently been published as a peer reviewed publication. We reviewed the bibliography of eligible studies for possible additional publications, and included all eligible publications, regardless of the language. Where necessary we contacted the authors of eligible or potentially eligible studies to request further details. The search strategies were extensively piloted and refined to optimize sensitivity, comparing search results with those of other high quality published systematic reviews. The final search strategies for this review as run in each database are shown in the Appendix and can also be accessed at
https://www.crd.york.ac.uk/PROSPEROFILES/4239_STRATEGY_20210527.pdf.

**Study Selection**

Titles, abstracts, and full texts of the identified studies were independently reviewed and assessed for eligibility against the inclusion criteria by two authors (R.S. and P.K.). Any discrepancies were resolved with the help of a third author (R.J.B.). Electronic records were kept regarding included and excluded studies for audit purposes. The reasons for the exclusion of any relevant studies were recorded, however ineligible studies were not analyzed further.

**Data Extraction**

An Excel data extraction form based on the form used in the previous systematic review was developed, piloted, and refined by three authors (R.S., P.K., and R.J.B.). Data extraction was undertaken by two authors (R.S. and P.K.). Disagreements and uncertainties were discussed in team meetings with a third author (R.J.B.). We extracted all relevant data from included studies, including data that could not be included in meta-analysis (e.g., which were not appropriately reported).

**Risk of Bias Assessment**

Review level bias: Publication bias was assessed using funnel plots and Egger's test where at least 10 trials were included in a meta-analysis.[4] Possible causes for asymmetry other than publication bias were also considered.
Study level bias: The risk of bias in included trials was assessed using the revised Cochrane risk-of-bias tool for randomized trials (RoB 2).[5] Overall risk of bias was considered low, some concerns, or high where the study outcome risk of bias was judged to be low for all domains of the RoB 2, some concerns for one domain, or high for at least one domain or some concerns for more than one, respectively.

**Strategy for Data Synthesis**

Meta-analysis was undertaken where ≥2 studies reported the same outcome for a given intervention. Our approach to meta-analysis was inclusive, however, data were explored for important sources of statistical or clinical heterogeneity.
Data extraction: Data were extracted using the number of events and total number of participants evaluated in each group. We did not plan formal individual patient data analysis but used data from publicly available datasets where necessary and where appropriate we contacted authors to clarify queries and include studies in meta-analysis.
Heterogeneity: Heterogeneity was quantified using $I^2$. Where extreme levels of heterogeneity were detected ($I^2$>75%), we performed sensitivity analyses to assess the effect of excluding outliers and re-considered whether

© 2023 Scarpone R et al. *JAMA Pediatrics*.

quantitative data synthesis was appropriate. Where possible, sensitivity/subgroup analyses were used to explore sources of heterogeneity arising from study characteristics.

Data analysis: Random effect meta-analyses were performed to allow for the anticipated heterogeneity between studies. Data were imported into R version 4.2.0 and analyzed using the “meta” version 5.2-0 and “metafor” version 3.4-0 packages.[6-8] The Mantel-Haenszel and DerSimonian and Laird methods were used.[9,10] Pooled results are presented as relative risks with 95% confidence intervals, and also expressed as risk differences where possible. P<0.05 was considered statistically significant. *Post hoc* trial sequential analysis (TSA) was used to quantify statistical reliability of the key positive review findings – for earlier multiple allergenic food introduction and any food allergy, earlier egg and egg allergy, and earlier peanut and peanut allergy.[11] A 2-sided P<0.05 significance level, 80% power, and control event rates pooled from the largest included studies (5% for any food allergy, 4% for egg allergy, and 2.5% for peanut allergy) were used to estimate optimal heterogeneity-adjusted information sizes needed to identify a relative risk reduction of 30%. TSA quantifies statistical reliability of data in a cumulative meta-analysis in a similar way to an interim analysis in a single randomized clinical trial. Statistical analyses were undertaken by three authors (R.S., D.I., and J.L.-B.).

Key findings are presented in Summary of Findings tables. Individual study results which were not appropriate for meta-analysis were reported narratively.

**Sensitivity and Subgroup Analyses**

Prespecified sensitivity analysis:

1. Low risk of bias – data evaluated as at low risk of bias were analyzed separately, excluding data with some concerns or high risk of bias.

Prespecified subgroup analyses:

1. Single versus multiple allergenic food introduction.
2. High versus low allergen intake, using a cut-off of 2g per week of the relevant individual food protein(s).
3. Milk feeding status at enrolment – exclusively breastmilk, with or without non-milk foods, versus mixed breastmilk/other milk or non-breastmilk fed.

Subgroup analyses were conducted using study-level variables. Additional sensitivity/subgroup analyses were undertaken to explore statistical heterogeneity if this was identified in the meta-analyses.

**Review Registration**

Details of the protocol for this systematic review were registered on PROSPERO on May 28, 2021, prior to title screening or selecting any studies from the search results and can be accessed at https://www.crd.york.ac.uk/PROSPERO/display_record.php?ID=CRD42013004239.

**Differences Between the Protocol and the Review**

One new author joined the review team (R.S.). We included TSA as an additional statistical measure of confidence in the key positive study findings.

**GRADE Evaluation of Evidence**

The certainty of evidence in this report was rated using the GRADE approach, which has four levels of certainty: high, moderate, low, and very low.[12,13] Evidence can be downgraded by one or two levels if there is serious or very serious risk of bias, inconsistency of results, indirectness of evidence, imprecision, or likely or very likely publication bias. The interpretation of GRADE certainty ratings is that for high certainty evidence further research is very unlikely to change our confidence in the estimate of effect; for moderate certainty evidence further research is likely to have an important impact on our confidence in the estimate of effect and may change the estimate; for low certainty evidence further research is very likely to have an important impact on our confidence in the estimate of effect and is likely to change the estimate; and for very low certainty evidence any estimate of effect is very uncertain.

© 2023 Scarpone R et al. *JAMA Pediatrics*.

## eFigure 1. PRISMA Flow Diagram of Study Selection

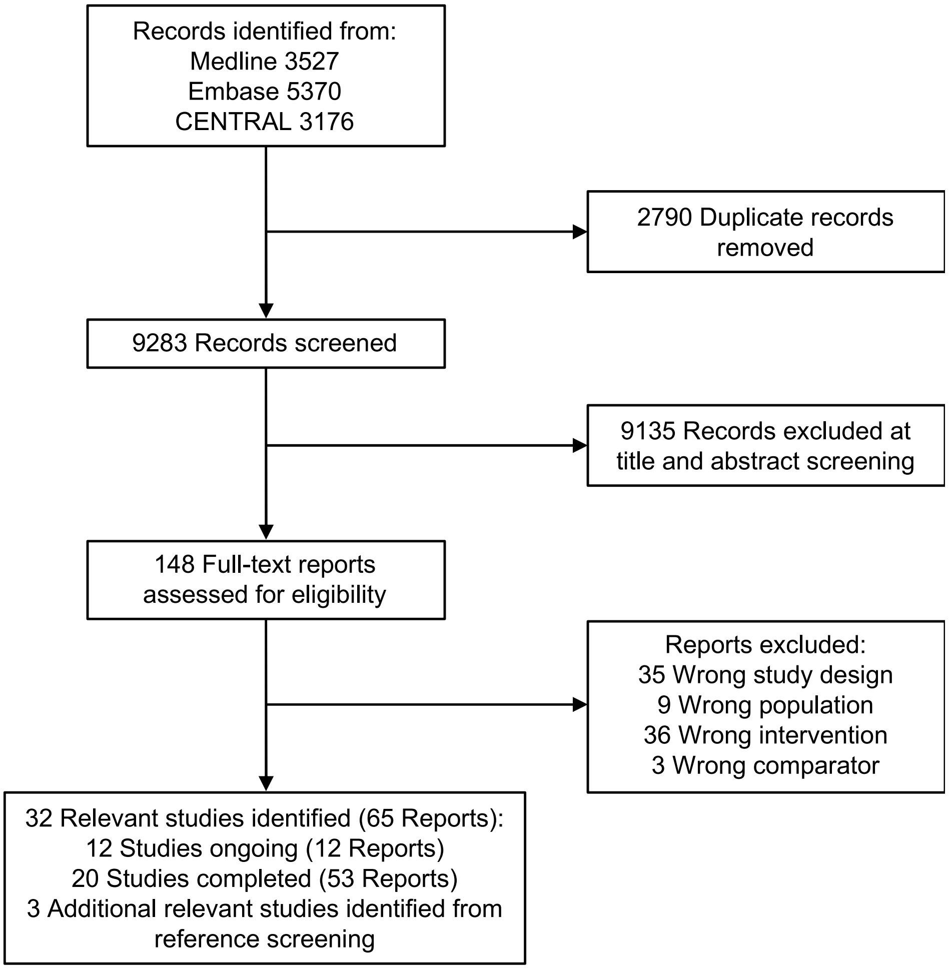


Search results and study selection procedure. Searches were run on May 11, 2021, and updated on June 28, 2022 and December 29, 2022.

© 2023 Scarpone R et al. *JAMA Pediatrics*.

**eFigure 2. Post Hoc Trial Sequential Analysis for Key Study Findings**

A

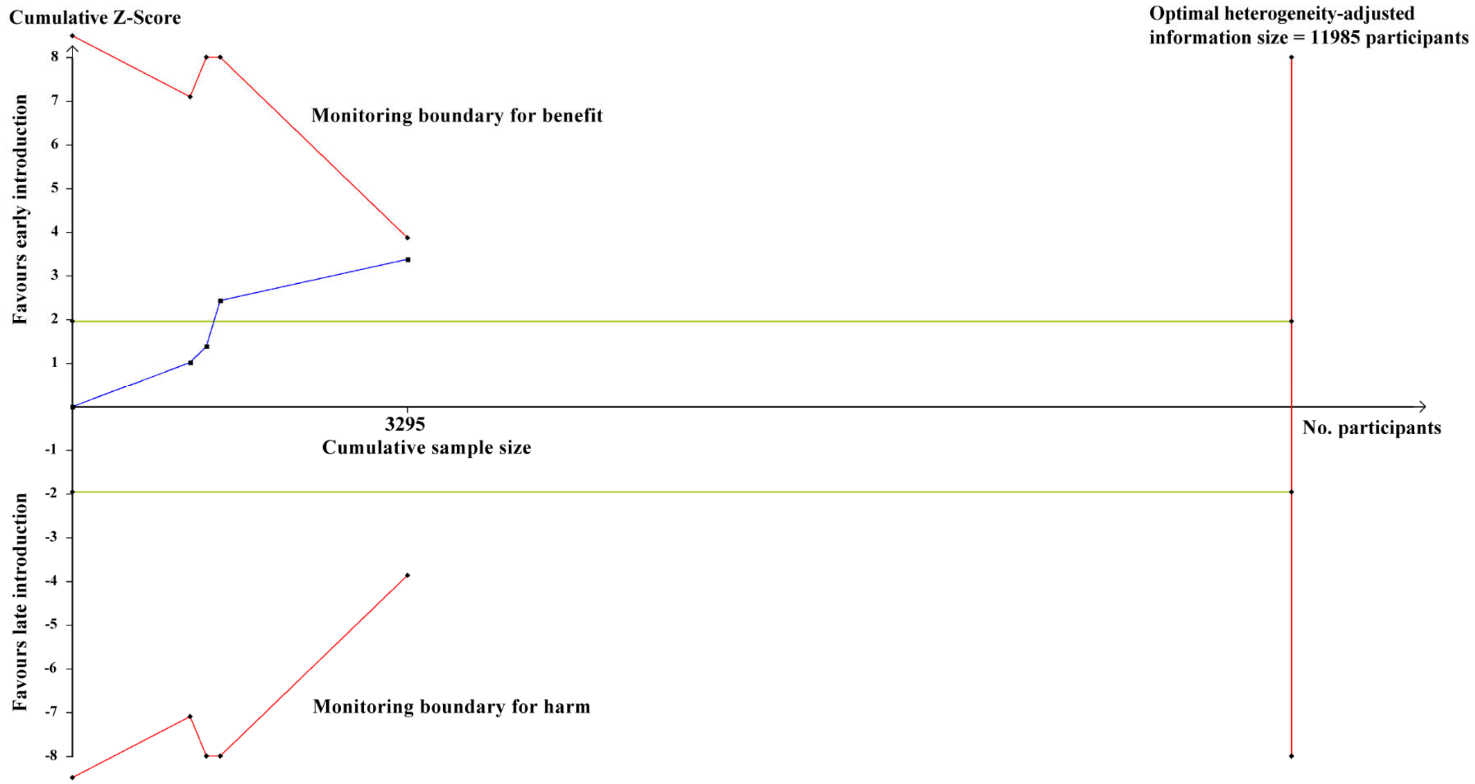


© 2023 Scarpone R et al. *JAMA Pediatrics*.

**B**

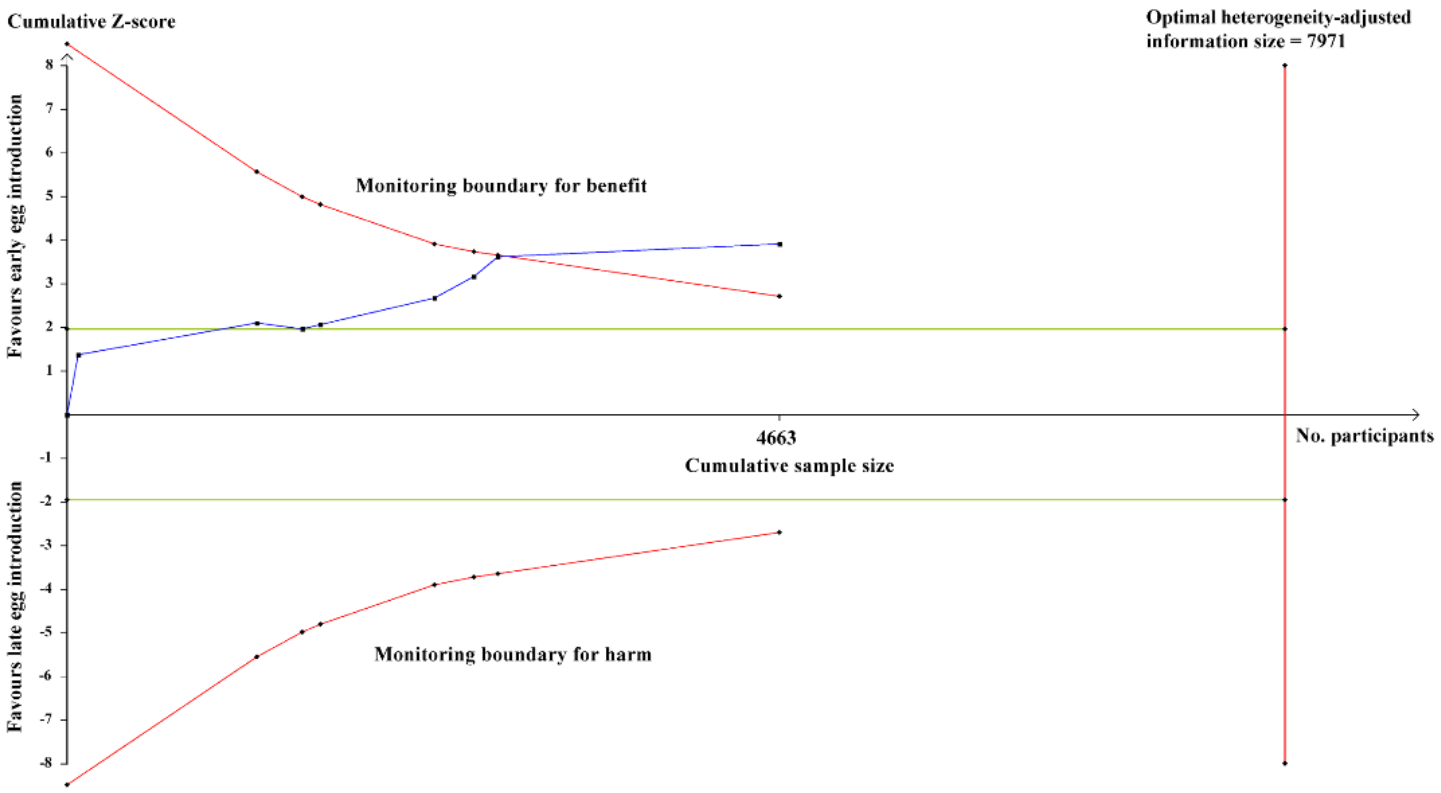


© 2023 Scarpone R et al. *JAMA Pediatrics*.

C

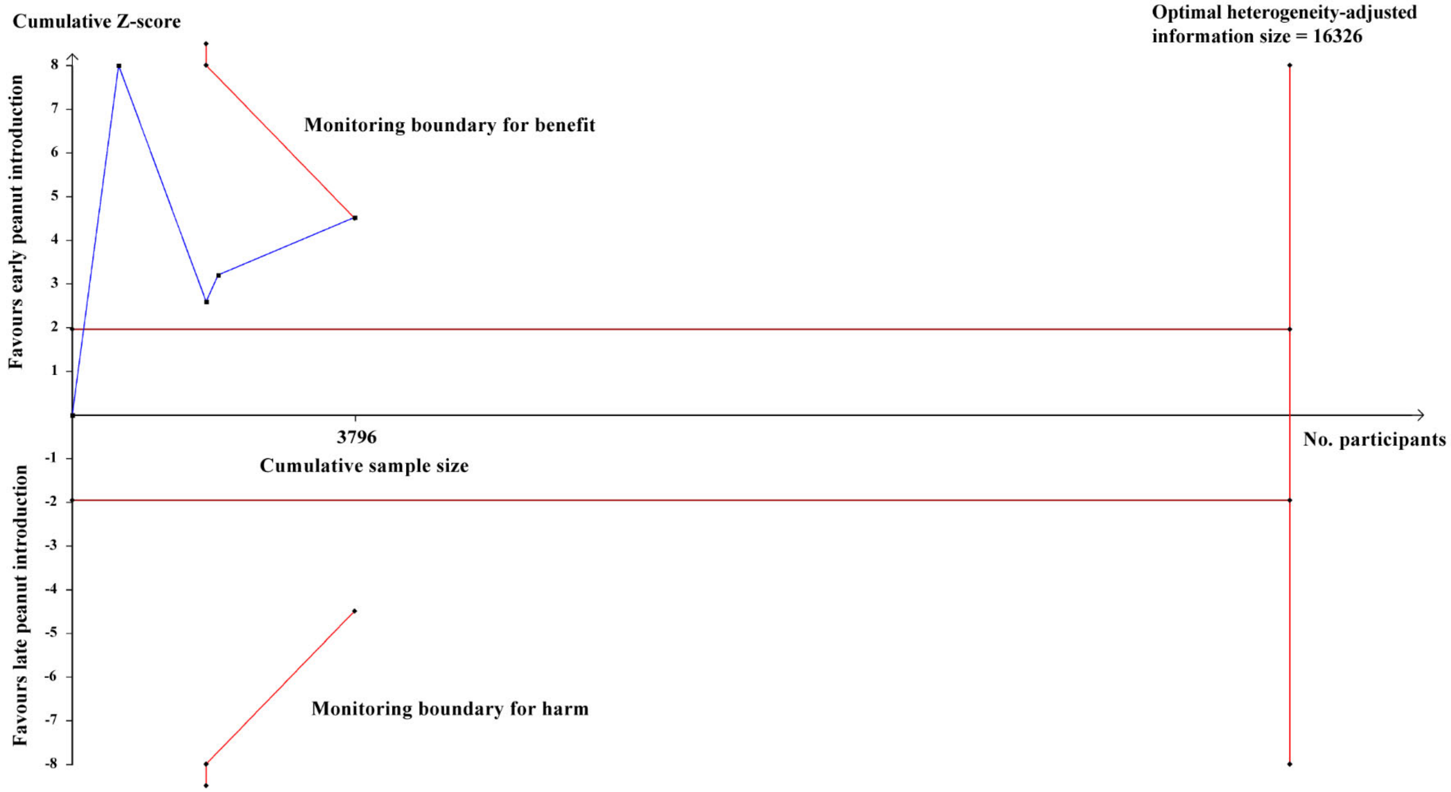


Trial sequential analysis is shown for earlier introduction of multiple allergenic foods and allergy to any food (A), earlier introduction of egg and egg allergy (B) and earlier introduction of peanut and peanut allergy (C). Horizontal lines are z scores of +1.96 or -1.96, equal to two-sided P=0.05. Datapoints on the blue (Z-statistic) and monitoring boundary lines represent individual studies in date and alphabetical order. The cumulative Z-statistic (blue line) approaches the trial sequential monitoring boundary for benefit in parts A and C, indicating that the optimal information size has not yet been reached for determining whether earlier introduction of multiple allergenic foods or peanut reduce risk of any food allergy or peanut allergy respectively, by ≥30%. For earlier egg introduction and egg allergy (B), the cumulative Z-statistic (blue line) crosses the trial sequential monitoring boundary for benefit, indicating that there is sufficient evidence to determine that earlier egg introduction reduces risk of egg allergy by ≥30%.

© 2023 Scarpone R et al. *JAMA Pediatrics*.

## eFigure 3. Earlier introduction of Allergenic Foods and Allergic Sensitization

### A

| Study | Earlier Events | Earlier Total | Later Events | Later Total | Weight | RR [95% CI] | D1 | D2 | D3 | D4 | D5 |
|---|---|---|---|---|---|---|---|---|---|---|---|
| Nishimura 2022 (29) | 65 | 76 | 68 | 74 | 45.5% | 0.93 [0.83, 1.04] | + | + | + | + | + |
| Perkin 2016 (32) | 51 | 572 | 61 | 601 | 32.5% | 0.88 [0.62, 1.25] | + | + | + | + | + |
| Skjerven 2022 (35) | 17 | 765 | 39 | 739 | 22.0% | 0.42 [0.24, 0.74] | + | + | + | + | + |
| **Overall** | **133** | **1413** | **168** | **1414** | **100.0%** | **0.77 [0.54, 1.10]** | | | | | |

Heterogeneity: $Tau^2 = 0.07$; $Chi^2 = 7.40$, df = 2 (P = 0.02); $I^2 = 73\%$

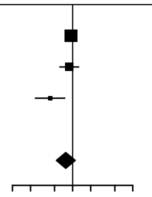


### B

| Study | Earlier Events | Earlier Total | Later Events | Later Total | Weight | RR [95% CI] | D1 | D2 | D3 | D4 | D5 |
|---|---|---|---|---|---|---|---|---|---|---|---|
| Bellach 2017 (14) | 8 | 142 | 4 | 156 | 2.0% | 2.20 [0.68, 7.14] | + | + | ? | + | ? |
| Makrides 2002 (25) | 7 | 91 | 7 | 46 | 2.8% | 0.51 [0.19, 1.35] | + | ? | − | + | ? |
| Nishimura 2022 (29) | 62 | 76 | 66 | 74 | 47.2% | 0.91 [0.80, 1.04] | + | + | + | + | + |
| Palmer 2013 (30) | 19 | 42 | 22 | 35 | 12.9% | 0.72 [0.47, 1.09] | + | + | + | + | + |
| Palmer 2017 (31) | 40 | 371 | 57 | 377 | 15.2% | 0.71 [0.49, 1.04] | + | + | + | + | + |
| Perkin 2016 (32) | 29 | 568 | 37 | 599 | 10.6% | 0.83 [0.52, 1.33] | + | + | + | + | + |
| Skjerven 2022 (35) | 8 | 765 | 9 | 739 | 3.0% | 0.86 [0.33, 2.21] | + | + | + | + | + |
| Tan 2017 (37) | 13 | 122 | 25 | 122 | 6.5% | 0.52 [0.28, 0.97] | + | + | + | + | ? |
| **Overall** | **186** | **2177** | **227** | **2148** | **100.0%** | **0.81 [0.69, 0.96]** | | | | | |

Heterogeneity: $Tau^2 = 0.01$; $Chi^2 = 8.55$, df = 7 (P = 0.29); $I^2 = 18\%$

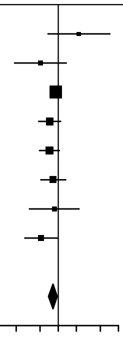


### C

| Study | Earlier Events | Earlier Total | Later Events | Later Total | Weight | RR [95% CI] | D1 | D2 | D3 | D4 | D5 |
|---|---|---|---|---|---|---|---|---|---|---|---|
| Du Toit 2018 (18) | 89 | 308 | 83 | 309 | 30.8% | 1.08 [0.83, 1.39] | + | + | + | ? | + |
| Nishimura 2022 (29) | 24 | 74 | 22 | 71 | 25.3% | 1.05 [0.65, 1.69] | + | + | + | + | + |
| Perkin 2016 (32) | 22 | 569 | 34 | 599 | 24.1% | 0.68 [0.40, 1.15] | + | + | + | + | + |
| Skjerven 2022 (35) | 10 | 765 | 33 | 739 | 19.7% | 0.29 [0.15, 0.59] | + | + | + | + | + |
| **Overall** | **145** | **1716** | **172** | **1718** | **100.0%** | **0.74 [0.46, 1.20]** | | | | | |

Heterogeneity: $Tau^2 = 0.18$; $Chi^2 = 13.24$, df = 3 (P = 0.00); $I^2 = 77\%$

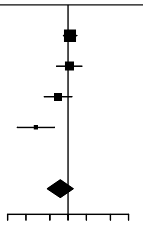


© 2023 Scarpone R et al. *JAMA Pediatrics*.

**D**

| Study | Earlier Events | Earlier Total | Later Events | Later Total | Weight | RR [95% CI] | Risk of Bias D1 | D2 | D3 | D4 | D5 |
|---|---|---|---|---|---|---|---|---|---|---|---|
| Du Toit 2018 (18) | 124 | 313 | 131 | 316 | 32.2% | 0.96 [0.79, 1.15] | + | + | + | ? | ? |
| Nishimura 2022 (29) | 65 | 76 | 68 | 74 | 39.9% | 0.93 [0.83, 1.04] | + | + | + | + | + |
| Perkin 2016 (32) | 51 | 572 | 61 | 601 | 18.3% | 0.88 [0.62, 1.25] | + | + | + | + | + |
| Skjerven 2022 (35) | 17 | 765 | 39 | 739 | 9.6% | 0.42 [0.24, 0.74] | + | + | + | + | + |
| **Overall** | **257** | **1726** | **299** | **1730** | **100.0%** | **0.86 [0.71, 1.05]** | | | | | |

Heterogeneity: $Tau^2 = 0.02$; $Chi^2 = 7.70$, df = 3 (P = 0.05); $I^2 = 61\%$

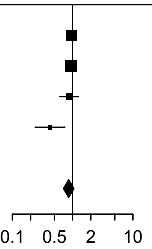


**E**

| Study | Earlier Events | Earlier Total | Later Events | Later Total | Weight | RR [95% CI] | Risk of Bias D1 | D2 | D3 | D4 | D5 |
|---|---|---|---|---|---|---|---|---|---|---|---|
| De Jong 1998 (16) | 41 | 702 | 30 | 732 | 22.1% | 1.43 [0.90, 2.26] | + | + | + | + | ? |
| Kjellman 1979 (23) | 4 | 25 | 4 | 23 | 5.8% | 0.92 [0.26, 3.26] | ? | + | + | ? | ? |
| Lowe 2011 (24) | 6 | 158 | 8 | 145 | 8.0% | 0.69 [0.24, 1.94] | ? | - | ? | + | ? |
| Nishimura 2022 (29) | 44 | 74 | 44 | 71 | 31.1% | 0.96 [0.74, 1.25] | + | + | + | + | + |
| Perkin 2016 (32) | 6 | 568 | 11 | 599 | 8.6% | 0.58 [0.21, 1.55] | + | + | + | + | + |
| Skjerven 2022 (35) | 1 | 765 | 1 | 739 | 1.4% | 0.97 [0.06, 15.42] | + | + | + | + | + |
| Urashima 2019 (38) | 46 | 143 | 24 | 143 | 23.1% | 1.92 [1.24, 2.96] | + | + | + | + | + |
| **Overall** | **148** | **2435** | **122** | **2452** | **100.0%** | **1.14 [0.82, 1.59]** | | | | | |

Heterogeneity: $Tau^2 = 0.07$; $Chi^2 = 10.91$, df = 6 (P = 0.09); $I^2 = 45\%$

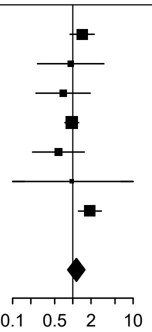


Data shown are for earlier introduction of multiple allergenic foods and allergic sensitization to any food (A), earlier introduction of egg and allergic sensitization to egg (B), earlier introduction of peanut and allergic sensitization to peanut (C) or to any food (D) and earlier introduction of cow's milk and allergic sensitization to cow's milk (E). There was no information for sensitization to any food from trials of earlier egg or milk introduction without multiple allergenic food introduction. Risk of bias D1, randomization process; D2, deviations from the intended intervention; D3, missing outcome data; D4, measurement of the outcome; D5, selection of the reported result; (+), low risk of bias; (?), some concerns; (-), high risk of bias; RR, risk ratio; CI, confidence interval.

© 2023 Scarpone R et al. *JAMA Pediatrics*.

## eFigure 4. Earlier Introduction of Allergenic Foods and Withdrawal From Study Intervention

### A

| Study | Earlier Events | Earlier Total | Later Events | Later Total | Weight | RR [95% CI] | D1 | D2 | D3 | D4 | D5 |
|---|---|---|---|---|---|---|---|---|---|---|---|
| Bellach 2017 (14) | 60 | 184 | 47 | 199 | 10.2% | 1.38 [1.00, 1.91] | + | + | + | + | ? |
| Holl 2020 (20) | 45 | 366 | 41 | 339 | 9.8% | 1.02 [0.68, 1.51] | ? | + | + | + | ? |
| Iannotti 2017 (21) | 4 | 80 | 9 | 83 | 5.0% | 0.46 [0.15, 1.44] | + | + | + | − | ? |
| Makrides 2002 (25) | 15 | 106 | 9 | 55 | 7.3% | 0.86 [0.40, 1.85] | + | + | + | − | ? |
| Natsume 2017 (28) | 14 | 73 | 13 | 74 | 7.8% | 1.09 [0.55, 2.16] | ? | + | + | + | + |
| Nishimura 2022 (29) | 1 | 83 | 1 | 80 | 1.4% | 0.96 [0.06, 15.15] | + | + | + | + | + |
| Palmer 2013 (30) | 20 | 49 | 5 | 37 | 6.5% | 3.02 [1.25, 7.30] | + | + | + | + | + |
| Palmer 2017 (31) | 59 | 407 | 48 | 413 | 10.0% | 1.25 [0.87, 1.78] | + | + | + | + | + |
| Perkin 2016 (32) | 432 | 652 | 117 | 651 | 10.9% | 3.69 [3.10, 4.38] | + | + | + | − | + |
| Quake 2022 (33) | 1 | 90 | 1 | 45 | 1.4% | 0.50 [0.03, 7.81] | ? | + | + | ? | ? |
| Skjerven 2022 (35) | 871 | 1225 | 249 | 1172 | 11.1% | 3.35 [2.98, 3.76] | + | + | + | − | + |
| Stewart 2019 (36) | 41 | 331 | 24 | 329 | 9.2% | 1.70 [1.05, 2.74] | + | + | + | − | + |
| Tan 2017 (37) | 42 | 165 | 22 | 154 | 9.3% | 1.78 [1.12, 2.84] | + | + | + | + | + |
| **Overall** | **1605** | **3811** | **586** | **3631** | **100.0%** | **1.58 [1.12, 2.22]** | | | | | |

Heterogeneity: $Tau^2 = 0.27$; $Chi^2 = 116.73$, df = 12 (P = 0.00); $I^2 = 90\%$

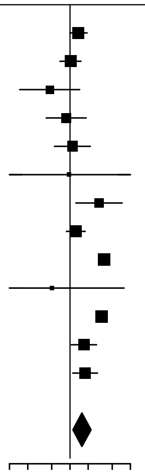


### B

| Study | Earlier Events | Earlier Total | Later Events | Later Total | Weight | RR [95% CI] | D1 | D2 | D3 | D4 | D5 |
|---|---|---|---|---|---|---|---|---|---|---|---|
| Du Toit 2015 (17) | 25 | 319 | 26 | 321 | 19.9% | 0.97 [0.57, 1.64] | + | + | + | − | + |
| Holl 2020 (20) | 45 | 366 | 41 | 339 | 22.3% | 1.02 [0.68, 1.51] | ? | + | + | + | ? |
| Nishimura 2022 (29) | 1 | 83 | 1 | 80 | 2.6% | 0.96 [0.06, 15.15] | + | + | + | + | + |
| Perkin 2016 (32) | 432 | 652 | 117 | 651 | 25.6% | 3.69 [3.10, 4.38] | + | + | + | − | + |
| Quake 2022 (33) | 2 | 90 | 1 | 45 | 3.4% | 1.00 [0.09, 10.74] | ? | + | + | ? | ? |
| Skjerven 2022 (35) | 871 | 1225 | 249 | 1172 | 26.1% | 3.35 [2.98, 3.76] | + | + | + | − | + |
| **Overall** | **1376** | **2735** | **435** | **2608** | **100.0%** | **1.91 [1.19, 3.05]** | | | | | |

Heterogeneity: $Tau^2 = 0.22$; $Chi^2 = 56.24$, df = 5 (P = 0.00); $I^2 = 91\%$

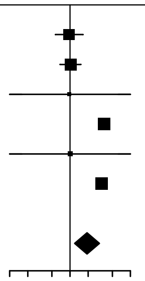


© 2023 Scarpone R et al. *JAMA Pediatrics*.

**C**

| Study | Earlier Events | Earlier Total | Later Events | Later Total | Weight | RR [95% CI] | D1 | D2 | D3 | D4 | D5 |
|---|---|---|---|---|---|---|---|---|---|---|---|
| Brown 1969 (15) | 0 | 196 | 85 | 183 | 3.1% | 0.01 [0.00, 0.09] | ? | + | + | − | ? |
| De Jong 1998 (16) | 51 | 758 | 60 | 775 | 14.3% | 0.87 [0.61, 1.25] | + | + | + | + | ? |
| Holl 2020 (20) | 45 | 366 | 41 | 339 | 14.1% | 1.02 [0.68, 1.51] | ? | + | + | + | ? |
| Kjellman 1979 (23) | 1 | 26 | 1 | 24 | 3.2% | 0.92 [0.06, 13.95] | ? | + | + | − | ? |
| Lowe 2011 (24) | 13 | 206 | 17 | 208 | 12.2% | 0.77 [0.38, 1.55] | ? | + | + | + | ? |
| Nishimura 2022 (29) | 1 | 83 | 1 | 80 | 3.1% | 0.96 [0.06, 15.15] | + | + | + | + | + |
| Perkin 2016 (32) | 432 | 652 | 117 | 651 | 15.0% | 3.69 [3.10, 4.38] | + | + | + | − | + |
| Quake 2022 (33) | 1 | 90 | 1 | 45 | 3.1% | 0.50 [0.03, 7.81] | ? | + | + | ? | ? |
| Sakihara 2021 (34) | 37 | 252 | 48 | 252 | 14.1% | 0.77 [0.52, 1.14] | − | + | + | − | ? |
| Skjerven 2022 (35) | 871 | 1225 | 249 | 1172 | 15.1% | 3.35 [2.98, 3.76] | + | + | + | − | + |
| Urashima 2019 (38) | 0 | 156 | 3 | 156 | 2.8% | 0.14 [0.01, 2.74] | + | + | + | − | + |
| **Overall** | **1452** | **4010** | **623** | **3885** | **100.0%** | **1.05 [0.61, 1.82]** | | | | | |

Heterogeneity: $Tau^2 = 0.51$; $Chi^2 = 164.50$, df = 10 (P = 0.00); $I^2 = 94\%$

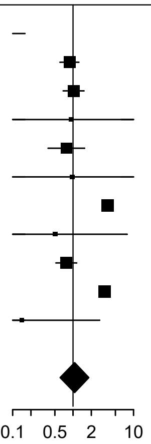


Data shown are for the outcome withdrawal from study intervention, in trials of earlier egg (A), peanut (B) or cow's milk (C) introduction. Risk of bias D1, randomization process; D2, deviations from the intended intervention; D3, missing outcome data; D4, measurement of the outcome; D5, selection of the reported result; (+), low risk of bias; (?), some concerns; (-), high risk of bias; RR, risk ratio; CI, confidence interval.

© 2023 Scarpone R et al. *JAMA Pediatrics*.

**eFigure 5. Exploration of Small Study Effects Using Funnel Plots**

**A**

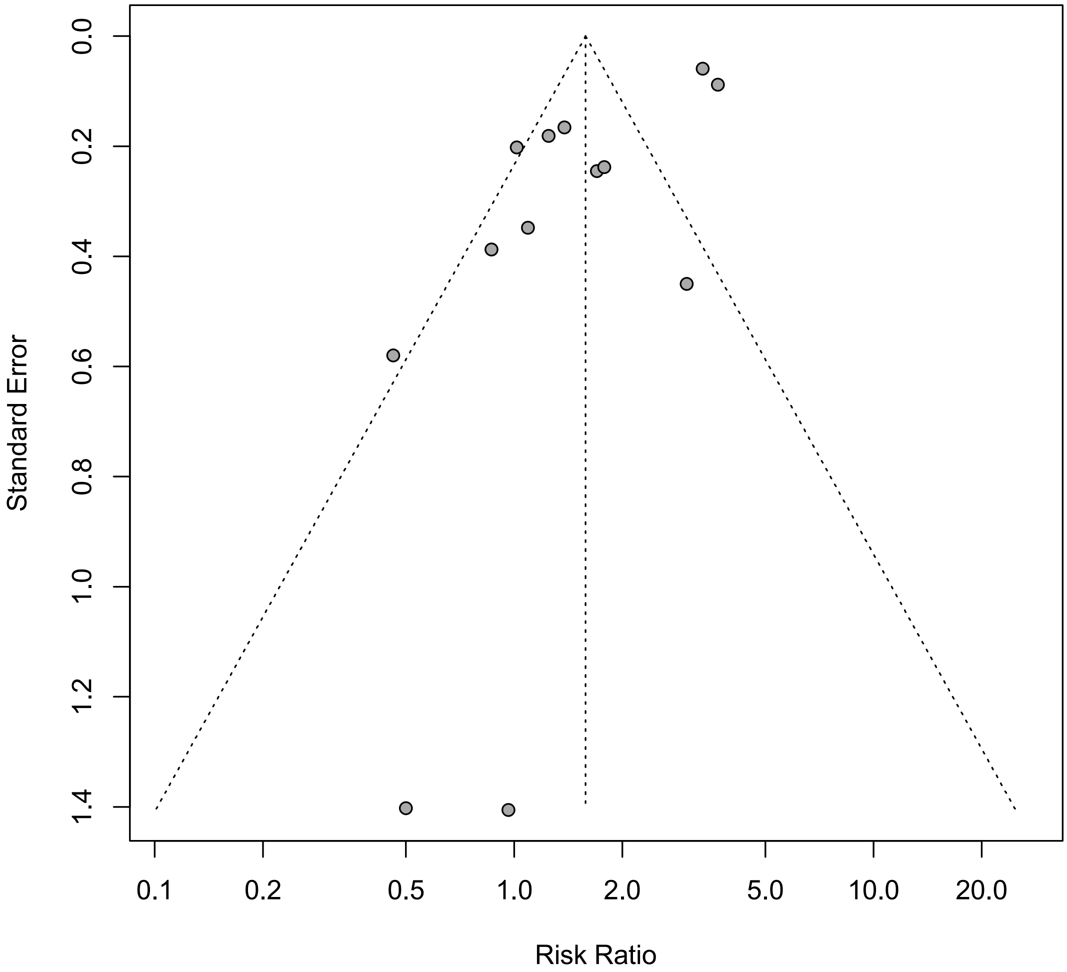


© 2023 Scarpone R et al. *JAMA Pediatrics*.

**B**

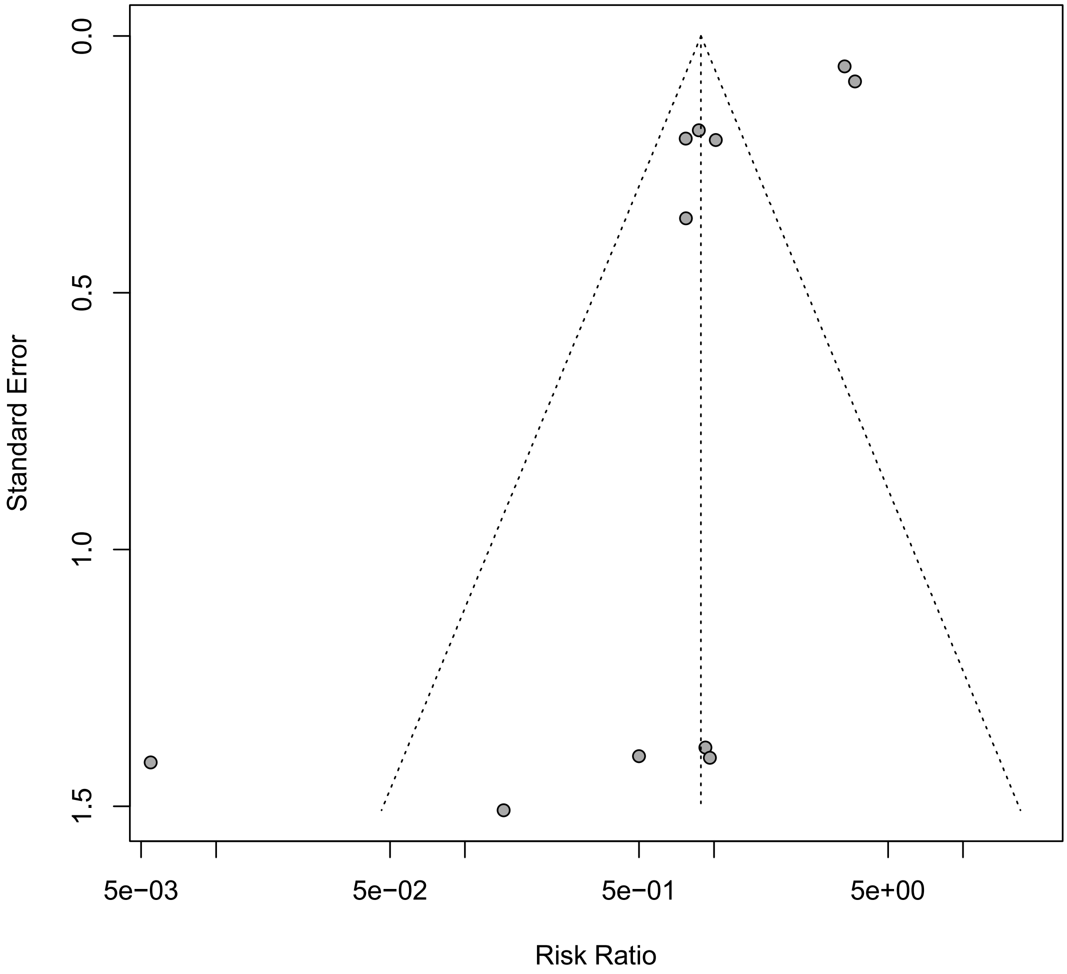


Funnel plots are shown for the outcome withdrawal from study intervention, in trials of earlier egg (A) or cow's milk (B) introduction. Funnel plots were only undertaken for meta-analyses including data from at least 10 trials. No other meta-analysis included 10 or more trials. Data show evidence of asymmetry for both egg (Egger's test, P=0.004) and cow's milk (Egger's test, P=0.01).

© 2023 Scarpone R et al. *JAMA Pediatrics*.

## eFigure 6. Earlier Introduction of Allergenic Foods and Risk of Allergy to Any Food

### A

| Study | Earlier Events | Earlier Total | Later Events | Later Total | Weight | RR [95% CI] | Risk of Bias D1 | D2 | D3 | D4 | D5 |
|---|---|---|---|---|---|---|---|---|---|---|---|
| Nishimura 2022 (29) | 7 | 82 | 19 | 79 | 16.6% | 0.35 [0.16, 0.80] | + | + | + | + | + |
| Perkin 2016 (32) | 32 | 567 | 42 | 595 | 32.5% | 0.80 [0.51, 1.25] | + | + | + | ? | + |
| Quake 2022 (33) | 18 | 89 | 21 | 44 | 28.5% | 0.42 [0.25, 0.71] | ? | + | + | ? | ? |
| Skjerven 2022 (35) | 13 | 924 | 31 | 915 | 22.5% | 0.42 [0.22, 0.79] | + | + | + | + | + |
| **Overall** | **70** | **1662** | **113** | **1633** | **100.0%** | **0.50 [0.34, 0.75]** | | | | | |

Heterogeneity: $Tau^2 = 0.07$; $Chi^2 = 5.50$, df = 3 (P = 0.14); $I^2 = 45\%$

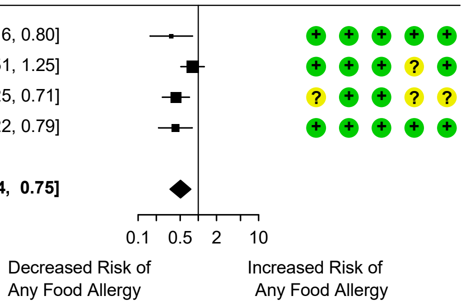


### B

| Study | Earlier Events | Earlier Total | Later Events | Later Total | Weight | RR [95% CI] | Risk of Bias D1 | D2 | D3 | D4 | D5 |
|---|---|---|---|---|---|---|---|---|---|---|---|
| Du Toit 2018 (18) | 180 | 316 | 179 | 317 | 26.5% | 1.01 [0.88, 1.16] | + | + | + | − | ? |
| Nishimura 2022 (29) | 7 | 82 | 19 | 79 | 14.4% | 0.35 [0.16, 0.80] | + | + | + | + | + |
| Perkin 2016 (32) | 32 | 567 | 42 | 595 | 21.4% | 0.80 [0.51, 1.25] | + | + | + | ? | + |
| Quake 2022 (33) | 19 | 88 | 21 | 44 | 20.2% | 0.45 [0.27, 0.75] | ? | + | + | ? | ? |
| Skjerven 2022 (35) | 13 | 924 | 31 | 915 | 17.5% | 0.42 [0.22, 0.79] | + | + | + | + | + |
| **Overall** | **251** | **1977** | **292** | **1950** | **100.0%** | **0.60 [0.38, 0.94]** | | | | | |

Heterogeneity: $Tau^2 = 0.19$; $Chi^2 = 20.78$, df = 4 (P = 0.00); $I^2 = 81\%$

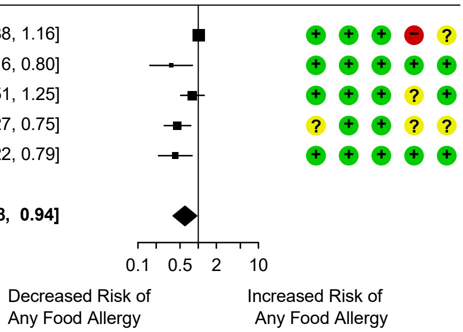


### C

| Study | Earlier Events | Earlier Total | Later Events | Later Total | Weight | RR [95% CI] | Risk of Bias D1 | D2 | D3 | D4 | D5 |
|---|---|---|---|---|---|---|---|---|---|---|---|
| Lowe 2011 (24) | 26 | 193 | 37 | 191 | 17.7% | 0.70 [0.44, 1.10] | ? | + | + | + | ? |
| Nishimura 2022 (29) | 7 | 82 | 19 | 79 | 13.8% | 0.35 [0.16, 0.80] | + | + | + | + | + |
| Perkin 2016 (32) | 32 | 567 | 42 | 595 | 17.9% | 0.80 [0.51, 1.25] | + | + | + | ? | + |
| Quake 2022 (33) | 18 | 89 | 21 | 44 | 17.1% | 0.42 [0.25, 0.71] | ? | + | + | ? | ? |
| Skjerven 2022 (35) | 13 | 924 | 31 | 915 | 15.7% | 0.42 [0.22, 0.79] | + | + | + | + | + |
| Urashima 2019 (38) | 45 | 151 | 22 | 151 | 17.7% | 2.05 [1.29, 3.23] | + | + | + | − | + |
| **Overall** | **141** | **2006** | **172** | **1975** | **100.0%** | **0.67 [0.39, 1.13]** | | | | | |

Heterogeneity: $Tau^2 = 0.36$; $Chi^2 = 29.93$, df = 5 (P = 0.00); $I^2 = 83\%$

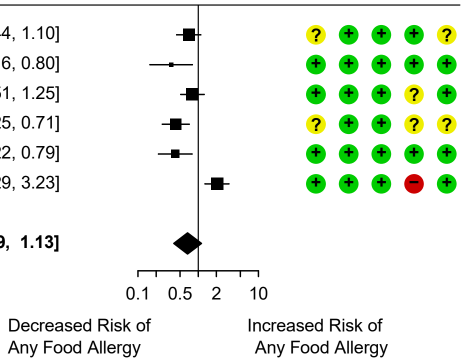


Data shown are for the outcome "allergy to any food", in trials of earlier egg (A), peanut (B) and cow's milk (C) introduction. Risk of bias D1, randomization process; D2, deviations from the intended intervention; D3, missing outcome data; D4, measurement of the outcome; D5, selection of the reported result; (+), low risk of bias; (?), some concerns; (-), high risk of bias; RR, risk ratio; CI, confidence interval.

© 2023 Scarpone R et al. *JAMA Pediatrics.*

**eTable 1. Characteristics of Included Studies**

| Study | Population | Intervention | Outcome assessment | Funding source and any relevant author conflicts of interest |
|---|---|---|---|---|
| **Bellach et al,[14] 2017**<br><br>**Hen's Egg Allergy Prevention (HEAP)**<br><br>**Germany** | Infants aged 4 to 6 months with egg-sIgE <0.35kU/L | Pasteurized egg white powder (n=184) vs rice powder (n=199) 3 times a week up to 12 months. 2.5g (week 1), 5g (week 2), and 7.5g (from week 3) egg protein per week. | Egg allergy: single-dose OFC (egg group) and graded DBPCFC (placebo group) in sensitized children at 12 months.<br>Egg sensitization: egg-sIgE ≥0.35kU/L at 12 months<br>Withdrawal: participants not evaluated in per-protocol analysis. | Food Allergy & Anaphylaxis Network and Prevention and Information Network for Allergy/Asthma, Germany. Authors reported payments from formula milk companies. |
| **Brown et al,[15] 1969**<br><br>**USA** | Infants from birth at standard risk for atopic disease | Cow's milk (n=196) vs milk-free diet with a soya formula as required (n=183) until weaning | Withdrawal: participants who did not accept milk-free diet | National Institute of Child Health and Human Development |
| **De Jong et al,[16] 1998**<br><br>**BOKAAL**<br><br>**Netherlands** | Infants from birth whose mothers intended to breastfeed for ≥6 weeks | Cow's milk formula (11.1g protein/100g powder) ≥3 times in the first 3 days of life (n=758) vs protein-free placebo formula (n=775).<br>One sachet of formula powder mixed with 60ml of water. Median 120ml in the first week. | Milk sensitization: milk-sIgE ≥2+RAST at 12 months<br>Withdrawal: participants who did not adhere to the protocol | Nutricia Nederland BV, Zoetermeer, the Netherlands |

© 2023 Scarpone R et al. *JAMA Pediatrics*.

| Study | Population | Intervention | Outcome assessment | Funding source and any relevant author conflicts of interest |
|---|---|---|---|---|
| **Du Toit et al,[17] 2015**<br>**Du Toit et al,[18] 2018**<br><br>**Learning Early About Peanut Allergy (LEAP)**<br><br>**UK** | Infants aged 4 to 10 months with severe eczema and/or egg allergy and peanut-SPT <4mm | ≥6g peanut protein per week (peanut snack or peanut butter), divided between ≥3 meals, up to 5 years of age (n=319) vs peanut avoidance (n=321) | Peanut allergy: single-dose OFC (if peanut allergy unlikely) or graded DBPCFC (other participants) or diagnostic algorithm (11 participants) at 60 months<br>Any food allergy: reported allergic reaction to any food in or outside clinic up to 60 months<br>Peanut sensitization: peanut-sIgE ≥0.35kU/L or peanut-SPT ≥3mm at 30 months<br>Any food sensitization: SPT ≥5mm at 60 months<br>Withdrawal: participants not evaluated in per-protocol analysis | National Institute of Allergy and Infectious Diseases, Food Allergy Research and Education, Medical Research Council and Asthma UK, UK Department of Health, National Peanut Board, and UK Food Standards Agency. |
| **Halpern et al,[19] 1973**<br><br>**USA** | Infants from birth at standard risk for atopic disease | Egg yolk given before 3 weeks vs after 6 months (n=1753, precise number per group not clear) | Egg allergy: participants with "symptoms", but precise outcome definition was not available | Borden, Inc, New York, NY |
| **Holl et al,[20] 2020**<br><br>**USA** | Infants aged 5 to 11 months without parent-reported severe eczema or food allergy diagnosis | Daily, single-dose, powdered food supplement containing 30mg of protein from each of peanut, soya, almond, cashew, hazelnut, pecan, pistachio, walnut, wheat, oat, milk, egg, cod, shrimp, salmon, and sesame (n=366) vs powdered placebo (n=339) for 28 days | Withdrawal: participants who were non-compliant with recording doses in the daily diary | Before Brands, Inc |
| **Iannotti et al,[21] 2017**<br><br>**Lulun Project**<br><br>**Ecuador** | Infants aged 6 to 9 months without egg allergy.<br>Egg consumption at baseline 40% (egg group) and 45% (control). | One medium-sized egg (≈50g) per day for 6 months (n=80) vs no intervention (n=83) | Egg allergy: observed or reported allergenic reaction to egg during the intervention period<br>Withdrawal: participants not analyzed at end point, excluding those who "moved permanently" | The Mathile Institute for the Advancement of Human Nutrition |

© 2023 Scarpone R et al. *JAMA Pediatrics*.

| Study | Population | Intervention | Outcome assessment | Funding source and any relevant author conflicts of interest |
|---|---|---|---|---|
| **Johnstone and Dutton,[22] 1966**<br><br>**USA** | Infants from birth with a family history of atopic disease | Cow's milk (formula) as required and wheat avoidance (n=120) vs soya formula and avoidance of cow products, chicken, egg, and wheat (n=115) for 9 months | Any food allergy: precise allergy definition was not available | United States Atomic Energy Commission at the University of Rochester Atomic Energy Project |
| **Kjellman and Johansson,[23] 1979**<br><br>**Sweden** | Infants from birth with a family history of atopic disease | Cow's milk formula (n=26) vs soya formula (n=24) as required and fish, egg, chocolate, and strawberry avoidance for 9 months | Milk allergy: observed symptoms to milk up to 48 months<br>Milk and soya sensitization: sIgE ≥0.2PRU at 36 months<br>Withdrawal: participants who refused further participation or developed symptoms | Förenade Liv, Stockholm, Semper Fund for Nutritional Research, Stockholm, King Gustaf V 80-Years-Anniversary Fund, the Medical Faculty, Linköping University and the Swedish Medical Research Council |
| **Lowe et al,[24] 2011**<br><br>**Melbourne Atopy Cohort Study (MACS)**<br><br>**Australia** | Infants from birth with a family history of atopic disease | Cow's milk formula (n=206) vs soya formula (n=208) as required. Dairy products, egg, fish, peanut, and nuts were avoided until 12 months. | Milk and any food allergy: reported reactions up to 24 months<br>Milk sensitization: milk-SPT ≥3mm at 24 months<br>Withdrawal: participants lost to follow-up at 24 months | Nestec Ltd.<br>Author conflicts of interest declared related to infant nutrition and dairy industries. |
| **Makrides et al,[25] 2002**<br><br>**Australia** | Infants aged 6 months without protein intolerances or allergies | Four regular or n-3 fatty acid-enriched egg yolks per week until 12 months (n=106) vs no dietary intervention (n=55) | Egg sensitization: egg-sIgE ≥0.35kU/L at 12 months<br>Withdrawal: participants withdrawn for reasons possibly related to the intervention | Rural Industries Research and Development Corporation, National Health & Medical Research Council, and the MS McLeod Research Trust |
| **Miskelly et al,[26] 1988**<br>**Hand et al,[27] 2021**<br><br>**Merthyr Allergy Prevention Study (MAPS)**<br><br>**UK** | Infants from birth with a family history of atopic disease | Cow's milk (n≈238) vs milk-free diet with a soya formula as required (n≈249) for ≥4 months. Mothers to restrict milk intake during pregnancy and lactation in the milk avoidance group. | Withdrawal: participants who received the wrong type of milk, but numbers were inconsistent between study publications and denominators were not clear | Welsh Scheme for the Development of Health and Social Research and Wyeth Laboratories |

© 2023 Scarpone R et al. *JAMA Pediatrics*.

| Study | Population | Intervention | Outcome assessment | Funding source and any relevant author conflicts of interest |
|---|---|---|---|---|
| **Natsume et al,[28] 2017**<br><br>**Prevention of Egg allergy with Tiny amount InTake (PETIT)**<br><br>**Japan** | Infants aged 4 to 5 months with atopic dermatitis | Heated egg powder (50mg containing 25mg egg protein daily from 6 to 9 months; 250mg containing 125mg egg protein daily from 9 to 12 months; n=73) vs placebo (pumpkin powder) from 6 to 12 months (n=74). | Egg allergy: single-dose OFC at 12 months (both groups)<br>Withdrawal: participants not evaluated in per-protocol analysis, unless reason given was clearly unrelated to the intervention | Ministry of Health, Labour and Welfare and National Centre for Child Health and Development, Japan |
| **Nishimura et al,[29] 2022**<br><br>**Japan** | Infants aged 3 to 4 months with atopic dermatitis | Daily mixed powder containing 2.5, 7.5, 20mg of each food (mg protein): pasteurized egg (2.2, 6.6, 17.6), milk (0.3, 0.9, 2.4), wheat (0.2, 0.6, 1.6), soya (0.9, 2.7, 7.2), buckwheat (0.3, 0.9, 2.4), and peanut (0.6, 1.8, 4.8; n=83) vs increasing amounts of placebo powder (n=80) up to week 2, 4, 12. Egg, milk, wheat, soya, buckwheat, and peanut avoidance to week 12. | Any food, egg, peanut, milk, wheat, and soya allergy: parental report or OFC in sensitized children from end of intervention to 18 months<br>Any food, egg, peanut, milk, wheat, and soya sensitization: sIgE >0.1kU/L at 11 to 13 months<br>Withdrawal: participants who refused to continue intervention or dropped out during follow-up | No funding received and no declared conflicts of interest |
| **Palmer et al,[30] 2013**<br><br>**Solids Timing for Allergy Reduction (STAR)**<br><br>**Australia** | Infants aged 4 months with moderate-to-severe eczema (SCORAD score of ≥15) | One teaspoon of pasteurized raw whole egg powder (0.9g egg protein; n=49) vs rice flour powder (n=37) daily until 8 months. Introduction of cooked egg in both groups at 8 months. | Egg allergy: if no obvious prior reaction, single-dose (if low-risk) or graded (if high-risk) pasteurized raw egg challenge in sensitized children at 12 months<br>Egg sensitization: egg-SPT ≥3mm at 12 months<br>Withdrawal: participants who withdrew from study for a reason other than “moving overseas”, or who ceased powder due to an allergic reaction | Women’s and Children’s Hospital Foundation and Ilhan Food Allergy Foundation.<br>Senior author reported being on the Boards for Nestlé, Danone, and ALK-Abelló. |

© 2023 Scarpone R et al. *JAMA Pediatrics*.

| Study | Population | Intervention | Outcome assessment | Funding source and any relevant author conflicts of interest |
|---|---|---|---|---|
| **Palmer et al,[31] 2017**<br><br>**Starting Time of Egg Protein (STEP)**<br><br>**Australia** | Infants aged 4 to 6 months with atopic mothers, but without history of allergic disease or ingestion of egg, who had not commenced solids before age 4 months | Pasteurized raw whole egg powder (0.4g egg protein; n=407) vs rice powder (n=413) daily until 10 months. | Egg allergy: single-dose (if low-risk) or graded (if high-risk) pasteurized raw egg challenge in sensitized children at 12 months<br>Egg sensitization: egg-SPT ≥3mm at 12 months<br>Withdrawal: participants with missing outcome data, who “did not take any powder”, and who “had a confirmed reaction to study powder” | Australian National Health and Medical Research Council.<br>First author reported payments from Nestlé Nutrition and Danone. Senior author reported serving on the board for Fonterra, and as a consultant for Nestlé and True Origins. |
| **Perkin et al,[32] 2016**<br><br>**Enquiring About Tolerance (EAT)**<br><br>**UK** | Infants aged 3 months, exclusively breastfed | Sequential introduction of cow’s milk (first), peanut, egg, sesame, fish (in random order), and wheat (last), 2g of the allergen protein twice weekly (n=652) vs exclusive breastfeeding to ≥6 months (n=651) | Any food, egg, peanut, milk, wheat, and fish allergy: mostly graded DBPCFC or reported reaction in sensitized children up to 36 months<br>Any food, egg, peanut, milk, wheat, and fish sensitization: SPT ≥0 at 36 months<br>Withdrawal: participants not evaluated in per-protocol analysis except if due to “emigration” | Food Standards Agency, Medical Research Council, NIHR Biomedical Research Centre, and National Peanut Board, Atlanta. |
| **Quake et al,[33] 2022**<br><br>**USA** | Infants aged 2 to 12 months with or without a family history of atopic disease, without known food allergy | Flours and powders of egg, milk, or peanut (each 300mg protein), or peanut and milk, peanut and egg, or milk and egg (each 150mg protein, 300mg per pair), or a 10-allergen mixture (almond, cashew, egg, hazelnut, milk, peanut, salmon, shrimp, walnut, and wheat, each 30, 90, or 300mg protein, 300, 900, or 3000mg per mixture) daily (n=135) vs control group which avoided all allergenic foods for 12 months (n=45). | Any food allergy: graded DBPCFC 24 to 48 months after the start of the study<br>Withdrawal: participants missing in the food allergy analysis | Sean N. Parker Center for Allergy and Asthma Research at Stanford University<br><br>Patent associated with the work “Special Oral Formula for Decreasing Food Allergy Risk and Treatment for Food Allergy” |

© 2023 Scarpone R et al. *JAMA Pediatrics*.

| Study | Population | Intervention | Outcome assessment | Funding source and any relevant author conflicts of interest |
|---|---|---|---|---|
| **Sakihara et al,[34] 2021**<br><br>**Strategy for Prevention of Milk Allergy by Daily Ingestion of Infant Formula in Early Infancy (SPADE)**<br><br>**Japan** | Infants aged 1 month with a negative OFC to cow's milk formula (20ml at once) who used cow's milk formula before 1 month | ≥10ml cow's milk formula (150mg protein) daily for ≥20 days per month with a maximum interruption of 1 week (n=252) vs cow's milk formula avoidance (<10 days per month) with soya formula as required (n=252) until 3 months while continuing breastfeeding. Avoidance of other dairy products, but cow's milk formula as required in both groups from 3 to 6 months. | Milk allergy: single-dose or if sensitized graded OFC at 6 months<br>Milk sensitization: milk-SPT ≥3mm at 6 months<br>Withdrawal: participants who declined or did not adhere to treatment or who withdrew except if they "moved" | Meiji Holdings Co, Ltd and Asahi Group Foods Ltd |
| **Skjerven et al,[35] 2022**<br><br>**Preventing Atopic Dermatitis and ALLergies in Children (PreventADALL)**<br><br>**Norway, Sweden** | Infants aged 3 to 4 months at standard risk for atopic disease | Sequential introduction of peanut, cow's milk, wheat, and egg (dose not specified) until ≥6 months with or without a skincare intervention (factorial design) from week 2 through 8 months (n=1225) vs no intervention or skincare intervention only (n=1172) | Any food, egg, peanut, milk, and wheat allergy: parental report and/or SPT result and/or graded OFC up to 36 months<br>Any food, egg, peanut, milk, and wheat sensitization: SPT ≥3mm at 36 months<br>Withdrawal: participants not evaluated in per-protocol analysis | Funded by several public and charitable funding bodies |
| **Stewart et al,[36] 2019**<br><br>**Mazira Project**<br><br>**Malawi** | Infants aged 6 to 9 months without egg allergy or severe allergic reaction to any substance, and no reaction to egg during a test feed. Baseline egg consumption≈4% | One egg per day for 6 months (n=331) vs control group that did not receive eggs (n=329) | Withdrawal: participants who withdrew or were absent | Bill and Melinda Gates Foundation |

© 2023 Scarpone R et al. *JAMA Pediatrics*.

| Study | Population | Intervention | Outcome assessment | Funding source and any relevant author conflicts of interest |
|---|---|---|---|---|
| **Tan et al,[37] 2017**<br><br>**Beating Egg Allergy Trial (BEAT)**<br><br>**Australia** | Infants aged 4 months with egg-SPT <2mm and a family history of atopic disease | Pasteurized whole egg powder (350mg protein; n=165) vs rice powder (n=154) daily from weaning until 8 months.<br>Egg avoidance until 8 months in both groups. | Egg allergy: reported reaction in sensitized children or graded OFC or egg-SPT ≥5mm at 12 months<br>Egg sensitization: egg-SPT ≥3mm at 12 months<br>Withdrawal: participants who withdrew or were lost to follow-up | Ilhan Food Allergy Foundation and Children's Hospital at Westmead Allergy and Immunology research fund |
| **Urashima et al,[38] 2019**<br><br>**Atopy Induced by Breastfeeding or Cow's Milk Formula (ABC)**<br><br>**Japan** | Infants from birth with a family history of atopic disease | Cow's milk formula within the first 24 hours of life (≥5ml followed by ≥40ml daily after 1 month) until 5 months or before starting solid foods (n=156) vs cow's milk formula avoidance for at least the first 3 days of life and amino acid-based elemental formula as required until 5 months (n=156). If ≥150ml of elemental formula were given daily for 3 consecutive days, it was switched to cow's milk formula after the fourth day. | Any food allergy and milk allergy: parental report or graded OFC in sensitized children up to 24 months<br>Milk sensitization: milk-sIgE ≥0.35kU/L at 24 months<br>Withdrawal: participants who did not adhere to the protocol or who were lost to follow-up | Funded by several public funding bodies, including the Dairy Products Health Science Council; and the Japan Dairy Association |

Abbreviations: sIgE, specific immunoglobulin E; DBPCFC, double-blind placebo-controlled food challenge; OFC, open food challenge; RAST, radioallergosorbent test; PRU, Phadebas RAST units; SCORAD, SCORing Atopic Dermatitis.

© 2023 Scarpone R et al. *JAMA Pediatrics*.

## eTable 2. Characteristics of Ongoing Studies

| Study | Population | Intervention | Outcomes | Funding source/ sponsor |
|---|---|---|---|---|
| **Study on the Induction of Food Tolerance in Babies (INTO)[39]**<br>**Registered Jul 7, 2016, at https://clinicaltrials.gov**<br>**NCT02825069**<br>**Finland** | Infants aged ≤1 month attending local Child Health Clinics<br>Target No. of participants: 1380 | Introduction of solid foods (vegetables and fruits, wheat and other grains, meat, fish, egg, dairy products) from 4 to 6 months vs official Finnish Nutrition Recommendations, including exclusive breastfeeding until the age of 6 months | Doctor-diagnosed and parent-reported food allergies by the age of 1 year | University of Oulu |
| **Prevention of infants' milk allergy (PIMA study)[40]**<br>**Registered Dec 1, 2017, at https://center6.umin.ac.jp**<br>**UMIN000030214**<br>**Japan** | Infants aged 6 to 11 months sensitized to milk and with a food allergy other than milk with no history of immediate symptoms with milk, no regular intake of >3ml milk, no non-IgE mediated gastrointestinal milk allergy and no severe, uncontrolled atopic dermatitis<br>Target No. of participants: 128 | Perform a 3ml heated milk oral food challenge and if tolerated, take 3ml milk at home regularly; vs milk avoidance to 18 months and introduction of 3ml daily milk in the same way at that stage. | Milk allergy assessed by food challenge by the age of 2 years | Japanese national hospital organization |
| **Introducing cashew nuts during infancy – The Cashew Study[41]**<br>**Registered Feb 12, 2018, at https://anzctr.org.au**<br>**ACTRN12618000228280**<br>**Australia** | Infants aged 6 to 8 months<br>Target No. of participants: 192 | One teaspoon vs increasing amounts (1 teaspoon from 6 to 7 months, 2 teaspoons from 8 to 9 months and 3 teaspoons from 10 months of age) of cashew nut spread three times per week until 12 months of age. | Allergy to cashew assessed by food challenge and sensitization to cashew measured by SPT and sIgE at 1 year of age | Australian Food Allergy Foundation |

© 2023 Scarpone R et al. *JAMA Pediatrics*.

| Study | Population | Intervention | Outcomes | Funding source/ sponsor |
|---|---|---|---|---|
| **Prevention study of hen's Egg Allergy with Low-allergenicity Egg powder (PEALE)[42]**<br>**Registered Nov 27, 2019, at https://jrct.niph.go.jp**<br>**jRCTs041190089**<br>**Japan** | Infants aged 4 to 5 months with atopic dermatitis and ovomucoid sIgE <2.0UA/ml<br>Target No. of participants: 200 | Heated egg powder vs ovomucoid reduced egg powder from 6 months of age | Egg allergy at 1 year of age and egg-sIgE | Kieikai Research Foundation and Japan Society for Promotion of Science Grants-in-Aid for Research |
| **Tolerance induction through early feeding to prevent food allergy in infants with eczema (TEFFA)[43]**<br>**Registered Jan 9, 2020, at https://www.drks.de**<br>**DRKS00016770**<br>**Germany** | Infants aged 4 to 8 months with eczema and with no previous consumption of egg, peanut, or hazelnut and no wheat allergy<br>Target No. of participants: 150 | Daily rusk-like biscuit powder with vs without egg, cow´s milk, peanut, and hazelnut (2mg of each food protein; the amount will be increased every 6 weeks) until 12 months of age | Food allergy to egg, cow's milk, peanut, or hazelnut in sensitized participants confirmed by an oral food challenge at around 1 year of age | Deutsche Forschungsgemeinschaft (German Research Foundation) |
| **The Effects of Complementary Feeding of Eggs on Infant Development and Growth in Guatemala: The Saqmolo Study[44]**<br>**Registered Mar 20, 2020, at https://clinicaltrials.gov**<br>**NCT04316221**<br>**Guatemala** | Infants aged 6 to 9 months with no known egg allergy, no recalcitrant, moderate-to-severe atopic dermatitis, and no history of anaphylaxis<br>Target No. of participants: 1200 | One egg per day for 6 months vs standard care | No relevant allergy outcomes specified in clinical trial registry record | Academy of Nutrition and Dietetics; Wuqu' Kawoq, Maya Health Alliance; Think Healthy Group, Inc |
| **Intervention to Reduce Early (Peanut) Allergy in Children (iREACH)[45]**<br>**Registered Oct 27, 2020, at https://clinicaltrials.gov**<br>**NCT04604431**<br>**USA** | Infants seen for 4- and 6-month well childcare visit<br>Target No. of participants: 10500 | Pediatric clinicians to receive the iREACH electronic health record integrated Clinical Decision Support tool together with educational modules on the 2017 National Institute of Allergy and Infectious Diseases Prevention of Peanut Allergy guidelines to assist in implementing the guidelines vs no intervention | Adherence to the Prevention of Peanut Allergy guidelines and peanut allergy incidence by age 2.5 years | Ann & Robert H Lurie Children's Hospital of Chicago; National Institute of Allergy and Infectious Diseases |

© 2023 Scarpone R et al. *JAMA Pediatrics*.

| Study | Population | Intervention | Outcomes | Funding source/ sponsor |
|---|---|---|---|---|
| **Grandi Byen: Supporting Child Growth and Development Through Integrated, Responsive Parenting, Nutrition and Hygiene[46]**<br>**Registered Mar 5, 2021, at https://clinicaltrials.gov**<br>**NCT04785352**<br>**Haiti** | Infants aged 6 to 8 months with no allergy to animal-source foods (specifically eggs, milk, or fish)<br>Target No. of participants: 600 | One egg per day for 6 months ± multicomponent intervention on responsive parenting, nutrition, and hygiene vs standard care | No relevant allergy outcomes specified in clinical trial registry record | Washington University School of Medicine; Hôpital Universitaire Justinien; Université Publique du Nord au Cap-Haïtien; Konbit Sante |
| **Can Early Introduction of Tree Nuts Prevent Tree Nut Allergy in Infants at High Risk of Tree Nut Allergy: The TreEat Study[47]**<br>**Registered Mar 17, 2021, at https://clinicaltrials.gov**<br>**NCT04801823**<br>**Australia** | Infants aged 4 to 11 months with peanut allergy and no anaphylaxis, tree nut allergy, tree nut allergy tests or tree nut consumption<br>Target No. of participants: 200 | Supervised hospital based multi-tree nut (almond, cashew, hazelnut, and walnut) oral food challenge, then home introduction of the remaining tree nuts vs standard care (gradual, sequential home introduction of 8 tree nuts) | Clinical confirmed tree nut allergy at 1.5 years | Murdoch Children's Research Institute |
| **Infancy to Toddlerhood: Early Nutrition & Tolerance (INTENT)[48]**<br>**Registered Mar 18, 2021, at https://clinicaltrials.gov**<br>**NCT04803981**<br>**USA** | Infants aged 4 to 6 months with no known allergy to any of the ingredients in the study intervention<br>Target No. of participants: 1500 | One SpoonfulONE product daily (mix-ins, puffs, or crackers containing 30mg of peanut, milk, shrimp, almond, cashew, hazelnut, pecan, pistachio, walnut, egg, cod, salmon, wheat, oat, soya, and sesame) vs standard diet | No relevant allergy outcomes specified in clinical trial registry record | Before Brands, Inc<br>Duke Clinical Research Institute |
| **Research on the effect and mechanism about early introduction of multi-allergen food on the risk of allergy**<br>**Registered Nov 24, 2021, at https://www.chictr.org.cn**<br>**ChiCTR2100053552**<br>**China** | Infants aged 4 to 6 months who have been formula fed for at least one month with no solid food introduction and no previous food allergy diagnosis<br>Target No. of participants: 60 | Multiple food protein supplement vs no intervention | No relevant allergy outcomes specified in clinical trial registry record | Nestlé Health Science China |

© 2023 Scarpone R et al. *JAMA Pediatrics*.

| Study | Population | Intervention | Outcomes | Funding source/ sponsor |
|---|---|---|---|---|
| **Randomised Controlled Trial to Test and Build Evidence Base for Providing Eggs as an Early Complementary Food to Promote Child Growth: Eggcel-growth Study[49]**<br>**Registered Dec 23, 2021, at https://clinicaltrials.gov**<br>**NCT05168085**<br>**South Africa** | Infants aged 6 to 9 months with no known allergy, intolerance, or sensitization to egg<br>No. of participants randomized: 655 | One egg per day for six months vs no intervention | No relevant allergy outcomes specified in clinical trial registry record | North-West University, South Africa |

Abbreviations: sIgE, specific immunoglobulin E; SPT, skin prick test.

© 2023 Scarpone R et al. *JAMA Pediatrics*.

## eTable 3. Summary of Findings for Earlier Introduction of Multiple Allergenic Foods

| | GRADE of evidence assessment | | | | | Summary of findings | |
|---|---|---|---|---|---|---|---|
| **Outcome** | **Risk of bias** | **Inconsistency** | **Indirectness** | **Imprecision** | **Publication bias** | **RR (95% CI)** | **GRADE of evidence** |
| **Allergy to any food[a]** | Not serious[b] | Serious[c] | Not serious[d] | Not serious[e] | Insufficient studies to undertake formal testing of publication bias | **0.49 (0.33-0.74)** | **Moderate** |
| **Withdrawal from study intervention[f]** | Serious[g] | Not serious[h] | Not serious[d] | Not serious[e] | | **2.29 (1.45-3.63)** | **Moderate** |
| **Allergic sensitization to any food[i]** | Not serious[j] | Serious[k] | Not serious[d] | Serious[l] | | **0.77 (0.54-1.10)** | **Low** |

CI, confidence interval; GRADE, Grading of Recommendations Assessment, Development, and Evaluation; RR, risk ratio.

[a]Intervention foods were egg, milk, wheat, soya, buckwheat, and peanut (1 trial; 161 participants); milk, peanut, egg, sesame, white fish, and wheat (1 trial; 1162 participants); milk and egg, or egg and peanut, or milk and peanut, or milk, egg, peanut, cashew, almond, shrimp, walnut, wheat, salmon, and hazelnut (1 trial; 133 participants); peanut, milk, wheat, and egg (1 trial; 1839 participants).

[b]Most information is from studies at low risk of bias or some concerns for one domain only.

[c]$I^2$=49%; relatively small difference in RR in one large study (Perkin et al, 2016) appears to explain heterogeneity, but the reason for the different findings in this study is not clear.

[d]Populations and interventions are similar to those which might be targeted outside of trial settings. Some studies used food powder, and some used normal foods. One study included infants with eczema.

[e]The 95% CI excludes a RR of no effect or effect sizes that are not clinically meaningful.

[f]Intervention foods were peanut, soya, almond, cashew, hazelnut, pecan, pistachio, walnut, wheat, oat, milk, egg, cod, shrimp, salmon, and sesame (1 trial; 705 participants); egg, milk, wheat, soya, buckwheat, and peanut (1 trial; 163 participants); milk, peanut, egg, sesame, white fish, and wheat (1 trial; 1303 participants); milk and egg, or egg and peanut, or milk and peanut, or milk, egg, peanut, cashew, almond, shrimp, walnut, wheat, salmon, and hazelnut (1 trial; 135 participants); peanut, milk, wheat, and egg (1 trial; 2397 participants).

[g]Most information is from studies at high risk of bias for one domain or some concerns for multiple domains. The main reason for risk of bias is that withdrawal could have been influenced by knowledge of intervention received.

[h]$I^2$=89%; heterogeneity appears to be explained by lower compliance in two largest studies (both high allergen intake for multiple allergenic foods, using normal foods rather than powders).

[i]Intervention foods were egg, milk, wheat, soya, buckwheat, and peanut (1 trial; 150 participants); milk, peanut, egg, sesame, white fish, and wheat (1 trial; 1173 participants); peanut, milk, wheat, and egg (1 trial; 1504 participants).

[j]All information is from studies at low risk of bias.

[k]$I^2$=73%; one study (Skjerven et al, 2022) which used skin prick test (SPT) ≥3mm showed reduced sensitization and two studies which used SPT >0mm and specific immunoglobulin E test showed no effect.

[l]Although the estimate indicates reduced allergic sensitization, the 95% CI includes the possibility of no effect.

© 2023 Scarpone R et al. *JAMA Pediatrics*.

## eTable 4. Summary of Findings for Earlier Introduction of Egg

| | GRADE of evidence assessment | | | | | Summary of findings | |
|---|---|---|---|---|---|---|---|
| **Outcome** | **Risk of bias** | **Inconsistency** | **Indirectness** | **Imprecision** | **Publication bias** | **RR (95% CI)** | **GRADE of evidence** |
| **Allergy to any food[a]** | Not serious[b] | Serious[c] | Very serious[d] | Not serious[e] | Insufficient studies to undertake formal testing of publication bias | **0.50 (0.34-0.75)** | **Very Low** |
| **Withdrawal from study intervention[f]** | Not serious[g] | Serious[h] | Not serious[i] | Serious[j] | Not likely[k] | **1.58 (1.12-2.22)** | **Low** |
| **Allergic sensitization to any food** | Results as for "earlier introduction of multiple allergenic foods"; no study of egg introduction without other foods, reporting this outcome, was identified. | | | | | | |
| **Allergy to egg[l]** | Not serious[b] | Not serious[m] | Not serious[i] | Not serious[e] | Insufficient studies to undertake formal testing of publication bias | **0.60 (0.46-0.77)** | **High[n]** |
| **Allergic sensitization to egg[o]** | Not serious[b] | Not serious[p] | Not serious[i] | Serious[j] | | **0.81 (0.69-0.96)** | **Moderate** |

CI, confidence interval; GRADE, Grading of Recommendations Assessment, Development, and Evaluation; RR, risk ratio.

[a]Intervention foods were egg, milk, wheat, soya, buckwheat, and peanut (1 trial; 161 participants); milk, peanut, egg, sesame, white fish, and wheat (1 trial; 1162 participants); egg, or milk and egg, or egg and peanut, or milk, egg, peanut, cashew, almond, shrimp, walnut, wheat, salmon, and hazelnut (1 trial; 133 participants); peanut, milk, wheat, and egg (1 trial; 1839 participants).

[b]Most information is from studies at low risk of bias or some concerns for one domain only.

[c]$I^2$=45%; relatively small difference in RR in one large study (Perkin et al, 2016) appears to explain heterogeneity, but the reason for the different findings in this study is not clear.

[d]Almost all the information for this analysis is derived from trials of multiple food interventions. The limited information for egg only shows 6/15 children developed food allergy, compared with 21/44 in a control group. There is therefore little meaningful information about the effect of earlier egg introduction on risk of allergy to any food.

[e]The 95% CI excludes a RR of no effect or effect sizes that are not clinically meaningful.

[f]Intervention foods were egg (8 trials; 2739 participants); peanut, soya, almond, cashew, hazelnut, pecan, pistachio, walnut, wheat, oat, milk, egg, cod, shrimp, salmon, and sesame (1 trial; 705 participants); egg, milk, wheat, soya, buckwheat, and peanut (1 trial; 163 participants); milk, peanut, egg, sesame, white fish, and wheat (1 trial; 1303 participants); egg, or milk and egg, or egg and peanut, or milk, egg, peanut, cashew, almond, shrimp, walnut, wheat, salmon, and hazelnut (1 trial; 135 participants); peanut, milk, wheat, and egg (1 trial; 2397 participants).

[g]Most information is from studies at high risk of bias, but findings appear to be similar to studies at low risk of bias or some concerns for one domain only.

[h]$I^2$=90%; heterogeneity appears to be explained by lower compliance in two large, pragmatic studies of high-dose multiple allergenic food introduction using normal foods, together with reports of allergic reactions to egg intervention in some smaller, single-food intervention trials.

[i]Populations and interventions are similar to those which might be targeted outside of trial settings. Some studies used food powder, but findings were similar to studies using normal foods. Populations varied in risk factors such as eczema at baseline, in whether and how egg allergy was excluded prior to the intervention and in the nature of the intervention.

[j]The 95% CI excludes a RR of no effect but includes effect sizes that are both trivial or uncertain and clinically meaningful.

[k]Funnel plot asymmetrical and Egger's test P=0.004; however, likely due to lower compliance in larger studies of multiple, high-dose allergenic food introduction, compared with smaller studies of single allergen (egg) introduction.

[l]Intervention foods were egg (6 trials; 1646 participants); egg, milk, wheat, soya, buckwheat, and peanut (1 trial; 161 participants); milk, peanut, egg, sesame, white fish, and wheat (1 trial; 1165 participants); peanut, milk, wheat, and egg (1 trial; 1839 participants).

© 2023 Scarpone R et al. *JAMA Pediatrics*.

[m]$I^2$=0%; mostly similar RRs and overlapping CIs. No obvious difference in outcome between single and multiple allergenic food introduction. Borderline interaction for high versus low dose egg introduction (P=0.02), with increased effect in low dose group.
[n]In one further high risk of bias trial which could not be included in meta-analysis, egg yolk was introduced before 3 weeks versus after 6 months in a cohort of 1753 infants; 9 participants in the earlier and 4 in the later introduction group developed symptoms before 4 and by 7 months, respectively, but precise outcome definition and denominators were not available.
[o]Intervention foods were egg (5 trials; 1504 participants); egg, milk, wheat, soya, buckwheat, and peanut (1 trial; 150 participants); milk, peanut, egg, sesame, white fish, and wheat (1 trial; 1167 participants); peanut, milk, wheat, and egg (1 trial; 1504 participants).
[p]$I^2$=18%; mostly similar RRs and overlapping CIs. *Post hoc* subgroup analysis showed a possible interaction for method of assessment (P=0.06): skin prick test (5 studies; 3740 participants) RR 0.72 (95% CI 0.58-0.89; $I^2$=0%); specific immunoglobulin E test (3 studies; 585 participants) RR 0.92 (95% CI, 0.80-1.04; $I^2$=43%).

© 2023 Scarpone R et al. *JAMA Pediatrics*.

## eTable 5. Summary of Findings for Earlier Introduction of Peanut

| | GRADE of evidence assessment | | | | | Summary of findings | |
|---|---|---|---|---|---|---|---|
| **Outcome** | **Risk of bias** | **Inconsistency** | **Indirectness** | **Imprecision** | **Publication bias** | **RR (95% CI)** | **GRADE of evidence** |
| **Allergy to any food[a]** | Not serious[b] | Serious[c] | Serious[d] | Serious[e] | Insufficient studies to undertake formal testing of publication bias | **0.60 (0.38-0.94)** | **Very low** |
| **Withdrawal from study intervention[f]** | Serious[g] | Serious[h] | Serious[d] | Not serious[i] | | **1.91 (1.19-3.05)** | **Very low** |
| **Allergic sensitization to any food[j]** | Not serious[b] | Serious[k] | Not serious[l] | Serious[e] | | **0.86 (0.71-1.05)** | **Low** |
| **Allergy to peanut[m]** | Not serious[b] | Not serious[n] | Not serious[l] | Not serious[i] | | **0.31 (0.19-0.51)** | **High** |
| **Allergic sensitization to peanut[o]** | Not serious[b] | Serious[p] | Not serious[l] | Serious[e] | | **0.74 (0.46-1.20)** | **Low** |

CI, confidence interval; GRADE, Grading of Recommendations Assessment, Development, and Evaluation; RR, risk ratio.

[a]Intervention foods were peanut (1 trial; 633 participants); egg, milk, wheat, soya, buckwheat, and peanut (1 trial; 161 participants); milk, peanut, egg, sesame, white fish, and wheat (1 trial; 1162 participants); peanut, or egg and peanut, or milk and peanut, or milk, egg, peanut, cashew, almond, shrimp, walnut, wheat, salmon, and hazelnut (1 trial; 132 participants); peanut, milk, wheat, and egg (1 trial; 1839 participants).

[b]Most or all information is from studies at low risk of bias or some concerns for one domain only.

[c]$I^2$=81%; heterogeneity appears to be partly (but not wholly) explained by a lack of effect in the single peanut-only intervention trial but reduced allergy in the multiple food intervention trials. This interaction was statistically significant (P=0.02).

[d]Some data contributing to this effect estimate are from trials of multiple allergenic food introduction. The single trial of peanut only shows no effect. Populations varied in risk factors such as eczema at baseline, in whether and how peanut allergy was excluded prior to the intervention and in the nature of the intervention.

[e]The 95% CI includes both a meaningful reduction and a trivial reduction or small increase in RR.

[f]Intervention foods were peanut (1 trial; 640 participants); peanut, soya, almond, cashew, hazelnut, pecan, pistachio, walnut, wheat, oat, milk, egg, cod, shrimp, salmon, and sesame (1 trial; 705 participants); egg, milk, wheat, soya, buckwheat, and peanut (1 trial; 163 participants); milk, peanut, egg, sesame, white fish, and wheat (1 trial; 1303 participants); peanut, or egg and peanut, or milk and peanut, or milk, egg, peanut, cashew, almond, shrimp, walnut, wheat, salmon, and hazelnut (1 trial; 135 participants); peanut, milk, wheat, and egg (1 trial; 2397 participants).

[g]Most information is from studies at high risk of bias for one domain or some concerns for multiple domains. The main reason for risk of bias is that withdrawal could have been influenced by knowledge of intervention received.

[h]$I^2$=91%; heterogeneity appears to be partly explained by lower compliance in two large, pragmatic studies of high-dose multiple allergenic food introduction using normal foods.

[i]The 95% CI excludes a RR of no effect or effect sizes that are not clinically meaningful.

[j]Intervention foods were peanut (1 trial; 629 participants); egg, milk, wheat, soya, buckwheat, and peanut (1 trial; 150 participants); milk, peanut, egg, sesame, white fish, and wheat (1 trial; 1173 participants); peanut, milk, wheat, and egg (1 trial; 1504 participants).

[k]$I^2$=61%; one study (Skjerven et al, 2022) of multiple allergenic food introduction which used skin prick test (SPT) ≥3mm showed reduced sensitization and appears to explain the heterogeneity, but the reason for different findings in this trial is unclear.

[l]Most of the events contributing to this effect estimate are from a trial of single allergenic food introduction. Populations varied in risk factors such as eczema at baseline, in whether and how peanut allergy was excluded prior to the intervention and in the nature of the intervention.

[m]Intervention foods were peanut (1 trial; 628 participants); egg, milk, wheat, soya, buckwheat, and peanut (1 trial; 161 participants); milk, peanut, egg, sesame, white fish, and wheat (1 trial; 1168 participants); peanut, milk, wheat, and egg (1 trial; 1839 participants).

[n]$I^2$=21%; mostly similar RRs and overlapping CIs.

© 2023 Scarpone R et al. *JAMA Pediatrics*.

[o]Intervention foods were peanut (1 trial; 617 participants); egg, milk, wheat, soya, buckwheat, and peanut (1 trial; 145 participants); milk, peanut, egg, sesame, white fish, and wheat (1 trial; 1168 participants); peanut, milk, wheat, and egg (1 trial; 1504 participants).

[p]$I^2$=77%; one study (Skjerven et al, 2022) of multiple allergenic food introduction which used SPT ≥3mm showed reduced sensitization and appears to explain the heterogeneity, but the reason for different findings in this trial is unclear. *Post hoc* subgroup analysis showed a possible interaction for method of assessment (P=0.06): SPT (2 studies; 2672 participants) RR 0.46 (95% CI, 0.20-1.05; $I^2$=72%); specific immunoglobulin E test (2 studies; 762 participants) RR 1.07 (95% CI, 0.85-1.34; $I^2$=0%).

© 2023 Scarpone R et al. *JAMA Pediatrics*.

## eTable 6. Summary of Findings for Earlier Introduction of Cow's Milk

| | GRADE of evidence assessment | | | | | Summary of findings | |
|---|---|---|---|---|---|---|---|
| **Outcome** | **Risk of bias** | **Inconsistency** | **Indirectness** | **Imprecision** | **Publication bias** | **RR (95% CI)** | **GRADE of evidence** |
| **Allergy to any food[a]** | Not serious[b] | Serious[c] | Serious[d] | Serious[e] | Insufficient studies to undertake formal testing of publication bias | **0.67 (0.39-1.13)** | **Very low[f]** |
| **Withdrawal from study intervention[g]** | Not serious[b] | Not serious[h] | Serious[d] | Serious[e] | Not likely[i] | **1.05 (0.61-1.82)** | **Low[j]** |
| **Allergic sensitization to any food** | Results as for "earlier introduction of multiple allergenic foods"; no study of cow's milk introduction without other foods, reporting this outcome, was identified. | | | | | | |
| **Allergy to cow's milk[k]** | Serious[l] | Not serious[m] | Serious[d] | Serious[e] | Insufficient studies to undertake formal testing of publication bias | **0.84 (0.38-1.87)** | **Very low[n]** |
| **Allergic sensitization to cow's milk[o]** | Not serious[p] | Serious[q] | Serious[d] | Serious[e] | | **1.14 (0.82-1.59)** | **Very low[r]** |

CI, confidence interval; GRADE, Grading of Recommendations Assessment, Development, and Evaluation; RR, risk ratio.

[a]Intervention foods were milk (2 trials; 686 participants); egg, milk, wheat, soya, buckwheat, and peanut (1 trial; 161 participants); milk, peanut, egg, sesame, white fish, and wheat (1 trial; 1162 participants); milk, or milk and egg, or milk and peanut, or milk, egg, peanut, cashew, almond, shrimp, walnut, wheat, salmon, and hazelnut (1 trial; 133 participants); peanut, milk, wheat, and egg (1 trial; 1839 participants).

[b]Most information is from studies at high risk of bias, but findings appear to be similar to studies at low risk of bias or some concerns for one domain only.

[c]$I^2$=83%; heterogeneity appears to be partly, but not completely, explained by one high risk of bias study.

[d]Interventions were not all representative of typical ways to introduce cow's milk products to an infant's diet. Some studies used food powder, others used formula milk compared with a control group using soya formula, but often using very early and specific exposure regimes.

[e]The 95% CI includes both a meaningful reduction and increase in risk or no effect.

[f]In one further high risk of bias study which could not be included in meta-analysis, none of 120 children who were given cow's milk (formula) and none of 115 children who were given a soya formula for the first 9 months of life developed allergy to foods such as soya, fish, orange, and nuts, and one child became clinically sensitive to chocolate after a 10-year follow-up.

[g]Intervention foods were milk (6 trials; 3192 participants); peanut, soya, almond, cashew, hazelnut, pecan, pistachio, walnut, wheat, oat, milk, egg, cod, shrimp, salmon, and sesame (1 trial; 705 participants); egg, milk, wheat, soya, buckwheat, and peanut (1 trial; 163 participants); milk, peanut, egg, sesame, white fish, and wheat (1 trial; 1303 participants); milk, or milk and egg, or milk and peanut, or milk, egg, peanut, cashew, almond, shrimp, walnut, wheat, salmon, and hazelnut (1 trial; 135 participants); peanut, milk, wheat, and egg (1 trial; 2397 participants).

[h]$I^2$=94%; heterogeneity is extreme and is fully explained by high rates of withdrawal in two pragmatic trials of multiple, high-dose, real food intervention trials, and a trial where many participants withdrew from the soya milk intervention due to a preference for cow's milk. Without these three trials included, the RR is 0.86 (95% CI, 0.70-1.06; $I^2$=0%).

[i]Funnel plot asymmetrical and Egger's test P=0.01; however, likely due to lower compliance in larger studies of multiple, high-dose allergenic food introduction, compared with smaller studies of single allergen (milk) introduction.

[j]In one further high risk of bias trial which could not be included in meta-analysis, 32/249 participants in the cow's milk group were given soya milk and 41/238 participants in the cow's milk avoidance group were given cow's milk. However, total number of participants randomized in each group was not clear as these numbers excluded some post-randomization exclusions and the number of events in the cow's milk avoidance group was inconsistent between study publications.

[k]Intervention foods were milk (3 trials; 734 participants); egg, milk, wheat, soya, buckwheat, and peanut (1 trial; 161 participants); milk, peanut, egg, sesame, white fish, and wheat (1 trial; 1166 participants); peanut, milk, wheat, and egg (1 trial; 1839 participants).

© 2023 Scarpone R et al. *JAMA Pediatrics*.

[l]Most information is from studies at high risk of bias for at least one domain and/or some concerns for multiple domains, with high heterogeneity in the high risk of bias information. The main reason for risk of bias is that outcome assessors were not blinded.
[m]$I^2$=36%; heterogeneity appears to be explained by risk of bias, with low risk of bias studies showing no heterogeneity and RR 0.32 (95% CI, 0.09-1.18).
[n]One further high risk of bias trial (Sakihara et al, 2021) reported milk allergy in 2/242 in the earlier and 17/249 in the later introduction group, assessed at 6 months, so could not be included in meta-analysis.
[o]Intervention foods were milk (4 trials; 2071 participants); egg, milk, wheat, soya, buckwheat, and peanut (1 trial; 145 participants); milk, peanut, egg, sesame, white fish, and wheat (1 trial; 1167 participants); peanut, milk, wheat, and egg (1 trial; 1504 participants).
[p]Most information is from studies at low risk of bias or some concerns for one domain only.
[q]$I^2$=45%; heterogeneity may be partly explained by method of outcome measurement. *Post hoc* subgroup analysis showed a possible interaction for method of assessment (P=0.08): skin prick test (3 studies; 2974 participants) RR 0.64 (95% CI, 0.32-1.29; $I^2$=0%); specific immunoglobulin E test (4 studies; 1913 participants) RR 1.29 (95% CI, 0.89-1.89; $I^2$=62%).
[r]One further high risk of bias trial (Sakihara et al, 2021) reported milk sensitization in 11/227 in the earlier and 38/235 in the later introduction group, assessed at 6 months, so could not be included in meta-analysis.

© 2023 Scarpone R et al. *JAMA Pediatrics*.

## eTable 7. Subgroup Analyses for Earlier Introduction of Multiple Allergenic Foods

| Outcome | Subgroup[a] | No. of participants (studies) | RR (95% CI) | $I^2$ (%) | P value for between groups difference |
|---|---|---|---|---|---|
| **Allergy to any food** | Single food | - | - | - | - |
| | Multiple foods | 3295 (4) | 0.49 (0.33-0.74) | 49 | |
| | High allergen | 3001 (2) | 0.60 (0.32-1.14) | 63 | 0.32 |
| | Low allergen | 161 (1) | 0.35 (0.16-0.80) | - | |
| | Breastmilk | 1162 (1) | 0.80 (0.51-1.25) | - | 0.02 |
| | Other | 2133 (3) | 0.40 (0.27-0.57) | 0 | |
| | High risk | 161 (1) | 0.35 (0.16-0.80) | - | 0.41 |
| | Standard/ low risk | 3134 (3) | 0.53 (0.33-0.85) | 59 | |
| **Withdrawal from study intervention** | Single food | - | - | - | - |
| | Multiple foods | 4703 (5) | 2.06 (1.00-4.26) | 89 | |
| | High allergen | 3700 (2) | 3.45 (3.13-3.80) | 0 | <0.001 |
| | Low allergen | 868 (2) | 1.02 (0.69-1.50) | 0 | |
| | Breastmilk | 1303 (1) | 3.69 (3.10-4.38) | - | 0.08 |
| | Other | 3400 (4) | 1.57 (0.62-3.98) | 91 | |
| | High risk | 163 (1) | 0.96 (0.06-15.15) | - | 0.58 |
| | Standard/ low risk | 4540 (4) | 2.15 (1.00-4.63) | 92 | |
| **Allergic sensitization to any food** | Single food | - | - | - | - |
| | Multiple foods | 2827 (3) | 0.75 (0.49-1.15) | 73 | |
| | High allergen | 2677 (2) | 0.63 (0.31-1.29) | 79 | 0.29 |
| | Low allergen | 150 (1) | 0.93 (0.83-1.04) | - | |
| | Breastmilk | 1173 (1) | 0.88 (0.62-1.25) | - | 0.50 |
| | Other | 1654 (2) | 0.66 (0.30-1.42) | 87 | |
| | High risk | 150 (1) | 0.93 (0.83-1.04) | - | 0.29 |
| | Standard/ low risk | 2677 (2) | 0.63 (0.31-1.29) | 79 | |

CI, confidence interval; RR, risk ratio.
[a]Prespecified subgroup analyses for single vs multiple allergenic food introduction; high vs low allergen intake, using a cut-off of 2g per week of the relevant individual food protein(s); milk feeding status at enrolment – exclusively breastmilk, with or without non-milk foods, vs mixed breastmilk/other milk or non-breastmilk fed; and a *post hoc* subgroup analysis of high vs standard/low risk for atopic disease based on family or personal history. Subgroup analyses were conducted using study-level variables; one small study (Quake et al, 2022) which used a mix of high and low allergen interventions within the study could not be included in the high vs low allergen intake subgroup analysis.

© 2023 Scarpone R et al. *JAMA Pediatrics*.

## eTable 8. Subgroup Analyses for Earlier Introduction of Egg

| Outcome | Subgroup[a] | No. of participants (studies) | RR (95% CI) | $I^2$ (%) | P value for between groups difference |
|---|---|---|---|---|---|
| **Allergy to any food** | Single food | - | - | - | - |
| | Multiple foods | 3162 (3) | 0.53 (0.31-0.90) | 55 | |
| | High allergen | 3001 (2) | 0.60 (0.32-1.14) | 63 | 0.32 |
| | Low allergen | 161 (1) | 0.35 (0.16-0.80) | - | |
| | Breastmilk | 1162 (1) | 0.80 (0.51-1.25) | - | 0.02 |
| | Other | 2133 (3) | 0.41 (0.28-0.58) | 0 | |
| | High risk | 161 (1) | 0.35 (0.16-0.80) | - | 0.38 |
| | Standard/ low risk | 3134 (3) | 0.54 (0.34-0.84) | 55 | |
| **Withdrawal from study intervention** | Single food | 2739 (8) | 1.39 (1.16-1.65) | 35 | 0.21 |
| | Multiple foods | 4568 (4) | 2.25 (1.07-4.71) | 91 | |
| | High allergen | 6292 (9) | 1.77 (1.20-2.61) | 90 | 0.04 |
| | Low allergen | 1015 (3) | 1.03 (0.74-1.45) | 0 | |
| | Breastmilk | 1303 (1) | 3.69 (3.10-4.38) | - | <0.001 |
| | Other | 6139 (12) | 1.45 (1.05-1.99) | 89 | |
| | High risk | 1535 (5) | 1.48 (1.10-2.01) | 18 | 0.92 |
| | Standard/ low risk | 5907 (8) | 1.53 (0.92-2.53) | 92 | |
| **Allergic sensitization to any food** | Single food | - | - | - | - |
| | Multiple foods | 2827 (3) | 0.75 (0.49-1.15) | 73 | |
| | High allergen | 2677 (2) | 0.63 (0.31-1.29) | 79 | 0.29 |
| | Low allergen | 150 (1) | 0.93 (0.83-1.04) | - | |
| | Breastmilk | 1173 (1) | 0.88 (0.62-1.25) | - | 0.50 |
| | Other | 1654 (2) | 0.66 (0.30-1.42) | 87 | |
| | High risk | 150 (1) | 0.93 (0.83-1.04) | - | 0.29 |
| | Standard/ low risk | 2677 (2) | 0.63 (0.31-1.29) | 79 | |
| **Allergy to egg** | Single food | 1646 (6) | 0.58 (0.42-0.81) | 20 | 0.86 |
| | Multiple foods | 3165 (3) | 0.61 (0.40-0.95) | 0 | |
| | High allergen | 4529 (7) | 0.68 (0.52-0.88) | 0 | 0.02 |
| | Low allergen | 282 (2) | 0.28 (0.14-0.54) | 0 | |
| | Breastmilk | 1165 (1) | 0.69 (0.40-1.18) | - | 0.56 |
| | Other | 3646 (8) | 0.57 (0.43-0.76) | 2 | |
| | High risk | 1361 (5) | 0.53 (0.37-0.75) | 30 | 0.29 |
| | Standard/ low risk | 3450 (4) | 0.73 (0.45-1.17) | 0 | |

© 2023 Scarpone R et al. *JAMA Pediatrics*.

| Outcome | Subgroup[a] | No. of participants (studies) | RR (95% CI) | $I^2$ (%) | P value for between groups difference |
|---|---|---|---|---|---|
| **Allergic sensitization to egg** | Single food | 1504 (5) | 0.70 (0.55-0.89) | 19 | 0.06 |
| | Multiple foods | 2821 (3) | 0.91 (0.80-1.03) | 0 | |
| | High allergen | 4175 (7) | 0.73 (0.59-0.90) | 0 | 0.08 |
| | Low allergen | 150 (1) | 0.91 (0.80-1.04) | - | |
| | Breastmilk | 1167 (1) | 0.83 (0.52-1.33) | - | 0.90 |
| | Other | 3158 (7) | 0.80 (0.66-0.97) | 30 | |
| | High risk | 1219 (4) | 0.78 (0.63-0.97) | 40 | 0.68 |
| | Standard/ low risk | 3106 (4) | 0.85 (0.59-1.24) | 16 | |

CI, confidence interval; RR, risk ratio.

[a]Prespecified subgroup analyses for single vs multiple allergenic food introduction; high vs low allergen intake, using a cut-off of 2g per week of the relevant individual food protein(s); milk feeding status at enrolment – exclusively breastmilk, with or without non-milk foods, vs mixed breastmilk/other milk or non-breastmilk fed; and a *post hoc* subgroup analysis of high vs standard/low risk for atopic disease based on family or personal history. Subgroup analyses were conducted using study-level variables; one small study (Quake et al, 2022) which used a mix of single and multiple allergenic food and high and low allergen interventions within the study could not be included in the single vs multiple allergenic food introduction and high vs low allergen intake subgroup analyses.

© 2023 Scarpone R et al. *JAMA Pediatrics*.

## eTable 9. Subgroup Analyses for Earlier Introduction of Peanut

| Outcome | Subgroup[a] | No. of participants (studies) | RR (95% CI) | $I^2$ (%) | P value for between groups difference |
|---|---|---|---|---|---|
| **Allergy to any food** | Single food | 633 (1) | 1.01 (0.88-1.16) | - | 0.02 |
| | Multiple foods | 3162 (3) | 0.53 (0.31-0.90) | 55 | |
| | High allergen | 3634 (3) | 0.75 (0.47-1.21) | 74 | 0.12 |
| | Low allergen | 161 (1) | 0.35 (0.16-0.80) | - | |
| | Breastmilk | 1162 (1) | 0.80 (0.51-1.25) | - | 0.29 |
| | Other | 2765 (4) | 0.55 (0.33-0.93) | 85 | |
| | High risk | 794 (2) | 0.65 (0.24-1.78) | 84 | 0.77 |
| | Standard/ low risk | 3133 (3) | 0.55 (0.36-0.84) | 50 | |
| **Withdrawal from study intervention** | Single food | 640 (1) | 0.97 (0.57-1.64) | - | 0.07 |
| | Multiple foods | 4568 (4) | 2.25 (1.07-4.71) | 92 | |
| | High allergen | 4340 (3) | 2.37 (1.06-5.31) | 91 | 0.06 |
| | Low allergen | 868 (2) | 1.02 (0.69-1.50) | 0 | |
| | Breastmilk | 1303 (1) | 3.69 (3.10-4.38) | - | 0.01 |
| | Other | 4040 (5) | 1.46 (0.72-2.94) | 92 | |
| | High risk | 803 (2) | 0.97 (0.58-1.62) | 0 | 0.07 |
| | Standard/ low risk | 4540 (4) | 2.23 (1.07-4.62) | 92 | |
| **Allergic sensitization to any food** | Single food | 629 (1) | 0.96 (0.79-1.15) | - | 0.31 |
| | Multiple foods | 2827 (3) | 0.75 (0.49-1.15) | 73 | |
| | High allergen | 3306 (3) | 0.75 (0.48-1.18) | 73 | 0.37 |
| | Low allergen | 150 (1) | 0.93 (0.83-1.04) | - | |
| | Breastmilk | 1173 (1) | 0.88 (0.62-1.25) | - | 0.67 |
| | Other | 2283 (3) | 0.78 (0.50-1.20) | 74 | |
| | High risk | 779 (2) | 0.94 (0.85-1.03) | 0 | 0.28 |
| | Standard/ low risk | 2677 (2) | 0.63 (0.31-1.29) | 79 | |
| **Allergy to peanut** | Single food | 628 (1) | 0.19 (0.10-0.36) | - | 0.06 |
| | Multiple foods | 3168 (3) | 0.43 (0.24-0.76) | 0 | |
| | High allergen | 3635 (3) | 0.31 (0.17-0.56) | 45 | 0.73 |
| | Low allergen | 161 (1) | 0.48 (0.04-5.21) | - | |
| | Breastmilk | 1168 (1) | 0.49 (0.20-1.19) | - | 0.30 |
| | Other | 2628 (3) | 0.27 (0.14-0.52) | 14 | |
| | High risk | 789 (2) | 0.20 (0.11-0.37) | 0 | 0.08 |
| | Standard/ low risk | 3007 (2) | 0.43 (0.24-0.76) | 0 | |

© 2023 Scarpone R et al. *JAMA Pediatrics*.

| Outcome | Subgroup[a] | No. of participants (studies) | RR (95% CI) | $I^2$ (%) | P value for between groups difference |
|---|---|---|---|---|---|
| **Allergic sensitization to peanut** | Single food | 617 (1) | 1.08 (0.83-1.39) | - | 0.15 |
| | Multiple foods | 2817 (3) | 0.62 (0.30-1.25) | 77 | |
| | High allergen | 3289 (3) | 0.63 (0.31-1.31) | 84 | 0.26 |
| | Low allergen | 145 (1) | 1.05 (0.65-1.69) | - | |
| | Breastmilk | 1168 (1) | 0.68 (0.40-1.15) | - | 0.89 |
| | Other | 2266 (3) | 0.73 (0.33-1.60) | 83 | |
| | High risk | 762 (2) | 1.07 (0.85-1.34) | 0 | 0.05 |
| | Standard/ low risk | 2672 (2) | 0.46 (0.20-1.05) | 72 | |

CI, confidence interval; RR, risk ratio.

[a]Prespecified subgroup analyses for single vs multiple allergenic food introduction; high vs low allergen intake, using a cut-off of 2g per week of the relevant individual food protein(s); milk feeding status at enrolment – exclusively breastmilk, with or without non-milk foods, vs mixed breastmilk/other milk or non-breastmilk fed; and a *post hoc* subgroup analysis of high vs standard/low risk for atopic disease based on family or personal history. Subgroup analyses were conducted using study-level variables; one small study (Quake et al, 2022) which used a mix of single and multiple allergenic food and high and low allergen interventions within the study could not be included in the single vs multiple allergenic food introduction and high vs low allergen intake subgroup analyses.

© 2023 Scarpone R et al. *JAMA Pediatrics*.

## eTable 10. Subgroup Analyses for Earlier Introduction of Cow's Milk

| Outcome | Subgroup[a] | No. of participants (studies) | RR (95% CI) | $I^2$ (%) | P value for between groups difference |
|---|---|---|---|---|---|
| **Allergy to any food** | Single food | 686 (2) | 1.19 (0.41-3.43) | 91 | 0.18 |
| | Multiple foods | 3162 (3) | 0.53 (0.31-0.90) | 55 | |
| | High allergen | 3687 (4) | 0.85 (0.44-1.61) | 85 | 0.10 |
| | Low allergen | 161 (1) | 0.35 (0.16-0.80) | - | |
| | Breastmilk | 1848 (3) | 1.04 (0.54-2.02) | 84 | 0.01 |
| | Other | 2133 (3) | 0.41 (0.28-0.58) | 0 | |
| | High risk | 847 (3) | 0.83 (0.31-2.22) | 89 | 0.44 |
| | Standard/ low risk | 3134 (3) | 0.54 (0.34-0.84) | 55 | |
| **Withdrawal from study intervention** | Single food | 3192 (6) | 0.78 (0.61-0.99) | 64 | 0.01 |
| | Multiple foods | 4568 (4) | 2.25 (1.07-4.71) | 92 | |
| | High allergen | 6892 (8) | 0.76 (0.24-2.33) | 95 | 0.63 |
| | Low allergen | 868 (2) | 1.02 (0.69-1.50) | 0 | |
| | Breastmilk | 3991 (6) | 0.48 (0.09-2.45) | 94 | 0.28 |
| | Other | 3904 (5) | 1.29 (0.59-2.79) | 95 | |
| | High risk | 939 (4) | 0.73 (0.38-1.38) | 0 | 0.82 |
| | Standard/ low risk | 6956 (7) | 0.85 (0.26-2.78) | 96 | |
| **Allergic sensitization to any food** | Single food | - | - | - | - |
| | Multiple foods | 2827 (3) | 0.75 (0.49-1.15) | 73 | |
| | High allergen | 2677 (2) | 0.63 (0.31-1.29) | 79 | 0.29 |
| | Low allergen | 150 (1) | 0.93 (0.83-1.04) | - | |
| | Breastmilk | 1173 (1) | 0.88 (0.62-1.25) | - | 0.50 |
| | Other | 1654 (2) | 0.66 (0.30-1.42) | 87 | |
| | High risk | 150 (1) | 0.93 (0.83-1.04) | - | 0.29 |
| | Standard/ low risk | 2677 (2) | 0.63 (0.31-1.29) | 79 | |
| **Allergy to cow's milk** | Single food | 734 (3) | 1.54 (0.37-6.39) | 61 | 0.18 |
| | Multiple foods | 3166 (3) | 0.47 (0.18-1.26) | 0 | |
| | High allergen | 3739 (5) | 0.96 (0.49-1.87) | 36 | 0.21 |
| | Low allergen | 161 (1) | 0.32 (0.07-1.54) | - | |
| | Breastmilk | 1900 (4) | 1.16 (0.49-2.75) | 43 | 0.11 |
| | Other | 2000 (2) | 0.32 (0.09-1.18) | 0 | |
| | High risk | 895 (4) | 1.04 (0.32-3.43) | 58 | 0.54 |
| | Standard/ low risk | 3005 (2) | 0.60 (0.17-2.10) | 0 | |

© 2023 Scarpone R et al. *JAMA Pediatrics*.

| Outcome | Subgroup[a] | No. of participants (studies) | RR (95% CI) | $I^2$ (%) | P value for between groups difference |
|---|---|---|---|---|---|
| **Allergic sensitization to cow's milk** | Single food | 2071 (4) | 1.44 (1.00-2.07) | 25 | 0.05 |
| | Multiple foods | 2816 (3) | 0.93 (0.72-1.19) | 0 | |
| | High allergen | 4742 (6) | 1.18 (0.75-1.86) | 33 | 0.43 |
| | Low allergen | 145 (1) | 0.96 (0.74-1.25) | - | |
| | Breastmilk | 3238 (5) | 1.18 (0.74-1.89) | 46 | 0.44 |
| | Other | 1649 (2) | 0.96 (0.74-1.25) | 0 | |
| | High risk | 782 (4) | 1.15 (0.71-1.85) | 63 | 0.81 |
| | Standard/ low risk | 4105 (3) | 1.03 (0.49-2.17) | 26 | |

CI, confidence interval; RR, risk ratio.

[a]Prespecified subgroup analyses for single vs multiple allergenic food introduction; high vs low allergen intake, using a cut-off of 2g per week of the relevant individual food protein(s); milk feeding status at enrolment – exclusively breastmilk, with or without non-milk foods, vs mixed breastmilk/other milk or non-breastmilk fed; and a *post hoc* subgroup analysis of high vs standard/low risk for atopic disease based on family or personal history. Subgroup analyses were conducted using study-level variables; one small study (Quake et al, 2022) which used a mix of single and multiple allergenic food and high and low allergen interventions within the study could not be included in the single vs multiple allergenic food introduction and high vs low allergen intake subgroup analyses.

© 2023 Scarpone R et al. *JAMA Pediatrics*.

## eTable 11. Sensitivity Analysis of Low Risk of Bias Data

| Intervention | Outcome | No. of participants (studies) | RR (95% CI) | $I^2$ (%) |
|---|---|---|---|---|
| **Earlier introduction of multiple allergenic foods** | Allergy to any food | 2000 (2) | 0.39 (0.24-0.65) | 0 |
| | Withdrawal from study intervention | 163 (1) | 0.96 (0.06-15.15) | - |
| | Allergic sensitization to any food | 2827 (3) | 0.75 (0.49-1.15) | 73 |
| **Earlier introduction of egg** | Allergy to any food | 2000 (2) | 0.39 (0.24-0.65) | 0 |
| | Withdrawal from study intervention | 1388 (4) | 1.62 (1.09-2.40) | 26 |
| | Allergic sensitization to any food | 2827 (3) | 0.75 (0.49-1.15) | 73 |
| | Allergy to egg | 2825 (4) | 0.63 (0.46-0.86) | 0 |
| | Allergic sensitization to egg | 3646 (5) | 0.85 (0.74-0.99) | 0 |
| **Earlier introduction of peanut** | Allergy to any food | 2000 (2) | 0.39 (0.24-0.65) | 0 |
| | Withdrawal from study intervention | 163 (1) | 0.96 (0.06-15.15) | - |
| | Allergic sensitization to any food | 2827 (3) | 0.75 (0.49-1.15) | 73 |
| | Allergy to peanut | 2000 (2) | 0.40 (0.19-0.82) | 0 |
| | Allergic sensitization to peanut | 2817 (3) | 0.62 (0.30-1.25) | 77 |
| **Earlier introduction of cow's milk** | Allergy to any food | 2000 (2) | 0.39 (0.24-0.65) | 0 |
| | Withdrawal from study intervention | 163 (1) | 0.96 (0.06-15.15) | - |
| | Allergic sensitization to any food | 2827 (3) | 0.75 (0.49-1.15) | 73 |
| | Allergy to cow's milk | 2000 (2) | 0.32 (0.09-1.18) | 0 |
| | Allergic sensitization to cow's milk | 3102 (4) | 1.11 (0.62-1.98) | 66 |

CI, confidence interval; RR, risk ratio.

© 2023 Scarpone R et al. *JAMA Pediatrics*.

## eTable 12. Earlier Introduction of Other Common Allergenic Foods

| Intervention | Outcome | No. of participants (studies) | Earlier introduction | Later introduction | RR (95% CI) | $I^2$ (%) |
|---|---|---|---|---|---|---|
| **Earlier introduction of wheat** | Allergy to any food | 3250 (4)[a] | 54/1617 | 113/1633 | 0.40 (0.20-0.79) | 72 |
| | Withdrawal from study intervention | 4658 (5)[b] | 1350/2371 | 409/2287 | 2.34 (1.49-3.68) | 89 |
| | Allergic sensitization to any food | Results as for "earlier introduction of multiple allergenic foods". | | | | |
| | Allergy to wheat | 3169 (3)[c] | 1/1578 | 3/1591 | 0.66 (0.10-4.47) | 2 |
| | Allergic sensitization to wheat | 2818 (3)[d] | 46/1409 | 60/1409 | 0.62 (0.29-1.34) | 59 |
| **Earlier introduction of soya** | Allergy to any food | 545 (2)[e] | 44/273 | 45/272 | 0.74 (0.19-2.93) | 88 |
| | Withdrawal from study intervention | 2215 (6)[f] | 197/1116 | 93/1099 | 1.43 (0.80-2.54) | 63 |
| | Allergic sensitization to any food | Only one study of soya introduction (with other foods)[g], reporting this outcome, was identified (150 participants); allergic sensitization to any food developed in 65/76 vs 68/74 participants in the earlier vs later introduction group. | | | | |
| | Allergy to soya | Only one study of soya introduction (with other foods)[g], reporting this outcome, was identified (161 participants); allergy to soya developed in 1/82 vs 0/79 participants in the earlier vs later introduction group. | | | | |
| | Allergic sensitization to soya | 192 (2)[h] | 37/97 | 30/95 | 1.14 (0.79-1.65) | 0 |
| **Earlier introduction of fish** | Allergy to any food | 1250 (2)[i] | 34/611 | 63/639 | 0.31 (0.04-2.44) | 88 |
| | Withdrawal from study intervention | 2098 (3)[j] | 478/1063 | 159/1035 | 1.80 (0.56-5.74) | 94 |
| | Allergic sensitization to any food | Only one study of fish introduction (with other foods)[k], reporting this outcome, was identified (1173 participants); allergic sensitization to any food developed in 51/572 vs 61/601 participants in the earlier vs later introduction group. | | | | |
| | Allergy to fish | Only one study of fish introduction (with other foods)[k], reporting this outcome, was identified (1174 participants); allergy to fish developed in 1/573 vs 1/601 participants in the earlier vs later introduction group. | | | | |
| | Allergic sensitization to fish | Only one study of fish introduction (with other foods)[k], reporting this outcome, was identified (1166 participants); allergic sensitization to fish developed in 4/567 vs 5/599 participants in the earlier vs later introduction group. | | | | |

© 2023 Scarpone R et al. *JAMA Pediatrics*.

| Intervention | Outcome | No. of participants (studies) | Earlier introduction | Later introduction | RR (95% CI) | $I^2$ (%) |
|---|---|---|---|---|---|---|
| **Earlier introduction of crustaceans or tree nuts** | Allergy to any food | Only one study of introduction of crustaceans or tree nuts (with other foods)[l], reporting this outcome, was identified (88 participants); allergy to any food developed in 2/44 vs 21/44 participants in the earlier vs later introduction group. | | | | |
| | Withdrawal from study intervention | 795 (2)[m] | 46/411 | 42/384 | 1.02 (0.69-1.50) | 0 |
| | Allergic sensitization to any food | No study of introduction of crustaceans or tree nuts (with or without other foods), reporting allergic sensitization to any food, was identified. | | | | |
| | Allergy to crustaceans or tree nuts | No study of introduction of crustaceans or tree nuts (with or without other foods), reporting allergy to crustaceans or tree nuts, was identified. | | | | |
| | Allergic sensitization to crustaceans or tree nuts | No study of introduction of crustaceans or tree nuts (with or without other foods), reporting allergic sensitization to crustaceans or tree nuts, was identified. | | | | |

CI, confidence interval; RR, risk ratio.

[a]Intervention foods were egg, milk, wheat, soya, buckwheat, and peanut (1 trial; 161 participants); milk, peanut, egg, sesame, white fish, and wheat (1 trial; 1162 participants); milk, egg, peanut, cashew, almond, shrimp, walnut, wheat, salmon, and hazelnut (1 trial; 88 participants); peanut, milk, wheat, and egg (1 trial; 1839 participants).

[b]Intervention foods were peanut, soya, almond, cashew, hazelnut, pecan, pistachio, walnut, wheat, oat, milk, egg, cod, shrimp, salmon, and sesame (1 trial; 705 participants); egg, milk, wheat, soya, buckwheat, and peanut (1 trial; 163 participants); milk, peanut, egg, sesame, white fish, and wheat (1 trial; 1303 participants); milk, egg, peanut, cashew, almond, shrimp, walnut, wheat, salmon, and hazelnut (1 trial; 90 participants); peanut, milk, wheat, and egg (1 trial; 2397 participants).

[c]Intervention foods were egg, milk, wheat, soya, buckwheat, and peanut (1 trial; 161 participants); milk, peanut, egg, sesame, white fish, and wheat (1 trial; 1169 participants); peanut, milk, wheat, and egg (1 trial; 1839 participants).

[d]Intervention foods were egg, milk, wheat, soya, buckwheat, and peanut (1 trial; 146 participants); milk, peanut, egg, sesame, white fish, and wheat (1 trial; 1168 participants); peanut, milk, wheat, and egg (1 trial; 1504 participants).

[e]Intervention foods were soya (1 trial; 384 participants); egg, milk, wheat, soya, buckwheat, and peanut (1 trial; 161 participants).

[f]Intervention foods were soya (4 trials; 1347 participants); peanut, soya, almond, cashew, hazelnut, pecan, pistachio, walnut, wheat, oat, milk, egg, cod, shrimp, salmon, and sesame (1 trial; 705 participants); egg, milk, wheat, soya, buckwheat, and peanut (1 trial; 163 participants).

[g]Intervention foods were egg, milk, wheat, soya, buckwheat, and peanut.

[h]Intervention foods were soya (1 trial; 48 participants); egg, milk, wheat, soya, buckwheat, and peanut (1 trial; 144 participants).

[i]Intervention foods were milk, peanut, egg, sesame, white fish, and wheat (1 trial; 1162 participants); milk, egg, peanut, cashew, almond, shrimp, walnut, wheat, salmon, and hazelnut (1 trial; 88 participants).

[j]Intervention foods were peanut, soya, almond, cashew, hazelnut, pecan, pistachio, walnut, wheat, oat, milk, egg, cod, shrimp, salmon, and sesame (1 trial; 705 participants); milk, peanut, egg, sesame, white fish, and wheat (1 trial; 1303 participants); milk, egg, peanut, cashew, almond, shrimp, walnut, wheat, salmon, and hazelnut (1 trial; 90 participants).

[k]Intervention foods were milk, peanut, egg, sesame, white fish, and wheat.

[l]Intervention foods were milk, egg, peanut, cashew, almond, shrimp, walnut, wheat, salmon, and hazelnut.

[m]Intervention foods were peanut, soya, almond, cashew, hazelnut, pecan, pistachio, walnut, wheat, oat, milk, egg, cod, shrimp, salmon, and sesame (1 trial; 705 participants); milk, egg, peanut, cashew, almond, shrimp, walnut, wheat, salmon, and hazelnut (1 trial; 90 participants).

© 2023 Scarpone R et al. *JAMA Pediatrics*.

## eReferences


1. Ierodiakonou D, Garcia-Larsen V, Logan A, et al. Timing of Allergenic Food Introduction to the Infant Diet and Risk of Allergic or Autoimmune Disease: A Systematic Review and Meta-analysis. *JAMA*. Sep 20 2016;316(11):1181-1192. doi:10.1001/jama.2016.12623
2. Boyle RJ, Ierodiakonou D, Khan T, et al. Hydrolysed formula and risk of allergic or autoimmune disease: systematic review and meta-analysis. *Bmj*. Mar 8 2016;352:i974. doi:10.1136/bmj.i974
3. Garcia-Larsen V, Ierodiakonou D, Jarrold K, et al. Diet during pregnancy and infancy and risk of allergic or autoimmune disease: A systematic review and meta-analysis. *PLoS Med*. Feb 2018;15(2):e1002507. doi:10.1371/journal.pmed.1002507
4. Egger M, Davey Smith G, Schneider M, Minder C. Bias in meta-analysis detected by a simple, graphical test. *Bmj*. Sep 13 1997;315(7109):629-34. doi:10.1136/bmj.315.7109.629
5. Sterne JAC, Savovic J, Page MJ, et al. RoB 2: a revised tool for assessing risk of bias in randomised trials. *Bmj*. Aug 28 2019;366:l4898. doi:10.1136/bmj.l4898
6. R Core Team (2022). R: A language and environment for statistical computing. R Foundation for Statistical Computing, Vienna, Austria. URL https://www.R-project.org/.
7. Balduzzi S, Rucker G, Schwarzer G. How to perform a meta-analysis with R: a practical tutorial. *Evid Based Ment Health*. Nov 2019;22(4):153-160. doi:10.1136/ebmental-2019-300117
8. Viechtbauer W. Conducting Meta-Analyses in R with the metafor Package. *Journal of Statistical Software*. 08/05 2010;36(3):1 - 48. doi:10.18637/jss.v036.i03
9. Mantel N, Haenszel W. Statistical aspects of the analysis of data from retrospective studies of disease. *J Natl Cancer Inst*. Apr 1959;22(4):719-48.
10. DerSimonian R, Laird N. Meta-analysis in clinical trials. *Control Clin Trials*. Sep 1986;7(3):177-88. doi:10.1016/0197-2456(86)90046-2
11. Trial Sequential Analysis (TSA) [Computer program]. Version 0.9.5.10 Beta. The Copenhagen Trial Unit, Centre for Clinical Intervention Research, The Capital Region, Copenhagen University Hospital – Rigshospitalet, 2021.
12. Guyatt GH, Oxman AD, Vist GE, et al. GRADE: an emerging consensus on rating quality of evidence and strength of recommendations. *Bmj*. Apr 26 2008;336(7650):924-6. doi:10.1136/bmj.39489.470347.AD
13. Guyatt G, Oxman AD, Akl EA, et al. GRADE guidelines: 1. Introduction-GRADE evidence profiles and summary of findings tables. *J Clin Epidemiol*. Apr 2011;64(4):383-94. doi:10.1016/j.jclinepi.2010.04.026
14. Bellach J, Schwarz V, Ahrens B, et al. Randomized placebo-controlled trial of hen's egg consumption for primary prevention in infants. *Journal of allergy and clinical immunology*. 2017;139(5):1591-1599.e2. doi:10.1016/j.jaci.2016.06.045
15. Brown EB, Josephson BM, Levine HS, Rosen M. A prospective study of allergy in a pediatric population. The role of heredity in the incidence of allergies, and experience with milk-free diet in the newborn. *Am J Dis Child*. Jun 1969;117(6):693-8.
16. de Jong MH, Scharp-van der Linden VT, Aalberse RC, Oosting J, Tijssen JG, de Groot CJ. Randomised controlled trial of brief neonatal exposure to cows' milk on the development of atopy. *Archives of disease in childhood*. 1998;79(2):126-130. doi:10.1136/adc.79.2.126
17. Du Toit G, Roberts G, Sayre PH, et al. Randomized trial of peanut consumption in infants at risk for peanut allergy. *N Engl J Med*. 2015;372(9):803-13. doi:https://dx.doi.org/10.1056/NEJMoa1414850
18. du Toit G, Sayre PH, Roberts G, et al. Allergen specificity of early peanut consumption and effect on development of allergic disease in the Learning Early About Peanut Allergy study cohort. *Journal of allergy and clinical immunology*. 2018;141(4):1343-1353. doi:10.1016/j.jaci.2017.09.034
19. Halpern SR, Sellars WA, Johnson RB, Anderson DW, Saperstein S, Reisch JS. Development of childhood allergy in infants fed breast, soy, or cow milk. *J Allergy Clin Immunol*. 1973;51(3):139-51.
20. Holl JL, Bilaver LA, Finn DJ, Savio K. A randomized trial of the acceptability of a daily multi-allergen food supplement for infants. *Pediatric allergy and immunology*. 2020;31(4):418-420. doi:10.1111/pai.13223
21. Iannotti LL, Lutter CK, Stewart CP, et al. Eggs in Early Complementary Feeding and Child Growth: a Randomized Controlled Trial. *Pediatrics*. 2017;140(1)doi:10.1542/peds.2016-3459
22. Johnstone DE, Dutton AM. Dietary prophylaxis of allergic disease in children. *N Engl J Med*. 1966;274(13):715-9.
23. Kjellman NI, Johansson SG. Soy versus cow's milk in infants with a biparental history of atopic disease: development of atopic disease and immunoglobulins from birth to 4 years of age. *Clinical allergy*. 1979;9(4):347-358. doi:10.1111/j.1365-2222.1979.tb02493.x
24. Lowe AJ, Hosking CS, Bennett CM, et al. Effect of a partially hydrolyzed whey infant formula at weaning on risk of allergic disease in high-risk children: a randomized controlled trial. *Journal of allergy and clinical immunology*. 2011;128(2):360-365.e4. doi:10.1016/j.jaci.2010.05.006


© 2023 Scarpone R et al. *JAMA Pediatrics*.

25. Makrides M, Hawkes JS, Neumann MA, Gibson RA. Nutritional effect of including egg yolk in the weaning diet of breast-fed and formula-fed infants: a randomized controlled trial. *Am J Clin Nutr*. Jun 2002;75(6):1084-92. doi:10.1093/ajcn/75.6.1084
26. Miskelly FG, Burr ML, Vaughan-Williams E, Fehily AM, Butland BK, Merret TG. Infant feeding and allergy. *Archives of Disease in Childhood*. 1988;63(4):388-393. doi:http://dx.doi.org/10.1136/adc.63.4.388
27. Hand S, Dunstan F, Jones K, Doull I. The effect of diet in infancy on asthma in young adults: the Merthyr Allergy Prevention Study. *Thorax*. 2021;07:07. doi:https://dx.doi.org/10.1136/thoraxjnl-2020-215040
28. Natsume O, Kabashima S, Nakazato J, et al. Two-step egg introduction for prevention of egg allergy in high-risk infants with eczema (PETIT): a randomised, double-blind, placebo-controlled trial. *Lancet (london, england)*. 2017;389(10066):276-286. doi:10.1016/S0140-6736(16)31418-0
29. Nishimura T, Fukazawa M, Fukuoka K, et al. Early introduction of very small amounts of multiple foods to infants: a randomized trial. *Allergology international*. 2022;doi:10.1016/j.alit.2022.03.001
30. Palmer DJ, Metcalfe J, Makrides M, et al. Early regular egg exposure in infants with eczema: a randomized controlled trial. *Journal of allergy and clinical immunology*. 2013;132(2):387-92.e1. doi:10.1016/j.jaci.2013.05.002
31. Palmer DJ, Sullivan TR, Gold MS, Prescott SL, Makrides M. Randomized controlled trial of early regular egg intake to prevent egg allergy. *Journal of allergy and clinical immunology*. 2017;139(5):1600-1607.e2. doi:10.1016/j.jaci.2016.06.052
32. Perkin MR, Logan K, Tseng A, et al. Randomized Trial of Introduction of Allergenic Foods in Breast-Fed Infants. *N Engl J Med*. 2016;374(18):1733-43. doi:https://dx.doi.org/10.1056/NEJMoa1514210
33. Quake AZ, Liu TA, D'Souza R, et al. Early Introduction of Multi-Allergen Mixture for Prevention of Food Allergy: Pilot Study. *Nutrients*. 2022;14(4) (no pagination)doi:https://dx.doi.org/10.3390/nu14040737
34. Sakihara T, Otsuji K, Arakaki Y, Hamada K, Sugiura S, Ito K. Randomized trial of early infant formula introduction to prevent cow's milk allergy. *Journal of allergy and clinical immunology*. 2021;147(1):224-232.e8. doi:10.1016/j.jaci.2020.08.021
35. Skjerven HO, Lie A, Vettukattil R, et al. Early food intervention and skin emollients to prevent food allergy in young children (PreventADALL): a factorial, multicentre, cluster-randomised trial. *Lancet*. 2022;399(10344):2398-2411. doi:https://dx.doi.org/10.1016/S0140-6736(22)00687-0
36. Stewart CP, Caswell B, Iannotti L, et al. The effect of eggs on early child growth in rural Malawi: the Mazira Project randomized controlled trial. *Am J Clin Nutr*. Oct 1 2019;110(4):1026-1033. doi:10.1093/ajcn/nqz163
37. Wei-Liang Tan J, Valerio C, Barnes EH, et al. A randomized trial of egg introduction from 4 months of age in infants at risk for egg allergy. *Journal of allergy and clinical immunology*. 2017;139(5):1621-1628.e8. doi:10.1016/j.jaci.2016.08.035
38. Urashima M, Mezawa H, Okuyama M, et al. Primary Prevention of Cow's Milk Sensitization and Food Allergy by Avoiding Supplementation With Cow's Milk Formula at Birth: a Randomized Clinical Trial. *JAMA pediatrics*. 2019;173(12):1137-1145. doi:10.1001/jamapediatrics.2019.3544
39. Study on the Induction of Food Tolerance in Babies. *https://clinicaltrialsgov/show/NCT02825069*. 2016;
40. Randomized controlled trial of intake of milk in infancy by high-risk infants to prevent milk allergy. *http://wwwwhoint/trialsearch/Trial2aspx?TrialID=JPRN-UMIN000030214*. 2017;
41. Introducing cashew nuts during infancy – The Cashew Study. *http://wwwwhoint/trialsearch/Trial2aspx?TrialID=ACTRN12618000228280*. 2018;
42. Prevention study of hen's Egg Allergy with Low-allergenisity Egg powder. *http://wwwwhoint/trialsearch/Trial2aspx?TrialID=JPRN-jRCTs041190089*. 2019;
43. Kalb B, Meixner L, Trendelenburg V, et al. Tolerance induction through early feeding to prevent food allergy in infants with eczema (TEFFA): rationale, study design, and methods of a randomized controlled trial. *Trials*. 2022;23(1) https://dx.doi.org/10.1186/s13063-022-06126-x
44. Effects of Complementary Feeding of Eggs on Infant Development and Growth in Guatemala: the Saqmolo Study. *https://clinicaltrialsgov/show/NCT04316221*. 2020;
45. Intervention to Reduce Early (Peanut) Allergy in Children. *https://clinicaltrialsgov/show/NCT04604431*. 2020;
46. Kohl PL, Gyimah EA, Diaz J, et al. Grandi Byen-supporting child growth and development through integrated, responsive parenting, nutrition and hygiene: study protocol for a randomized controlled trial. *BMC pediatrics*. 2022;22(1)https://dx.doi.org/10.1186/s12887-021-03089-x
47. The TreEat Study- Can Early Introduction of Tree Nuts Prevent Tree Nut Allergy in Infants With Peanut Allergy. *https://clinicaltrialsgov/show/NCT04801823*. 2021;
48. Infancy to Toddlerhood: early Nutrition & Tolerance (INTENT). *https://clinicaltrialsgov/show/NCT04803981*. 2021;

© 2023 Scarpone R et al. *JAMA Pediatrics*.

49. Nct. Efficacy of Providing Eggs as an Early Complementary Food to Promote Child Growth. *https://clinicaltrialsgov/show/NCT05168085*. 2021;

© 2023 Scarpone R et al. *JAMA Pediatrics*.

## eAppendix. Search Strategies

### Embase

1. complementary food?.ab,ti.
2. (introduc$ adj2 food?).ab,ti.
3. wean$.ab,ti.
4. weaning/
5. solid?.ab,ti.
6. semi-solid?.ab,ti.
7. baby food?.ab,ti.
8. baby food/
9. infant nutrition/
10. breast feeding.ab,ti.
11. breastfeeding.ab,ti.
12. breast fed.ab,ti.
13. breastfed.ab,ti.
14. breast feeding/
15. breast milk/
16. formula?.ab,ti.
17. hydrolysed.ab,ti.
18. bottlefed.ab,ti.
19. bottle fed.ab,ti.
20. (bottle adj3 feed$).ab,ti.
21. artificial milk/
22. bottle feeding/
23. liquid?.ti,ab.
24. milk.ti,ab.
25. milk/
26. egg?.ti,ab.
27. egg/
28. egg protein/
29. nut?.ab,ti.
30. peanut?.ab,ti.
31. almond?.ab,ti.
32. (brazil? adj5 nut?).ab,ti.
33. walnut?.ab,ti.
34. pecan?.ab,ti.
35. pistachio?.ab,ti.
36. cashew?.ab,ti.
37. hazelnut?.ab,ti.
38. macadamia?.ab,ti.
39. nut/
40. peanut/
41. almond/
42. Brazil nut/
43. exp walnut/
44. pecan/
45. pistachio/
46. cashew nut/
47. hazelnut/
48. Corylus avellana/
49. Macadamia/
50. wheat.ti,ab.
51. exp wheat/
52. soya.ti,ab.
53. soybean/
54. gluten$.ti,ab.
55. gluten/
56. fish$.ti,ab.
57. fish/

© 2023 Scarpone R et al. *JAMA Pediatrics*.

58. 1 or 2 or 3 or 4 or 5 or 6 or 7 or 8 or 9 or 10 or 11 or 12 or 13 or 14 or 15 or 16 or 17 or 18 or 19 or 20 or 21 or 22 or 23 or 24 or 25 or 26 or 27 or 28 or 29 or 30 or 31 or 32 or 33 or 34 or 35 or 36 or 37 or 38 or 39 or 40 or 41 or 42 or 43 or 44 or 45 or 46 or 47 or 48 or 49 or 50 or 51 or 52 or 53 or 54 or 55 or 56 or 57
59. allerg$.ab,ti.
60. asthma$.ab,ti.
61. wheeze.ab,ti.
62. wheezing.ab,ti.
63. bronchial hyperresponsiveness.ab,ti.
64. bronchial hyperreactivity.ab,ti.
65. Forced expiratory volume.ab,ti.
66. FEV1.ab,ti.
67. "FEV 1".ab,ti.
68. "FEV0.5".ab,ti.
69. "FEV 0.5".ab,ti.
70. Forced vital capacity.ab,ti.
71. FVC.ab,ti.
72. Peak expiratory flow rate.ab,ti.
73. PEFR.ab,ti.
74. eczema.ab,ti.
75. neurodermatitis.ab,ti.
76. rhinitis.ab,ti.
77. besniers prurigo.ab,ti.
78. rhinoconjunctivitis.ab,ti.
79. hayfever.ab,ti.
80. (hay adj fever).ab,ti.
81. poll?nosis.ab,ti.
82. SAR.ab,ti.
83. (pollen adj allergy).ab,ti.
84. conjunctivitis.ab,ti.
85. immunoglobulin e.ab,ti.
86. Total IgE.ab,ti.
87. atopic disease.ab,ti.
88. atopic dermatitis.ab,ti.
89. (food? adj3 sensiti$).ab,ti.
90. (food? adj3 toleran$).ab,ti.
91. (food? adj3 intoleran$).ab,ti.
92. ((aero or air$) adj3 allergen?).ab,ti.
93. (aeroallergen? adj3 sensiti$).ab,ti.
94. (allergen? adj3 sensiti$).ab,ti.
95. skin prick test$.ab,ti.
96. atopy.ab,ti.
97. hypersensitiv$.ab,ti.
98. exp hypersensitivity/
99. respiratory tract allergy/
100. asthma/
101. wheezing/
102. bronchus hyperreactivity/
103. forced expiratory volume/
104. forced vital capacity/
105. peak expiratory flow/
106. eczema/
107. neurodermatitis/
108. rhinitis/
109. rhinoconjunctivitis/
110. hay fever/
111. pollen allergy/
112. perennial rhinitis/
113. conjunctivitis/
114. immunoglobulin E/
115. atopic dermatitis/

© 2023 Scarpone R et al. *JAMA Pediatrics*.

116. nutritional intolerance/
117. 59 or 60 or 61 or 62 or 63 or 64 or 65 or 66 or 67 or 68 or 69 or 70 or 71 or 72 or 73 or 74 or 75 or 76 or 77 or 78 or 79 or 80 or 81 or 82 or 83 or 84 or 85 or 86 or 87 or 88 or 89 or 90 or 91 or 92 or 93 or 94 or 95 or 96 or 97 or 98 or 99 or 100 or 101 or 102 or 103 or 104 or 105 or 106 or 107 or 108 or 109 or 110 or 111 or 112 or 113 or 114 or 115 or 116
118. infant?.ab,ti.
119. ((one or two or three or four or five or six or seven or eight or nine or ten or eleven or twelve or thirteen or fourteen or fifteen or sixteen or seventeen or eighteen or nineteen or twenty or "twenty one" or "twenty two" or "twenty three" or "twenty four" or "twenty five" or "twenty six") adj week?).ab,ti.
120. ((one or two or three or four or five or six or seven or eight or nine or ten or eleven or twelve or thirteen or fourteen or fifteen or sixteen or seventeen or eighteen or nineteen or twenty or "twenty one" or "twenty two" or "twenty three" or "twenty four") adj month?).ab,ti.
121. ((1 or 2 or 3 or 4 or 5 or 6 or 7 or 8 or 9 or 10 or 11 or 12 or 13 or 14 or 15 or 16 or 17 or 18 or 19 or 20 or 21 or 22 or 23 or 24 or 25 or 26) adj week?).ab,ti.
122. ((1 or 2 or 3 or 4 or 5 or 6 or 7 or 8 or 9 or 10 or 11 or 12 or 13 or 14 or 15 or 16 or 17 or 18 or 19 or 20 or 21 or 22 or 23 or 24) adj month?).ab,ti.
123. 119 or 120 or 121 or 122
124. (old or age?).ab,ti.
125. 123 and 124
126. (("one year?" or "two year?") adj3 (old or age?)).ab,ti.
127. ((first or second or two) adj3 "year? of life").ab,ti.
128. (("1 year?" or "2 year?") adj3 (old or age?)).ab,ti.
129. ((first or second or 2) adj3 "year? of life").ab,ti.
130. infant/
131. newborn/
132. 118 or 125 or 126 or 127 or 128 or 129 or 130 or 131
133. clinical trial?.mp.
134. random$.mp.
135. factorial$.mp.
136. crossover$.mp.
137. placebo$.mp.
138. (doubl$ adj blind$).mp.
139. (singl$ adj blind$).mp.
140. assign$.mp.
141. volunteer$.mp.
142. longitudinal$.mp.
143. follow-up.mp.
144. groups.ab,ti.
145. exp clinical trial/
146. crossover procedure/
147. placebo/
148. double blind procedure/
149. single blind procedure/
150. follow up/
151. 133 or 134 or 135 or 136 or 137 or 138 or 139 or 140 or 141 or 142 or 143 or 144 or 145 or 146 or 147 or 148 or 149 or 150
152. 58 and 117 and 132 and 151

© 2023 Scarpone R et al. *JAMA Pediatrics.*

**CENTRAL**

1. “complementary food*”:ab,ti
2. (introduc* NEAR/2 food*):ab,ti
3. wean*:ab,ti
4. MeSH descriptor [Weaning] this term only
5. solid*:ab,ti
6. semi-solid*:ab,ti
7. “baby food*”:ab,ti
8. MeSH descriptor [Infant Food] this term only
9. MeSH descriptor [Infant Nutritional Physiological Phenomena] this term only
10. “breast feeding”:ab,ti
11. breastfeeding:ab,ti
12. “breast fed”:ab,ti
13. breastfed:ab,ti
14. MeSH descriptor [Breast Feeding] this term only
15. MeSH descriptor [Milk, Human] this term only
16. formula*:ab,ti
17. hydrolysed:ab,ti
18. bottlefed:ab,ti
19. “bottle fed”:ab,ti
20. (bottle NEAR/3 feed*):ab,ti
21. MeSH descriptor [Infant Formula] this term only
22. MeSH descriptor [Bottle Feeding] this term only
23. liquid*:ab,ti
24. milk:ab,ti
25. MeSH descriptor [Milk] this term only
26. egg*:ab,ti
27. MeSH descriptor [Egg Proteins] this term only
28. MeSH descriptor [Egg Proteins, Dietary] this term only
29. nut*:ab,ti
30. peanut*:ab,ti
31. almond*:ab,ti
32. (brazil* NEAR/5 nut*):ab,ti
33. walnut*:ab,ti
34. pecan*:ab,ti
35. pistachio*:ab,ti
36. cashew*:ab,ti
37. hazelnut*:ab,ti
38. macadamia*:ab,ti
39. MeSH descriptor [Nuts] this term only
40. MeSH descriptor [Arachis hypogaea] this term only
41. MeSH descriptor [Prunus] this term only
42. MeSH descriptor [Bertholletia] this term only
43. MeSH descriptor [Juglans] this term only
44. MeSH descriptor [Carya] this term only
45. MeSH descriptor [Pistacia] this term only
46. MeSH descriptor [Anacardium] this term only
47. MeSH descriptor [Corylus] this term only
48. MeSH descriptor [Macadamia] this term only
49. wheat:ab,ti
50. MeSH descriptor [Triticum] this term only
51. soya:ab,ti
52. MeSH descriptor [Soybeans] this term only
53. gluten*:ab,ti
54. MeSH descriptor [Glutens] this term only
55. fish:ab,ti
56. MeSH descriptor [Fishes] this term only
57. 1 or 2 or 3 or 4 or 5 or 6 or 7 or 8 or 9 or 10 or 11 or 12 or 13 or 14 or 15 or 16 or 17 or 18 or 19 or 20 or 21 or 22 or 23 or 24 or 25 or 26 or 27 or 28 or 29 or 30 or 31 or 32 or 33 or 34 or 35 or 36 or 37 or 38 or 39 or 40 or 41 or 42 or 43 or 44 or 45 or 46 or 47 or 48 or 49 or 50 or 51 or 52 or 53 or 54 or 55 or 56

© 2023 Scarpone R et al. *JAMA Pediatrics*.

58. allerg*:ab,ti
59. asthma*:ab,ti
60. wheeze:ab,ti
61. wheezing:ab,ti
62. “bronchial hyperresponsiveness”:ab,ti
63. “bronchial hyperreactivity”:ab,ti
64. “Forced expiratory volume”:ab,ti
65. “FEV1”:ab,ti
66. "FEV 1":ab,ti
67. "FEV0.5":ab,ti
68. "FEV 0.5":ab,ti
69. “Forced vital capacity”:ab,ti
70. FVC:ab,ti
71. “Peak expiratory flow rate”:ab,ti
72. PEFR:ab,ti
73. eczema:ab,ti
74. neurodermatitis:ab,ti
75. rhinitis:ab,ti
76. “besniers prurigo”:ab,ti
77. rhinoconjunctivitis:ab,ti
78. hayfever:ab,ti
79. “hay fever”:ab,ti
80. poll*nosis:ab,ti
81. SAR:ab,ti
82. “pollen allergy”:ab,ti
83. conjunctivitis:ab,ti
84. “immunoglobulin e”:ab,ti
85. “Total IgE”:ab,ti
86. “atopic disease”:ab,ti
87. “atopic dermatitis”:ab,ti
88. (food* NEAR/3 sensiti*):ab,ti
89. (food* NEAR/3 toleran*):ab,ti
90. (food* NEAR/3 intoleran*):ab,ti
91. ((aero or air*) NEAR/3 allergen*):ab,ti
92. (aeroallergen* NEAR/3 sensiti*):ab,ti
93. (allergen* NEAR/3 sensiti*):ab,ti
94. “skin prick test*”:ab,ti
95. atopy:ab,ti
96. hypersensitiv*:ab,ti
97. MeSH descriptor [Hypersensitivity] this term only
98. MeSH descriptor [Food Hypersensitivity] explode all trees
99. MeSH descriptor [Respiratory Hypersensitivity] this term only
100. MeSH descriptor [Asthma] this term only
101. MeSH descriptor [Bronchial Hyperreactivity] this term only
102. MeSH descriptor [Forced Expiratory Volume] this term only
103. MeSH descriptor [Vital Capacity] this term only
104. MeSH descriptor [Peak Expiratory Flow Rate] this term only
105. MeSH descriptor [Eczema] this term only
106. MeSH descriptor [Neurodermatitis] this term only
107. MeSH descriptor [Rhinitis] this term only
108. MeSH descriptor [Rhinitis, Allergic, Perennial] this term only
109. MeSH descriptor [Rhinitis, Allergic, Seasonal] this term only
110. MeSH descriptor [Conjunctivitis] this term only
111. MeSH descriptor [Immunoglobulin E] this term only
112. MeSH descriptor [Dermatitis, Atopic] this term only
113. MeSH descriptor [Hypersensitivity, Immediate] this term only
114. 58 or 59 or 60 or 61 or 62 or 63 or 64 or 65 or 66 or 67 or 68 or 69 or 70 or 71 or 72 or 73 or 74 or 75 or 76 or 77 or 78 or 79 or 80 or 81 or 82 or 83 or 84 or 85 or 86 or 87 or 88 or 89 or 90 or 91 or 92 or 93 or 94 or 95 or 96 or 97 or 98 or 99 or 100 or 101 or 102 or 103 or 104 or 105 or 106 or 107 or 108 or 109 or 110 or 111 or 112 or 113

© 2023 Scarpone R et al. *JAMA Pediatrics*.

115. infant*:ab,ti
116. ((one or two or three or four or five or six or seven or eight or nine or ten or eleven or twelve or thirteen or fourteen or fifteen or sixteen or seventeen or eighteen or nineteen or twenty or "twenty one" or "twenty two" or "twenty three" or "twenty four" or "twenty five" or "twenty six") NEAR/1 week*):ab,ti
117. ((one or two or three or four or five or six or seven or eight or nine or ten or eleven or twelve or thirteen or fourteen or fifteen or sixteen or seventeen or eighteen or nineteen or twenty or "twenty one" or "twenty two" or "twenty three" or "twenty four") NEAR/1 month*):ab,ti
118. ((1 or 2 or 3 or 4 or 5 or 6 or 7 or 8 or 9 or 10 or 11 or 12 or 13 or 14 or 15 or 16 or 17 or 18 or 19 or 20 or 21 or 22 or 23 or 24 or 25 or 26) NEAR/1 week*):ab,ti
119. ((1 or 2 or 3 or 4 or 5 or 6 or 7 or 8 or 9 or 10 or 11 or 12 or 13 or 14 or 15 or 16 or 17 or 18 or 19 or 20 or 21 or 22 or 23 or 24) NEAR/1 month*):ab,ti
120. 116 or 117 or 118 or 119
121. (old or age*):ab,ti
122. 120 and 121
123. (("one year*" or "two year*") NEAR/3 (old or age*)):ab,ti
124. ((first or second or two) NEAR/3 "year* of life"):ab,ti
125. (("1 year?" or "2 year?") NEAR/3 (old or age*)):ab,ti
126. ((first or second or 2) NEAR/3 "year* of life"):ab,ti
127. MeSH descriptor [Infant] this term only
128. MeSH descriptor [Infant, Newborn] this term only
129. 115 or 122 or 123 or 124 or 125 or 126
130. “clinical trial*”
131. random*
132. factorial*
133. crossover*
134. placebo*
135. “doubl* blind*”
136. “singl* blind*”
137. assign*
138. volunteer*
139. longitudinal*
140. follow-up
141. groups:ab.ti
142. MeSH descriptor [clinical trial] explode all trees
143. MeSH descriptor [Cross-Over Studies] this term only
144. MeSH descriptor [Placebos] this term only
145. MeSH descriptor [Double-Blind Method] this term only
146. MeSH descriptor [Single-Blind Method] this term only
147. 130 or 131 or 132 or 133 or 134 or 135 or 136 or 137 or 138 or 139 or 140 or 141 or 142 or 143 or 144 or 145 or 146
148. 57 and 114 and 129 and 147

© 2023 Scarpone R et al. *JAMA Pediatrics*.

**Medline**

1. complementary food?.ab,ti.
2. (introduc$ adj2 food?).ab,ti.
3. wean$.ab,ti.
4. Weaning/
5. solid?.ab,ti.
6. semi-solid?.ab,ti.
7. baby food?.ab,ti.
8. Infant Food/
9. Infant Nutritional Physiological Phenomena/
10. breast feeding.ab,ti.
11. breastfeeding.ab,ti.
12. breast fed.ab,ti.
13. breastfed.ab,ti.
14. Breast Feeding/
15. Milk, Human/
16. formula?.ab,ti.
17. hydrolysed.ab,ti.
18. bottlefed.ab,ti.
19. bottle fed.ab,ti.
20. (bottle adj3 feed$).ab,ti.
21. Infant Formula/
22. Bottle Feeding/
23. liquid?.ab,ti.
24. milk.ab,ti.
25. Milk/
26. egg?.ab,ti.
27. Egg Proteins/
28. Egg Proteins, Dietary/
29. nut?.ab,ti.
30. peanut?.ab,ti.
31. almond?.ab,ti.
32. (brazil? adj5 nut?).ab,ti.
33. walnut?.ab,ti.
34. pecan?.ab,ti.
35. pistachio?.ab,ti.
36. cashew?.ab,ti.
37. hazelnut?.ab,ti.
38. macadamia?.ab,ti.
39. Nuts/
40. Arachis hypogaea/
41. Prunus/
42. Bertholletia/
43. Juglans/
44. Carya/
45. Pistacia/
46. Anacardium/
47. Corylus/
48. Macadamia/
49. wheat.ab,ti.
50. Triticum/
51. soya.ab,ti.
52. Soybeans/
53. gluten$.ab,ti.
54. Glutens/
55. fish.ab,ti.
56. Fishes/
57. 1 or 2 or 3 or 4 or 5 or 6 or 7 or 8 or 9 or 10 or 11 or 12 or 13 or 14 or 15 or 16 or 17 or 18 or 19 or 20 or 21 or 22 or 23 or 24 or 25 or 26 or 27 or 28 or 29 or 30 or 31 or 32 or 33 or 34 or 35 or 36 or 37 or 38 or 39 or 40 or 41 or 42 or 43 or 44 or 45 or 46 or 47 or 48 or 49 or 50 or 51 or 52 or 53 or 54 or 55 or 56

© 2023 Scarpone R et al. *JAMA Pediatrics*.

58. allerg$.ab,ti.
59. asthma$.ab,ti.
60. wheeze.ab,ti.
61. wheezing.ab,ti.
62. bronchial hyperresponsiveness.ab,ti.
63. bronchial hyperreactivity.ab,ti.
64. Forced expiratory volume.ab,ti.
65. FEV1.ab,ti.
66. "FEV 1".ab,ti.
67. "FEV0.5".ab,ti.
68. "FEV 0.5".ab,ti.
69. Forced vital capacity.ab,ti.
70. FVC.ab,ti.
71. Peak expiratory flow rate.ab,ti.
72. PEFR.ab,ti.
73. eczema.ab,ti.
74. neurodermatitis.ab,ti.
75. rhinitis.ab,ti.
76. besniers prurigo.ab,ti.
77. rhinoconjunctivitis.ab,ti.
78. hayfever.ab,ti.
79. (hay adj fever).ab,ti.
80. poll?nosis.ab,ti.
81. SAR.ab,ti.
82. (pollen adj allergy).ab,ti.
83. conjunctivitis.ab,ti.
84. immunoglobulin e.ab,ti.
85. Total IgE.ab,ti.
86. atopic disease.ab,ti.
87. atopic dermatitis.ab,ti.
88. (food? adj3 sensiti$).ab,ti.
89. (food? adj3 toleran$).ab,ti.
90. (food? adj3 intoleran$).ab,ti.
91. ((aero or air$) adj3 allergen?).ab,ti.
92. (aeroallergen? adj3 sensiti$).ab,ti.
93. (allergen? adj3 sensiti$).ab,ti.
94. skin prick test$.ab,ti.
95. atopy.ab,ti.
96. hypersensitiv$.ab,ti.
97. Hypersensitivity/
98. exp Food Hypersensitivity/
99. Respiratory Hypersensitivity/
100. Asthma/
101. Bronchial Hyperreactivity/
102. Forced Expiratory Volume/
103. Vital Capacity/
104. Peak Expiratory Flow Rate/
105. Eczema/
106. Neurodermatitis/
107. Rhinitis/
108. Rhinitis, Allergic, Perennial/
109. Rhinitis, Allergic, Seasonal/
110. Conjunctivitis/
111. Immunoglobulin E/
112. Dermatitis, Atopic/
113. Hypersensitivity, Immediate/
114. 58 or 59 or 60 or 61 or 62 or 63 or 64 or 65 or 66 or 67 or 68 or 69 or 70 or 71 or 72 or 73 or 74 or 75 or 76 or 77 or 78 or 79 or 80 or 81 or 82 or 83 or 84 or 85 or 86 or 87 or 88 or 89 or 90 or 91 or 92 or 93 or 94 or 95 or 96 or 97 or 98 or 99 or 100 or 101 or 102 or 103 or 104 or 105 or 106 or 107 or 108 or 109 or 110 or 111 or 112 or 113

© 2023 Scarpone R et al. *JAMA Pediatrics*.

115. infant? ab.ti
116. ((one or two or three or four or five or six or seven or eight or nine or ten or eleven or twelve or thirteen or fourteen or fifteen or sixteen or seventeen or eighteen or nineteen or twenty or "twenty one" or "twenty two" or "twenty three" or "twenty four" or "twenty five" or "twenty six") adj week?).ab,ti.
117. ((one or two or three or four or five or six or seven or eight or nine or ten or eleven or twelve or thirteen or fourteen or fifteen or sixteen or seventeen or eighteen or nineteen or twenty or "twenty one" or "twenty two" or "twenty three" or "twenty four") adj month?).ab,ti.
118. ((1 or 2 or 3 or 4 or 5 or 6 or 7 or 8 or 9 or 10 or 11 or 12 or 13 or 14 or 15 or 16 or 17 or 18 or 19 or 20 or 21 or 22 or 23 or 24 or 25 or 26) adj week?).ab,ti.
119. ((1 or 2 or 3 or 4 or 5 or 6 or 7 or 8 or 9 or 10 or 11 or 12 or 13 or 14 or 15 or 16 or 17 or 18 or 19 or 20 or 21 or 22 or 23 or 24) adj month?).ab,ti.
120. 116 or 117 or 118 or 119
121. (old or age?).ab,ti.
122. 120 and 121
123. (("one year?" or "two year?") adj3 (old or age?)).ab,ti.
124. ((first or second or two) adj3 "year? of life").ab,ti.
125. (("1 year?" or "2 year?") adj3 (old or age?)).ab,ti.
126. ((first or second or 2) adj3 "year? of life").ab,ti.
127. Infant/
128. Infant, Newborn/
129. 115 or 122 or 123 or 124 or 125 or 126 or 127 or 128
130. clinical trial?.mp.
131. random$.mp.
132. factorial$.mp.
133. crossover$.mp.
134. placebo$.mp.
135. (doubl$ adj blind$).mp.
136. (singl$ adj blind$).mp.
137. assign$.mp.
138. volunteer$.mp.
139. longitudinal$.mp.
140. follow-up.mp.
141. groups.ab.ti.
142. exp clinical trial/
143. Cross-Over Studies/
144. Placebos/
145. Double-Blind Method/
146. Single-Blind Method/
147. 130 or 131 or 132 or 133 or 134 or 135 or 136 or 137 or 138 or 139 or 140 or 141 or 142 or 143 or 144 or 145 or 146
148. 57 and 114 and 129 and 147

© 2023 Scarpone R et al. *JAMA Pediatrics*.
