# Supplementary material for: Timing of Allergenic Food Introduction and Risk of Immunoglobulin E–Mediated Food Allergy: A Systematic Review and Meta-analysis
Source: JAMA Pediatr. 2023 Mar 27;177(5):489–97. doi: 10.1001/jamapediatrics.2023.0142 (PMC10043805; doi:10.1001/jamapediatrics.2023.0142)
Supplement: Supplement 2. — Data Sharing Statement [file jamapediatr-e230142-s002.pdf]

Data Sharing Statement

Scarpone. Timing of Allergenic Food Introduction and Risk of Immunoglobulin E-Mediated Food Allergy. *JAMA Pediatr*. Published March 27, 2023. doi:10.1001/jamapediatrics.2023.0142

Data
Data available: Yes
Data types: Deidentified participant data
How to access data: All data used for this systematic review and meta-analysis are in the public domain, but a copy of the specific datasets used for meta-analyses can be accessed by contacting the corresponding author.
When available: With publication

Supporting Documents
Document types: None

Additional Information
Who can access the data: Anyone requesting the data
Types of analyses: For any purpose
Mechanisms of data availability: With investigator support
Any additional restrictions: Nil
